# Identification and validation of quantitative real-time reverse transcription PCR reference genes for gene expression analysis in teak (*Tectona grandis* L.f.)

**DOI:** 10.1186/1756-0500-7-464

**Published:** 2014-07-22

**Authors:** Esteban Galeano, Tarcísio Sales Vasconcelos, Daniel Alves Ramiro, Valentina de Fátima De Martin, Helaine Carrer

**Affiliations:** 1 Departamento de Ciências Biológicas, Escola Superior de Agricultura “Luiz de Queiroz”, Universidade de São Paulo, Av. Pádua Dias, 11, Piracicaba, SP 13418-900, Brazil

**Keywords:** Relative expression, Trees, Transcript stability, Lignin

## Abstract

**Background:**

Teak (*Tectona grandis* L.f.) is currently the preferred choice of the timber trade for fabrication of woody products due to its extraordinary qualities and is widely grown around the world. Gene expression studies are essential to explore wood formation of vascular plants, and quantitative real-time reverse transcription PCR (qRT-PCR) is a sensitive technique employed for quantifying gene expression levels. One or more appropriate reference genes are crucial to accurately compare mRNA transcripts through different tissues/organs and experimental conditions. Despite being the focus of some genetic studies, a lack of molecular information has hindered genetic exploration of teak. To date, qRT-PCR reference genes have not been identified and validated for teak.

**Results:**

Identification and cloning of nine commonly used qRT-PCR reference genes from teak, including *ribosomal protein 60s* (*rp60s*)*, clathrin adaptor complexes medium subunit family* (*Cac*)*, actin* (*Act*)*, histone 3* (*His3*)*, sand family* (*Sand*)*, β-Tubulin* (*Β-Tub*)*, ubiquitin* (*Ubq*)*, elongation factor 1-α* (*Ef-1α*)*,* and *glyceraldehyde-3-phosphate dehydrogenase* (*GAPDH*). Expression profiles of these genes were evaluated by qRT-PCR in six tissue and organ samples (leaf, flower, seedling, root, stem and branch secondary xylem) of teak. Appropriate gene cloning and sequencing, primer specificity and amplification efficiency was verified for each gene. Their stability as reference genes was validated by NormFinder, BestKeeper, geNorm and Delta Ct programs. Results obtained from all programs showed that *TgUbq* and *TgEf-1α* are the most stable genes to use as qRT-PCR reference genes and *TgAct* is the most unstable gene in teak. The relative expression of the teak *cinnamyl alcohol dehydrogenase* (*TgCAD*) gene in lignified tissues at different ages was assessed by qRT-PCR, using *TgUbq* and *TgEf-1α* as internal controls. These analyses exposed a consistent expression pattern with both reference genes.

**Conclusion:**

This study proposes a first broad collection of teak tissue and organ mRNA expression data for nine selected candidate qRT-PCR reference genes. NormFinder, Bestkeeper, geNorm and Delta Ct analyses suggested that *TgUbq* and *TgEf-1α* have the highest expression stability and provided similar results when evaluating *TgCAD* gene expression, while the commonly used *Act* should be avoided.

## Background

The flux of information from DNA to protein is connected by mRNA, and the level of mRNA transcription is one of the factors determining the degree of gene expression [1]. Changes in gene expression are critical for cell development [2], integration of metabolism [3] and resistance to biotic and abiotic stresses [4,5], and as such are a research area of great interest to the fields of medicine, pharmacy, life sciences and agronomy.

Methods currently available for gene expression assessment include microarray analysis, Northern blotting, *in situ* hybridization, RNase protection assay, RNA sequencing (RNA-seq), qualitative RT-PCR, competitive RT-PCR, and quantitative real-time reverse transcription RT-PCR (qRT-PCR). The qRT-PCR is considered an efficient, safe (free of radioactive reagents), fast, affordable, reproducible, reliable and specific for quantifying levels of transcripts [6]. However, some variables such as the integrity, amount and purity of the RNA used as well as enzyme efficiency during cDNA synthesis and PCR amplification make an additional step to normalize the data necessary [7]. Normalization requires the use of one or more reference genes (also called internal control genes) for which expression is constant and stable at different developmental stages, nutritional conditions or experimental conditions [8]. Unfortunately, a gene has not been found for which expression is absolutely stable under all circumstances or across species that can be used indiscriminately for qRT-PCR analysis.

Bioinformatics tools have been developed to assess and identify the most suitable reference genes for qRT-PCR data normalization. geNorm shows expression stability throughout a set of housekeeping candidates [9], the Normfinder algorithm chooses the best candidate reference genes according to its calculations [10], while the Excel-based tool called BestKeeper determines the best candidate of pair-wise correlations [11]. Other statistical approaches used include Delta Ct [12] and “Stability index” methods [13].

Reference genes commonly used that present sufficiently stable expression are those related to cell maintenance such as *actin*, *tubulin*, *glyceraldehyde-3-phosphate dehydrogenase*, *elongation factor 1-α* and *18S ribosomal RNA*[8,14]. New genes have been studied as internal controls in model or commercial plants, such as *Arabidopsis*[15], *Populus*[16] and *Brachypodium*[17]. Tests for the selection of reference genes for qRT-PCR in teak have not been published yet.

Teak is a deciduous tree, native to countries of southeast Asia such as Myanmar, Thailand, India, Laos and Java [18]. Its wood is known internationally for its beauty, weightlessness, durability and weather resistance and it is used in the building of ships, furniture, house floors and walls, and general carpentry [19,20]. Currently, the wood market has a great interest in teak extractives such as naphthoquinones and anthraquinones, which have shown remarkable antifungal and antitermitic effect [21,22]. Additionally, teak populations serve significant environmental roles, as they can be used in agroforestry systems and forest recovery [22]. These characteristics make teak one of the most widely grown and economically profitable trees around the world [23]. Despite the great economic importance of teak, there are no studies of gene expression, the genome sequence is not available and sequenced genes are limited.

To select suitable qRT-PCR internal control genes for teak, this study analyzed the expression levels of candidate reference genes in different tissues and organs such as leaves, flowers, seedlings, roots, stem and branch secondary xylem of trees. Eight candidate reference genes were identified by their orthologous genes in model plants. These candidates were cloned, sequenced and tested. The selected genes are involved in different biological functions such as the formation of cellular cytoskeleton (*Actin and β-Tubulin*), elongation phase of translation (*Elongation factor 1-α*), DNA packaging (*Histone 3*) protein modification (*Ubiquitin*), intracellular transport (*Clathrin adaptor complexes medium subunit family*), vesicular transport (*SAND family*), protein biosynthesis (*Ribosomal protein 60s*) and carbohydrate metabolism (*Glyceraldehyde-3-phosphate dehydrogenase*).

Finally, in order to validate our results, the most stable reference genes were used to assess the *TgCAD* gene expression levels in different tissues and organs.

## Results

**Figure 1:**
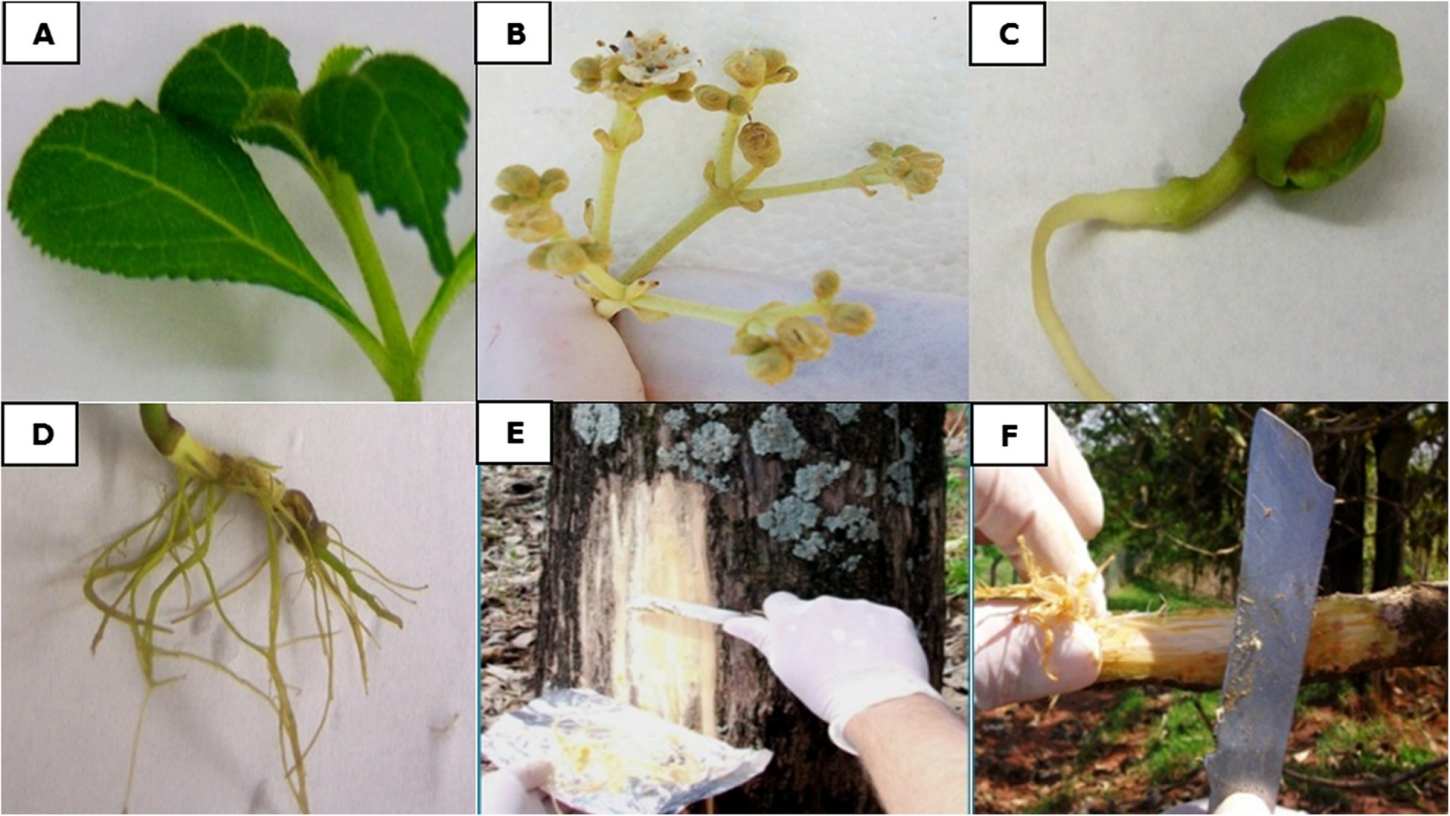
**Teak tissue and organ sample set. A** = leaf, **B** = flower, **C** = seedling, **D** = root, **E** = stem secondary xylem, **F** = branch secondary xylem.

Normalization of gene expression experiments, especially of qRT-PCR using a set of reference genes is currently a critical procedure when analyzing expression levels of target genes in different tissues or under different conditions. In the present study, nine potential reference genes for qRT-PCR of teak were assessed. A total of 36 cDNA samples including several organ types (leaf, root, and flower) and secondary xylem tissues from stems and branches of different ages were analyzed (Figure 1).

### Identification and cloning of references genes in teak

As teak does not have the relevant genetic sequence information available in databases, it was necessary to design degenerate primers to amplify, clone and sequence the reference genes according to the most common genes used for qRT-PCR analysis in trees such as *Platycladus orientalis*[24], *Vernicia fordii*[25], *Quercus suber*[26], *Populus euphratica*[27] and *Pyrus pyrifolia*[28]. *GAPDH* (FN431982.1) was the only teak sequence available in GenBank (http://www.ncbi.nlm.nih.gov/genbank). Therefore, we performed multiple nucleotide sequence alignment of the reference genes of different species for the remaining genes (Additional file 1). For each gene, at least four sequences were used in the alignments. Degenerate primers were designed to amplify the most conserved domains and at least 250 bp of the teak cDNA (Table 1, Additional file 2).

**Table 1:** Candidate reference genes, primers used to amplify in teak and their PCR parameters

**Gene symbol**	**Gene name**	Primer sequences (5′-3′) forward/reverse*	**Tm (°C)**	**Amplicon (bp)**
*rp60s*	*Ribosomal protein 60S*	ATGGTGAAGTTCTTGAAGCC/TGGTTCTTTACCAAGCTC	55	399
*Cac*	*Clathrin adaptor complex*	AAGGATAACTTTGTCATTGT/TGGGAAATACATGAAGGCG	58	794
*Act*	*Actin*	GTTAGCAATTGGGATGATATGG/ATCCAGACACTGTACTTCCT	57	797
*His3*	*Histone 3*	AC**N**GGTGGAGTGAAGAAGCC/TCCTTGGGCATGAT**N**GT**N**AC	61	275
*Sand*	*Sand family protein*	ATATATTCCAGATATGGAGATGA/TA**Y**ATGAAATGCCAAAGTCCA	55	941
*Β-Tub*	*Β-Tubulin*	AC**N**CA**R**CAAATGTGGGATGC/TCCCCAGTGTACCA**R**TGCAA	60	335
*Ubq*	*Ubiquitin*	T**R**ACGGG**N**AAGACCATAAC/ACCTTCTT**N**TTCTTGTGCTT	56	271
*Ef-1α*	*Elongation factor 1- α*	CATCAACATTGTGGTCATTGG/CCAGA**N**CGCCTGTCAATCTTG	55	1095

Degenerate bases are indicated in bold.

*Degenerate nucleotides used in some primers: N = any base, Y = C or T, R = A or G.

The degenerate primers were able to produce specific amplicons ranging from 271 to 1440 bp using cDNA of teak leaves as template (Table 1). After gel purification, PCR fragments were cloned into the pJET1.2/Blunt vector (Thermo Scientific, USA) and transformed into *DH5α*™ competent cells (Life Technologies, USA). Recombinant colonies were selected to extract plasmid DNA for sequencing.

The teak nucleotide identities were checked by BLAST [29] against NCBI non redundant sequences (http://blast.ncbi.nlm.nih.gov) and the results showed that all the clones contained the expected fragments. The most conserved genes were *TgAct* and *TgEf-1α* with 92% of similarity, followed by *TgUbq* and *Tgβ-Tub* with 91% and 87%, respectively (data not shown). All genes showed at least 79% of similarity. Translated amino acid sequences were obtained by Expasy Translation Tool (http://web.expasy.org/translate/) and used to check for the presence of the expected domains in Pfam Database (http://pfam.sanger.ac.uk). Thereafter, teak amino acid sequences were compared against NCBI protein sequences with the algorithm tBLASTn (http://blast.ncbi.nlm.nih.gov). Results of the *in silico* analysis showed that all teak putative protein sequences possess the predicted domains, presenting high similarity with the selected reference genes (Additional file 3). At protein level, the most conserved genes were *TgAct*, *TgEf-1α, TgHis3, Tgβ-Tub and TgUbq* presenting 99% of similarity (data not shown).

### Primer specificity and PCR efficiency

**Figure 2:**
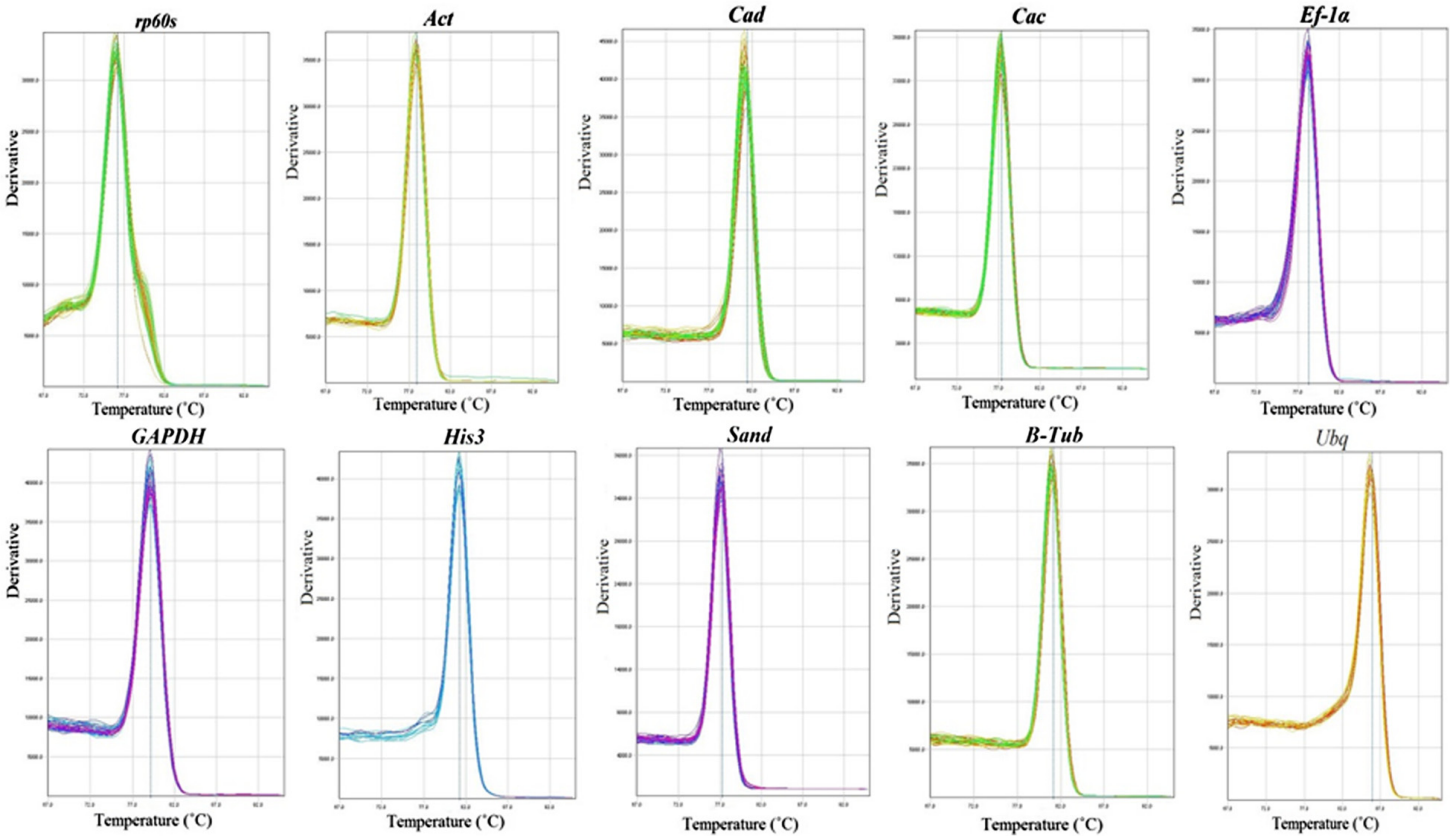
**Specificity of qRT-PCR amplification.** Melting curves (dissociation curves) of the 10 amplicons (*rp60s, Act, Cad, Cac, Ef-1α, GAPDH, His3, Sand, B-Tub, Ubq* genes) after the qRT-PCR reactions, all showing one peak.

Real-time PCR primers (Table 2, Additional file 4) were designed to amplify the teak sequences of the eight clones (*Tgrp60s, TgCac, TgAct, TgHis3, TgSand, Tgβ-Tub, TgUbq and TgEf-1α*) and *TgGAPDH* (Table 2), and were used to detect transcript levels. Primer specificity was evaluated with a single peak in all ten melting curves (Figure 2) and as a single band in the agarose gel analysis (Additional file 5). qRT-PCR efficiency (E) varied from 91.4% for *Tgrp60s* to 108.5% for *TgSand* and correlation coefficients (R^2^) oscillated from 98.2% for *TgΒ-Tub* to 99.9% for *TgEf-1α* (Table 2). The acceptable range for PCR efficiencies calculated using standard curve serial dilution experiments is 90–110% (i.e. a slope between 3.1 and 3.58) [30]. The annealing temperature of 65°C was effective for all primers; nevertheless, its choice can impact on the efficiency of the reaction. Altogether, the results showed that the chosen primers accurately amplified the candidate reference genes.

**Table 2:** **Candidate reference genes, *TgCAD *** **target gene, specific qRT-PCR primers and different parameters derived from qRT-PCR analysis**

**Gene symbol**	**Accession number**	**Primer sequences (5′-3′) forward/reverse**	**Tm (°C)**	**Amplicon length (bp)**	**Primer efficiency**	**R**^**2**^
*Tgrp60s*	JZ515972	AGAAGCAGGCGAAGAAATCA/GTGGGCATGATGTGGTTGTA	75.9	70	91.4	0.998
*TgCac*	JZ515973	ATCTTGTGGAAGAAATGGATGC/TTCGCAAACAACAGAGTGAGAT	77.4	127	91.7	0.994
*TgAct*	JZ515974	TCCAGAAGAGCACCCAATTC/CAGGGGCATTAAAGGTCTCA	77.9	100	91.6	0.995
*TgHis3*	JZ515975	TGGCTTTGGAACCTCAAATC/CCCTGGAACTGTTGCTCTTC	81.2	135	92.4	0.998
*TgSand*	JZ515976	GCCCAAAAAGCATCTCTTCA/TTGTGGTGAGCAAGATCAGG	77.1	187	108.5	0.987
*TgΒ-Tub*	JZ515977	CAAGATGAGCACGAAAGAAGTG/CGGAACATCTCCTGTATCGAC	81.1	180	93.8	0.982
*TgUbq*	JZ515978	CGGGTAAGACCATAACTCTGGA/GTCGATTCCTTTTGGATGTTGT	85.6	171	92.8	0.998
*TgEf-1α*	JZ515979	ACCACACCAAAATACTCCAAGG/TGGACCTCTCAATCATGTTGTC	78.1	145	93.5	0.999
*TgGAPDH*	FN431983.1	GGCCACCTATGAGGAGATCA/CCAAGATGCCCTTTAGCTTG	79.2	152	101.9	0.998
*TgCAD*	JZ515980	CGGCAAGGTCTACAAAGGAG/GGCTGTTTATCGCTTGCTTC	78.8	200	98.4	0.993

**Figure 3:**
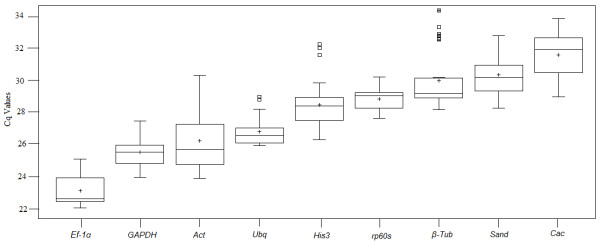
**Expression levels of candidate reference genes in different plant samples.** Expression data displayed as Cq values for each reference gene in all *T. grandis* samples. The horizontal lines of the box indicate the 25th and 75th quartiles. The central horizontal line across the box is depicted as the median. The whisker caps represent the maximum and minimum values. Dots represent outliers. Genes are in order from the most (lower Cq, on the left) to the least abundantly expressed (higher Cq, on the right).

To compare the differences in transcript levels between reference genes, the Cq range was determined and the coefficient of variance was calculated for each gene across all samples based on the interquartile range (25-75% percentiles). The average Cq values of the different genes ranged from 22 to 34 cycles (Figure 3, Table 3). *TgUbq, Tgβ-Tub* and *Tgrp60s* showed the narrowest variance (lowest Cq dispersion), while *TgAct* and *TgCac* exhibited widest variance (highest dispersion). The gene with the most abundant transcript level was *TgEf-1α* while *TgCac* was the least abundant, reaching mean threshold fluorescence with 23 and 31 amplification cycles, respectively.

**Table 3:** Descriptive statistics and expression level obtained by BestKeeper

**Factor**	***Tgrp60s***	***TgSand***	***TgAct***	***TgCac***	***TgGAPDH***	***TgΒ-Tub***	***TgHis3***	***TgUbq***	***TgEf-1α***
N	36	36	36	36	36	36	36	36	36
GM [CP]	28.59	29.92	26.21	31.05	25.63	29.59	28.24	26.77	23.45
AM [CP]	28.59	29.94	26.27	31.07	25.64	29.62	28.27	26.78	23.47
Min [CP]	27.52	28.08	24.15	28.71	24.21	28.00	26.31	25.99	22.50
Max [CP]	29.82	32.15	29.91	33.11	27.36	33.54	31.66	28.71	25.23
SD [±CP]	**0.51**	**0.90**	**1.42**	**0.99**	**0.64**	**1.21**	**1.02**	**0.60**	**0.73**
CV [%CP]	**1.78**	**3.01**	**5.40**	**3.20**	**2.49**	**4.10**	**3.62**	**2.23**	**3.12**
Min [x-fold]	-1.99	-3.82	-3.84	-4.58	-2.72	-2.62	-3.53	-1.63	-1.79
Max [x-fold]	2.22	5.13	11.12	3.85	3.38	10.89	9.29	3.37	2.99
SD [±x-fold]	1.39	1.79	2.50	1.90	1.51	2.19	1.94	1.47	1.61

Abbreviations: N: number of samples; CP: crossing point; GM [CP]: geometric CP mean; AM [CP]:arithmetic CP mean; Min [CP] and Max [CP]: CP threshold values; SD [±CP]: CP standard deviation; CV [%CP]: variance coefficient expressed as percentage of CP level; Min [x-fold] and Max [x-fold]: threshold expression levels expressed as absolute x-fold over- or under-regulation coefficient; SD [±x-fold]: standard deviation of absolute regulation coefficient.

SD and CV are indicated in bold.

### Expression stability of the nine candidate reference genes

To evaluate the reference genes’ expression stability, four different methodologies were used: geNorm, NormFinder, BestKeeper and Delta Ct.

### geNorm

**Figure 4:**
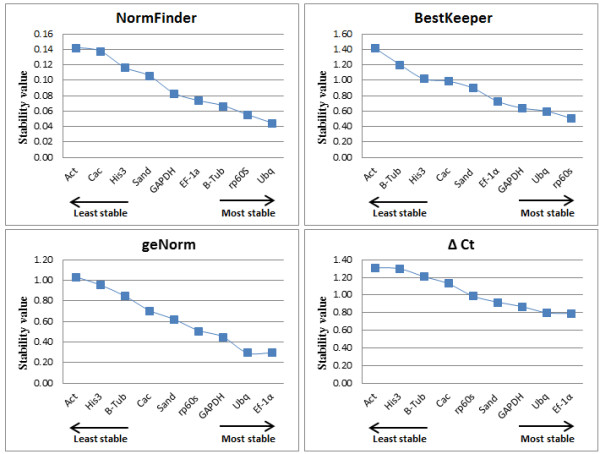
**Gene expression stability of the candidate reference genes calculated by different statistical methods.** Ranking of each candidate reference gene (*rp60s, Act, Cac, Ef-1α, GAPDH, His3, Sand, B-Tub, Ubq*) calculated by NormFinder, BestKeeper, geNorm and Delta Ct methods, for all tested cDNA samples (leaf, flower, seedling, root, stem secondary xylem, branch secondary xylem).

geNorm was used to rank the reference genes by calculating the gene expression stability value *M,* which corresponds to the average pairwise variation (V) of a particular gene with all other control genes [9]. The most stable reference gene has the lowest *M* value, while the least stable has the highest *M* value. To identify reference genes with stable expression, geNorm indicates genes with *M* values below the threshold of 1.5, however Vandesompele et al. [9] suggests *M* values lower than 1.0 to ensure the selection of the most stable genes. When all 36 samples were analyzed together with geNorm (Figure 4), eight genes had *M* < 1.0, with *TgUbq* and *TgEF1α* showing the highest expression stability (*M* = 0.295) in different tissues. *Act* was the only gene with M > 1.0, with the lowest expression stability of 1.035.

**Figure 5:**
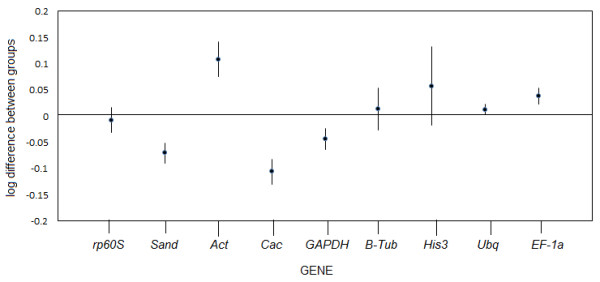
**Gene expression differences among the candidate reference genes analyzed by NormFinder.** Black circles represent the log-transformed gene expression levels. Vertical bars give a confidence interval for the inter-tissue variation. Top and bottom lines from the graphic represent the maximum standard deviation of the candidate reference genes, with the difference log expression levels between 0.2 and -0.2.

**Figure 6:**
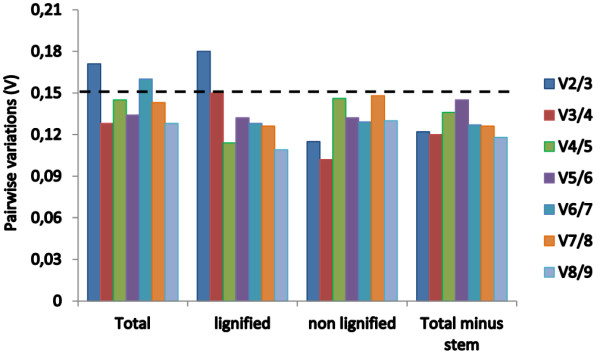
**Pairwise variations (V) calculated by geNorm to determine the optimal number of reference genes.** The average pairwise variations V_n/n+1_ was analyzed between the normalization factors NF_n_ and NF_n+1_ to indicate the optimal number of reference genes required for qRT-PCR data normalization in all the samples, lignified tissues (root, stem secondary xylem, branch secondary xylem), non-lignified tissues (leaf, flower, seedling) and in all samples minus stem secondary xylem.

To obtain reliable results from qRT-PCR studies, two or more reference genes should be used for data normalization. The optimal number of reference genes can be determined by calculating the pairwise variation (V_n/n+1_) using the geNorm algorithm [9]. It is calculated between the two sequential normalization factors (NF), NF_n_ and NF_n+1_, for all the samples analyzed. Slight variations mean addition of another gene has a low effect on the normalization. Vandesompele et al. [9] proposed 0.15 as the cut-off value for V, below which the inclusion of an additional control gene is not required. This means that if V_n/n+1_ < 0.15, it is not necessary to use ≥ n + 1 reference genes for normalization. In this study, the paired variable coefficients indicated that the inclusion of the third reference gene (i.e. *TgUbq*, *TgEF1α* and *TgGAPDH*) would be useful for normalization when considering total samples and only lignified samples, whereas two stable reference genes (*TgGAPDH* and *Tgrp60s*) can be employed when analyzing non-lignified tissues (Figure 5). However, if the samples of stem secondary xylem (the most lignified tissue) are excluded from the analysis, two reference genes (*TgUbq* and *TgEF1α*) would be optimal for normalizing gene expression (Figure 6).

### NormFinder

NormFinder is a Microsoft Excel-based Visual Basic application that allows estimation of stability values of single candidate reference genes. The algorithm is based on intra- and inter-group variations and combines both results into a stability value for each candidate reference gene [10]. The results of the NormFinder analysis were somewhat similar to those of geNorm. Both methods ranked *TgUbq*, *TgEF1α* and *Tgrp60s* as among the four most stable reference genes (Figure 5) and *TgAct* and *TgHis3* as the least stable (Figure 4). However, *Tgβ-Tub* emerged as the third most stably expressed using NormFinder, whereas it was ranked seventh by geNorm. These discrepant results could be explained due to inter-tissue expression variations detected by NormFinder analysis, which is not take account for gene stability calculations in the geNorm algorithm. When considering only intra-tissue variations, *Tgβ–Tub* was the most stable gene in lignified tissues (i.e. roots, branches and stems) (Table 4). However, in non-lignified tissues (leaves, flowers and seedlings) *Tgβ–Tub* was ranked eighth of nine genes, corroborating results obtained by geNorm.

**Table 4:** NormFinder intragroup expression stability for teak candidate reference genes

**Gene**	Non-lignified^*^	Lignified^**^	**Total**
*Tgrp60s*	0.032 (6)	0.016 (5)	0.048 (4)
*TgSand*	0.024 (5)	0.015 (4)	0.039 (3)
*TgAct*	0.043 (7)	0.023 (6)	0.066 (7)
*TgCac*	0.012 (2)	0.037 (8)	0.059 (6)
*TgGAPDH*	0.017 (3)	0.023 (6)	0.050 (5)
*TgΒ-Tub*	0.069 (8)	0.011 (1)	0.080 (8)
*TgHis3*	0.113 (9)	0.037 (8)	0.150 (9)
*TgUbq*	0.009 (1)	0.012 (2)	0.021 (1)
*TgEf-1α*^***+***^	0.017 (3)	0.014 (3)	0.031 (2)

Between parenthesis: ranking of stability.

*Column 1: non-lignified tissues (flower, leaf, seedling).

**Column 2: lignified tissues (root, branch, stem).

### BestKeeper

The Bestkeeper software was adopted for descriptive analysis. The program is an Excel-based software tool that estimates gene expression stability based on the coefficient of correlation (r) between each reference gene and an index, defined as the geometric mean of all candidate reference gene Ct (or CP) values [11]. The BestKeeper also calculates the CP standard deviation (SD) and the coefficient of variance (CV) of each candidate gene. Reference genes with SD values >1 are considered not stable and should be avoided. Results of analysis are shown in Table 3. Similarly to geNorm and NormFinder, BestKeeper ranked *Act* and *His3* among the three least stable reference genes with SD values >1.0 (1.42 and 1.02 respectively). In addition, *Tgβ–Tub* presented unstable expression with SD value of 1.21, being one of the least stable genes as observed in the geNorm analysis (Figure 4). The best reference genes are those that have the lowest coefficient of variance and standard deviation. In this study, *Tgrp60s* and *TgUbq* had CV ± SD values of 1.78 ± 0.51 and 2.23 ± 0.60, respectively, displaying a stable expression in all samples. The results of the BestKeeper analysis showed a similar pattern of stability to those obtained from geNorm, which described *TgUbq*, *Tgrp60s*, *TgEF1α* and *TgGAPDH* as the four best reference genes for the normalization of qRT-PCR data in teak. In the NormFinder analysis, *TgGAPDH* was replaced by *Tgβ–Tub* among the top four regarding stability (Figure 4).

### Delta Ct

The Delta Ct method is based on the ‘pairs of genes’ comparison using a simple ΔCt approach [12]. The formula used in this method is similar to the standard comparative Ct method (ΔΔCt) [31] except that no endogenous reference gene is incorporated since the purpose is to define stably expressed genes to normalize. In this approach, all pairs of genes are compared to each other and the genes are ranked according to the ΔCt values, from lowest to highest [12]. As observed in geNorm and Bestkeeper analysis, *TgEF1α, TgUbq* and *TgGAPDH* were the best reference genes, as they had the highest expression stability (lowest ΔCt values) (Figure 4). As was detected in all used programs, *TgHis3* and *TgAct* were the least stable internal controls for gene expression normalization.

### Validation of TgUbq and TgEF-1a as internal controls to assess expression of the teak cinnamyl alcohol dehydrogenase gene in lignified tissues

**Figure 7:**
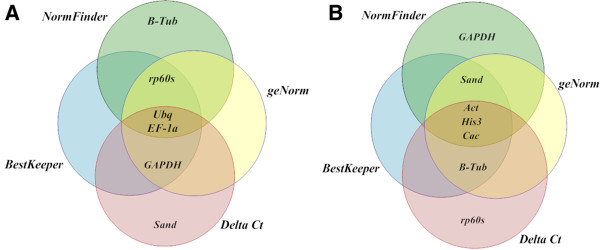
**Venn diagrams. (A)** the most stable reference genes present in the first four positions and **(B)** the least stable genes present in the last five positions identified by the NormFinder, BestKeeper, geNorm and Delta Ct methods. Diagrams were performed with the Smartdraw® program.

**Figure 8:**
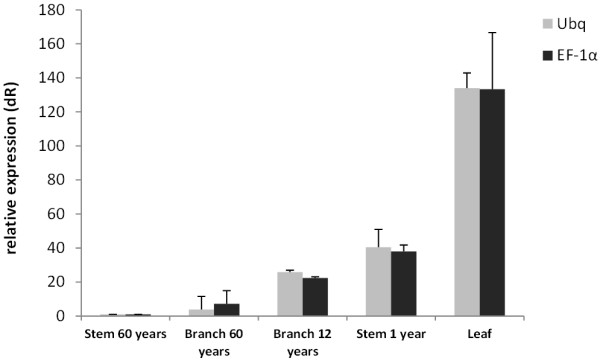
**Expression levels of the *TgCAD *gene.** It was used different tissues and organ ages of teak tree, using the best validated reference genes (*TgUbq* and *TgEF-1a*) for normalization and the results are represented as mean fold changes in relative expression compared to stem secondary xylem from 60 years-old trees. Bars are mean standard deviation calculated from the 3 biological replicates.

The use of different reference genes to evaluate relative expression data has an important impact on the final normalized results. As *TgUbq* and *TgEF-1a* showed the best stability values by geNorm, Delta Ct, NormFinder and BestKeeper analyses (Figure 7), they were used to evaluate the transcript level of a gene of interest, the teak *cinnamyl alcohol dehydrogenase* (*TgCAD*). *TgCAD* was identified and cloned using the same methodologies described for the reference genes. To validate the selected reference genes, the transcript levels were quantified in leaves from four month-old greenhouse grown teak, and in lignified tissues and organs such as stem secondary xylem from 60 year-old trees, stem from 1 year-old plants and branch secondary xylem from 60 and 12 year-old teak trees. Results showed that no matter which gene is used (*TgUbq* or *TgEF-1a*), *TgCAD* expression decreased in the following order: leaf > stem from 1 year-old plants > branch secondary xylem from 12 year-old trees > branch secondary xylem from 60 year-old trees > stem secondary xylem from 60 year-old trees (Figure 8). On the other hand, between tissues, leaf was the tissue with highest expression and stem and branch secondary xylem from 60 year-old teak trees were the tissues with lowest expression of the *TgCAD* gene.

## Discussion

In plant molecular biological research, qRT-PCR has improved the detection and quantification of expression profiles of target genes due to its sensitivity, specificity and accuracy. For correct qRT-PCR measurements, reference genes are used as endogenous controls for gene expression normalization when analyzing the expression of genes of interest [5,24]. Therefore, a careful choice of reference genes is essential to obtain an accurate quantification of the target gene transcript levels [25].

Currently, teak is one of the most important trees worldwide due to its wood’s properties. Despite the growing importance of teak wood and extractives to the world market, the number of studies adopting techniques of modern biology for teak improvement is still quite limited. As far as has been documented, this is the first study of cloning and expression stability of qRT-PCR reference genes in teak tissues. In total, eight candidate genes (*Tgrp60s*, *TgCac, TgAct, TgHis3, TgSand, TgΒ-Tub, TgUbq,* and *TgEf-1α*) were successfully identified.

One of the challenges of studying gene expression in trees is to ensure good quality of total RNA isolated from stems and branches, which are woody tissues with high lignin contents. In this study, the use of the Salzman protocol [32] for total RNA extraction, followed by a DNAse I (Promega) treatment, provided high quality RNA from all lignified tissues and was chosen as standard method for RNA extraction.

The stability of nine reference genes for qRT-PCR normalization was assessed by four statistical approaches, geNorm, NormFinder, BestKeeper and Delta Ct method, in teak lignified and non-lignified tissues at different developmental stages. In spite of some inconsistencies that are usually observed between these methods [24,26,33], our results were quite constant regardless of the algorithm used for analysis. When considering the rank of four most and least stable genes, *TgUbq* and *TgEF1α* were selected among the most stable in all methods, while *TgAct*, *TgHis3* and *TgCac* showed the least expression stability. The only clear discrepancy within the results was the inclusion of *Tgβ-Tub* in the most stable group by NormFinder and in the least stable group by the other programs (Figure 4). In the NormFinder intra-tissues analysis, *Tgβ–Tub* was the most stable gene in lignified tissues, whereas it was ranked eighth of the nine genes in non-lignified tissues (Table 4). These results suggest that *Tgβ–Tub* is a suitable reference gene in lignified tissues and could be used as internal control for quantifying gene expression in them.

Among recent studies in trees searching for suitable reference genes, control genes such as *Act*, *Ubq*, *Ef1α, α-Tub, Cac, Sand, β-Tub* were considered to be stable in various tissues and different conditions [24-28]. *Act*, *Ubq* and *Ef1α* were shown to be suitable reference genes for normalization in lignified tissues of *Vernicia fordii*[25] and *Quercus suber*[26]. In our analysis, the most stable genes were *Ubq* and *EF1α* (Figure 7).

Genes encoding elongation factor-1α and ubiquitin are frequently considered consistent reference genes under different experimental conditions. *EF1α* has been found to be one of the most stable reference genes in several plants and conditions such as *Nicotiana tabacum*[34], *Lolium perenne*[35] and *Capsicum annuum*[36]. The *Ubq* gene showed high stability for qRT-PCR normalization in *Platycladus orientalis*[24] and *Brachypodium distachyon*[37]. In combination, *Ubq* and *EF1α* showed stable expression across different tissues of *Vernicia fordii*[25] and *Dimocarpus longan*[33]. However, *Ef1α* and *Ubq* were the most variable reference genes in *Lycopersicum esculentum*[38] and *Euphorbia esula*[39], respectively, suggesting that these genes might not be suitable for qRT-PCR normalization in some plants and/or conditions. Although *Act* is one of the most commonly used reference gene in plants, in teak it showed low stability when assessed in different tissue samples and with different statistical methods. Similar results were observed in *Nicotiana tabacum* plants with viral infections [40] and *Glycine max*[41].

Studies have shown that the expression of reference genes can vary significantly under different experimental conditions [33,42]. To mitigate these variations, the use of multiple reference genes to assess target gene expression is appropriate. geNorm analysis of paired variable coefficients suggested the inclusion of a third reference gene (i.e. *TgUbq*, *TgEF1α* and *TgGAPDH*) for normalization when considering the total number of samples (Figure 6). Although the cut off value ≤0.15 is frequently used to confirm the optimal number of reference genes [9], this is not an absolute number because small datasets require fewer reference genes than larger ones and previous studies have reported proper normalization with higher cut-off values [40]. In this study, the combination of the two most stable reference genes (*TgUbq* and *TgEF1α*) to evaluate expression stability in all samples provided a coefficient of 0.17 and, thus, can be sufficient for the normalization of qRT-PCR data in teak, especially considering that the use of more than two reference genes in large scale gene expression profiles will significantly increase the costs of analysis.

To validate the utility of *TgUbq* and *TgEF-1a* as reference genes, the expression profile of *TgCAD* was assessed in teak leaves and lignified tissues collected from plants in the field at different development stages (Figure 8). *CAD* functions in one of the final steps of monolignol biosynthesis in the phenylpropanoid pathway and its study is essential to understand the lignin deposition and cell wall formation in trees. It catalyzes the NAPDH-dependent reduction of cinnamyl aldehydes to cinnamyl alcohols prior to their transport to the secondary cell wall for polymerization into the lignin heteropolymer [43]. In plants, *CAD* expression may vary according to tissue and development stage [44]. In addition, it has been shown that *CAD/CAD*-like genes are differentially expressed in plants infected with pests and pathogens [45,46].

Using *TgUbq* or *TgEF-1a* as reference genes, qRT-PCR results showed that *TgCAD* was strongly expressed in leaves (average 133-fold), followed by stems from 1 year-old plants (40-fold), branch secondary xylem from 12 year-old trees (24-fold), branch secondary xylem from 60 year-old trees (5-fold) and stem secondary xylem from 60 year-old trees (*calibrator*) (Figure 8). We observed higher expression of *TgCAD* in younger lignified tissues compared to older ones, probably due to less lignin deposition and secondary wall formation in 60 year-old trees. The *TgCAD* expression in lignified tissues and leaves presented the same pattern whichever internal control used, indicating that the reference genes identified in this study are suitable for qRT-PCR normalization in different tissues and plant ages.

This is the first attempt to identify qRT-PCR reference genes in several teak tissues. These results suggest the use of *TgUbq* and *TgEF-1a* as the best combination of reference genes for gene expression assessment in leaves, flowers, seedlings, roots, and lignified stem and branch secondary xylem of varying ages in teak. In addition, we recommend the researchers to validate the reference genes in their samples of interest before performing any experiment. The different tissues show that *TgAct* is not a suitable reference gene to normalize gene expression in this tree, highlighting the need to evaluate commonly used reference genes for particular species, conditions, tissues and organs. Finally, they advise that the use of reference genes without validation may reduce precision or produce misleading results.

## Conclusions

To the best of our knowledge, this study is the first attempt at cloning, sequencing and evaluating a set of commonly used candidate reference genes for the normalization of gene expression analysis using qRT-PCR in teak. Our data showed that expression stability varied considerably among the nine genes tested in the different samples of teak tissues. Stability analysis using NormFinder, Bestkeeper, geNorm and Delta Ct showed that *TgUbq* and *TgEF-1a* are the most stable genes across different tissues and organs, while *TgAct* was deemed to be unsuitable as a reference gene. *TgCAD* expression analyses confirmed *TgUbq* and *TgEF-1a* stability for correct normalization in teak. Consequently, they can be used in future gene expression studies of target genes in different teak tissues.

## Methods

### Plant material

Roots, seedlings and leaves were obtained from fifteen four month-old greenhouse grown teak. Flowers and branch and stem secondary xylem (Figure 1) were collected from fifteen twelve year-old teak trees located in Piracicaba, São Paulo State, Brazil. All the harvested tissues were immediately frozen by immersion in liquid nitrogen and stored at -80°C.

### Total RNA extraction, purification and quality controls

Frozen tissue samples of 1.0 g were weighed and ground to fine powder in liquid nitrogen using a sterilized mortar and pestle. The fifteen samples from each tissue or organ were divided into three different RNA extractions (five samples for each extraction). Total RNA was extracted following a protocol developed for lignified tissues by Salzman et al. [32]. RNA quality assessment included purity (absence of protein and DNA) and integrity (absence of RNA degradation). 1 μl of each extraction was analyzed spectrophotometrically using a Nanodrop ND-1000 Spectrophotometer (NanoDrop Technologies Inc., USA) and only RNA samples with 260/280 ratio between 1.9 and 2.1 and 260/230 ratio greater than 2.0 were used for subsequent analyses. The concentration of each sample was approximately 2 μg/μl, so they were diluted to a final concentration of 1 μg/μl and 4 μg of total RNA from each sample was treated with DNAse I (Promega). Then, 0.5 μl of each treated sample was analyzed in agarose gels, all displaying clear bands corresponding to rRNA, absence of DNA and no degradation. In addition, PCR control reactions to examine for genomic DNA contamination were performed using total RNA without reverse transcription as template, and negative results (absence of bands) were assessed by electrophoresis on a 1% (w/v) agarose gel with ethidium bromide staining.

### cDNA synthesis

Two cDNA samples were synthesized from the three extractions of each tissue or organ from 1.0 μg of the treated RNA using the SuperScript™ III First-Strand Synthesis System for RT-PCR (Invitrogen) according to the manufacturer’s instructions. Each cDNA sample concentration was determined using the Nanodrop ND-1000 Spectrophotometer (NanoDrop Technologies Inc., USA) to be approximately 2000 ng/μl. A concentration of 100 ng/μl (1:20 dilution) and 25 ng/μl (1:80 dilution) was used for PCR amplification and qRT-PCR expression experiments, respectively.

### Multiple sequence alignments, PCR and qRT-PCR primer design

Primers (Table 1) were manually designed flanking the conserved domains of *rp60s*, *Cac, Act, His3, Sand, Β-Tub, Ubq,* and *Ef-1α* after doing Clustal alignment (http://www.ebi.ac.uk/Tools/msa/clustalw2) of several orthologous plant sequences obtained from GenBank (http://www.ncbi.nlm.nih.gov/genbank) to amplify by PCR those genes from teak leaf cDNA (Additional file 2). The eight amplified fragments gel electrophoresis were excised, purified with Fragment CleanUp® (Invisorb, USA) and inserted into the pJET1.2/Blunt vector from the CloneJet™ PCR Cloning Kit (Thermo Scientific, USA) following the manufacturer’s recommendations. Plasmids were cloned in *DH5α*™ competent cells (Life Technologies, USA) and recombinant colonies were sequenced with the 3100 Genetic Analyzer (Applied Biosystems, USA) using pJET1.2/Blunt vector specific primers. Finally, sequences were blasted using blastx (http://blast.ncbi.nlm.nih.gov) to confirm their percentage amino acid similarity to the conserved domains and were translated (http://web.expasy.org/translate/) to amino acid sequences, which were submitted to PFAM search (http://pfam.sanger.ac.uk/search) (Sanger Institute, England) to confirm the presence of each gene’s canonical protein domains. The primers for qRT-PCR were designed flanking the eight cloned teak sequences and *GAPDH* (Table 2) with OligoPerfectTM Designer (Life technologies, USA) with default parameters. Teak candidate reference genes, *TgCAD* target gene, NCBI accession numbers, qRT-PCR primer information and different parameters derived from qRT-PCR analysis are shown in Table 2.

### Primer specificity, qRT-PCR Efficiency and R^2^

Confirmation of primer specificity was based on the dissociation curve at the end of each run (Figure 3). To determine the amplification efficiencies of the candidate genes, it was used cDNA samples from the teak leaf with five dilutions to obtain the standard curve, and then the PCR efficiency for each gene was calculated according to the equation (1 + E) = 10^slope^. The correlation coefficient (R^2^) and slope values were obtained from the standard curve (Table 2).

### Quantitative real-time reverse transcription PCR

The qRT-PCR mixture contained 5.0 μl of a 1:80 dilution of the six synthesized cDNAs from each tissue or organ, primers to a final concentration of 50 μM each, 12.5 μl of the SYBR Green PCR Master Mix (Applied Biosystems, USA) and PCR-grade water up to a total volume of 25 μl. Each gene reaction was performed in technical replicate. PCR reactions without template were also done as negative controls for each primer pair. The quantitative PCRs were performed employing the StepOnePlus™ System (Applied Biosystems, USA). All PCR reactions were performed under the following conditions: 2 min at 50°C, 2 min at 95°C, and 45 cycles of 15 s at 95°C and 1 min at 65°C in 96-well optical reaction plates (Applied Biosystems, USA). Leaf samples were used as calibrator to normalize the values between different plates.

### Analysis of gene expression stability

Gene expression stability was evaluated by applying four different statistical approaches: geNorm [9], NormFinder [10], Bestkeeper [11] and Delta Ct [12]. qRT-PCR data was exported from the StepOnePlus™ System (Applied Biosystems, USA) into an Excel datasheet (Microsoft Excel 2003) as Raw Crossing Point data (Additional file 6) and those values were log transformed by the 2^-ΔCt^ method for further requirements. Each of these approaches generated a measure of reference gene stability, by which each gene was ranked. Venn diagrams were constructed with the Smartdraw® program.

### Validation of reference genes

One gene of interest, putatively coding for a *cinammyl alcohol dehydrogenase* (*CAD*) (Table 2), an enzyme involved in lignin biosynthesis, one of the terminal steps of the phenylpropanoid pathway, was used to validate the best two reference genes. The relative expression level of the target gene was determined in leaves, and the lignified tissues of stem and branch secondary xylem of 60 year-old trees, stem of 1 year-old trees and branch secondary xylem of 12 year-old trees, expecting a higher expression level in younger tissues with a continuous secondary wall formation. The experimental procedure was the same used for the selection of the reference genes. Stem secondary xylem of 60 year-old tree samples were chosen as calibrator.

## Abbreviations

*Tg*: *Tectona grandis*; *rp60s*: *Ribosomal protein 60s*; Cac: Clathrin adaptor complexes medium subunit family; Act: Actin; His3: Histone 3; Sand: Sand family; Β-Tub: β-Tubulin; Ubq: Ubiquitin; Ef-1α: Elongation factor 1-α; GAPDH: Glyceraldehyde-3-phosphate dehydrogenase; CAD: Cinnamyl alcohol dehydrogenase; cDNA: Complementary DNA; ΔCt: Delta cycle threshold; mRNA: Messenger RNA; PCR: Polymerase chain reaction; qRT-PCR: Quantitative real-time reverse transcription PCR; CP: Crossing-point cycle number; GM: Geometric mean; AM: Arithmetic mean; SD: Standard deviation; CV: Coefficient of variation; bp: Base pairs; Tm: Melting temperature; R^2^: Correlation coefficient.

## Competing interests

The authors declare that they have no competing interests.

## Authors’ contributions

EG is the primary author of the manuscript. EG, TSV and DAR performed all the sample collection and preparation, experimental procedures, data analysis, qRT-PCR experiments and drafted the manuscript. VF and HC participated in the study design and provided helpful discussions and contributed with reagents, materials and analysis tools. EG, TSV, DAR and HC authors participated in the structuring and editing of the manuscript. HC coordinated the study, supervised the research and provided financial support. All authors read and approved the final manuscript.

## Supplementary Material

Additional file 1 Information related to the orthologous plant sequences used in this study. Click here for file

Additional file 2 **Clustal alignments used for designing primers to amplify orthologous sequences in teak.** Red and green squares mean forward and reverse primers, respectively. Click here for file

Additional file 3 Protein clustal alignments for teak candidate reference genes. Click here for file

Additional file 4 Teak sequences (with accession numbers) used for designing qRT-PCR primers (Underlined and bolded). Click here for file

Additional file 5 **Agarose gel (2%) electrophoresis showing amplification of a specific PCR product of the expected size for each gene.** M represents 50 bp DNA ladder marker (GeneRuler™ 50 bp DNA Ladder, Thermo Scientific, USA) and “-” represents negative control. Click here for file

Additional file 6 Raw CP data used for statistical analysis in this study. Click here for file
