# Supplementary material for: Identification and validation of quantitative real-time reverse transcription PCR reference genes for gene expression analysis in teak (Tectona grandis L.f.)
Source: BMC Res Notes. 2014 Jul 22;7:464. doi: 10.1186/1756-0500-7-464 (PMC4114093; doi:10.1186/1756-0500-7-464)
Supplement: Additional file 1 — Information related to the orthologous plant sequences used in this study. [file 1756-0500-7-464-S1.docx]

Additional File 1. Information related to the orthologous plant sequences used in this study.

| **Gene** | **Species** | **Reference** | **GenBank ID** |
| --- | --- | --- | --- |
| ***rp60s*** | *Populus trichoparpa* | [1] | XM_002300027.1 |
|  | *Arabidopsis thaliana* | [2] | NM_117587.2 |
|  | *Glycine max* | [3] | XM_003531057.1 |
|  | *Pisum sativum* | [4] | U10046.1 |
|  | *Ricinus communis* | [5] | XM_002513364.1 |
|  | *Vitis vinifera* | [6] | XM_002277389.2 |
| ***Cac*** | *Vitis vinifera* | [6] | XM_002281392.1 |
|  | *Arabidopsis lyrata* | [7] | XM_002894613.1 |
|  | *Populus trichoparpa* | [1] | XM_002318903.1 |
|  | *Ricinus communis* | [5] | XM_002512492.1 |
|  | *Glycine max* | [3] | XM_003535990.1 |
| ***Act*** | *Populus trichoparpa* | [1] | XM_002308329.1 |
|  | *Arabidopsis lyrata* | [7] | XM_002882721.1 |
|  | *Arabidopsis thaliana* | [2] | NM_112046.3 |
|  | *Glycine max* | [3] | NM_001254249.1 |
|  | *Ricinus communis* | [5] | XM_002530665.1 |
|  | *Vitis vinifera* | [6] | XM_002279636.1 |
| ***His3*** | *Populus trichoparpa* | [1] | XM_002306258.1 |
|  | *Gossypium hirsutum* | [8] | AF024716.1 |
|  | *Lycopersicon esculentum* | [9] | X83422.1 |
|  | *Zea mays* | [10] | EU976723.1 |
| ***Sand*** | *Populus trichoparpa* | [1] | XM_002314230.1 |
|  | *Arabidopsis thaliana* | [2] | NM_128399.3 |
|  | *Picea sitchensis* | [11] | EF676351.1 |
|  | *Vitis vinifera* | [6] | XM_002285134.1 |
| ***Β-Tub*** | *Populus trichoparpa* | [1] | XM_002298000.1 |
|  | *Gossypium hirsutum* | [12] | AF521240.1 |
|  | *Medicago truncatula* | [13] | XM_003630465.1 |
|  | *Nicotiana tabacum* | [14] | EF051136.2 |
|  | *Ricinus communis* | [5] | XM_002509755.1 |
|  | *Theobroma cacao* | [15] | GU570572.1 |
|  | *Vitis vinifera* | [6] | XM_002273478.2 |
| ***Ubq*** | *Populus trichoparpa* | [1] | XM_002320914.1 |
|  | *Hevea brasiliensis* | [16] | EF120638.1 |
|  | *Medicago truncatula* | [13] | XM_003629847.1 |
|  | *Nicotiana tabacum* | [17] | DQ138111.1 |
|  | *Pyrus communis* | [18] | AF386524.1 |
|  | *Ricinus communis* | [5] | XM_002515167.1 |
|  | *Solanum tuberosum* | [19] | L22576.1 |
| ***Ef1α*** | *Populus trichoparpa* | [1] | EF147714.1 |
|  | *Arabidopsis thaliana* | [2] | NM_100666.3 |
|  | *Elaeis guineensis* | [20] | AY550990.1 |
|  | *Gossypium hirsutum* | [21] | DQ174254.1 |
|  | *Malus domestica* | [22] | AJ223969.1 |
|  | *Nicotiana paniculata* | [23] | AB019427.1 |
|  | *Prunus persica* | [24] | FJ267653.1 |
|  | *Vitis vinifera* | [6] | XM_002284888.1 |

**Literature Cited**

1. Tuskan GA, Difazio S, Jansson S, Bohlmann J, Grigoriev I, Hellsten U, Putnam N, et al: **The genome of black cottonwood, *Populus trichocarpa* (Torr. & Gray).** *Science* 2006, **313 (5793):1596**-604.
2. Mayer K, Schüller C, Wambutt R, Murphy G, Volckaert G, Pohl T, Düsterhöft A, Stiekema W, et al: **Sequence and analysis of chromosome 4 of the plant *Arabidopsis thaliana*.** *Nature* 1999, **402(6763):**769-77.
3. Schmutz J, Cannon SB, Schlueter J, Ma J, Mitros T, Nelson W, et al: **Genome sequence of the palaeopolyploid soybean.** *Nature* 2010, **463:**178-183.
4. Stafstrom JP, Devitt ML: **Nucleotide Sequence of Four Ribosomal Protein 127 cDNAs from Growing Axillary Buds of Pea.** *Plant Physiol.* 1995, **107:**1031-1032.
5. Chan AP, Crabtree J, Zhao Q, Lorenzi H, et al: **Draft genome sequence of the oilseed species *Ricinus communis*.** *Nature Biotechnology* 2010, **28:**951–956.
6. Jaillon O, Aury JM, Noel B, Policriti A, Clepet C, Casagrande A, Choisne N, Aubourg S, et al: **The grapevine genome sequence suggests ancestral hexaploidization in major angiosperm phyla.** *Nature* 2007, **449(7161):**463-467.
7. Hu TT, Pattyn P, Bakker EG, Cao J, Cheng J, Clark RM, Fahlgren N, Fawcett JA, Grimwood J, et al: **The *Arabidopsis lyrata* genome sequence and the basis of rapid genome size change.** *Nature Genetics* 1997, **43:**476–481.
8. Turley RB: **cDNA cones encoding histone H3 (Accession No. AF024716) and histone H2B (Accession No. AF025667) from upland cotton (*Gossypium hirsutum* L.) (PGR97-182).** *Plant Physiol.* 1997, **115:**1729-1731.
9. Hartung F, Guitton C, Muhlbach HP: **The electronic Plant Gene Register.** *Plant Physiol.* 1996, **112(3):**1397-1400.
10. Alexandrov NN, Brover VV, Freidin S, Troukhan ME, Tatarinova TV, Zhang H, Swaller TJ, Lu YP, Bouck J, Flavell RB, Feldmann KA: **Insights into corn genes derived from large-scale cDNA sequencing.** *Plant Mol.* *Biol.* 2009, **69:**179-194.
11. Ralph SG, Chun HJ, Kolosova N, Cooper D, Oddy C, Ritland CE, Kirkpatrick R, Moore R, Barber S, Holt RA, Jones SJ, Marra MA, Douglas CJ, Ritland K, Bohlmann J: **A conifer genomics resource of 200,000 spruce (*Picea spp*.) ESTs and 6,464 high-quality, sequence-finished full-length cDNAs for Sitka spruce (*Picea sitchensis*).** *BMC Genomics* 2008, **9:**484.
12. Shi Y, Zhu S, Mao X, Feng J, Qin Y, Zhang L, Cheng J, Wei L, Wang Z, Zhu Y: **Transcriptome profiling, molecular biological, and physiological studies reveal a major role for ethylene in cotton fiber cell elongation.** *Plant Cell* 2006, **18:**651-664.
13. Young ND, Debellé F, Oldroyd GE, Geurts R, Cannon SB, Udvardi MK, Benedito VA, Mayer KF, Gouzy J, Schoof H, Van de Peer Y, Proost S, Cook DR, Meyers BC, Spannagl M, et al: **The Medicago genome provides insight into the evolution of rhizobial symbioses.** *Nature* 2011, **480:**520-524.
14. Matsuoka K, Demura T, Galis I, Horiguchi T, Sasaki M, Tashiro G, Fukuda H: **A Comprehensive Gene Expression Analysis Toward the Understanding of Growth and Differentiation of Tobacco BY-2 Cells.** *Plant Cell Physiol* 2004, **45(9):**1280-1289.
15. Souza VL, de Almeida AA, de S Souza J, Mangabeira PA, de Jesus RM, Pirovani CP, Ahnert D, Baligar VC, Loguercio LL: **Altered physiology, cell structure, and gene expression of *Theobroma cacao* seedlings subjected to Cu toxicity.** *Environmental science and pollution research international* 2013, 0944-1344.
16. Yun Y, Zhi-Li Z, Kuan-Can L, Wei-Guo L, Huo-Sheng S: **Cloning and characteristics of a novel gene HbUEP from latex in *Hevea brasiliensis*.** *Chinese Journal of Agricultural Biotechnology* 2008, **5(2):**165-168.
17. Lewandowskaa M, Wawrzynskaa A, Moniuszkoa G, Łukomskaa J, Zientaraa K, Piechoa M, Hodureka P, Zhukova I, Liszewskaa F, Nikiforovac V, Sirkoa A: **A Contribution to Identiﬁcation of Novel Regulators of Plant Response to Sulfur Deﬁciency: Characteristics of a Tobacco Gene UP9C, Its Protein Product and the Effects of UP9C Silencing.** *Molecular Plant* 2010, 1–14.
18. Wu J, Zhao G, Yang Y, Le W, Khan MA, Zhang S, Gu S, Huang W: **Identification of differentially expressed genes related to coloration in red/green mutant pear (*Pyrus communis* L.).** *Tree Genetics & Genomes* 2013, **9(1):**75-83.
19. Garbarino JE, Belknap WR: **Isolation of a ubiquitin-ribosomal protein gene (ubi3) from potato and expression of its promoter in transgenic plants.** *Plant Mol. Biol.* 1994, **24:**119-127.
20. Low ET, Alias H, Boon SH, Shariff EM, Tan CY, Ooi LC, Cheah SC, Raha AR, Wan KL, Singh R: **Oil palm (*Elaeis guineensis* Jacq.) tissue culture ESTs: identifying genes associated with callogenesis and embryogenesis.** *BMC Plant Biol.* 2008, **8:**62-62.
21. Xu WL, Wang XL, Wang H, Li XB: **Molecular characterization and expression analysis of nine cotton GhEF1A genes encoding translation elongation factor 1A.** *Gene* 2007, **389:**27-35.
22. Giorno F, Guerriero G, Baric S, Mariani C: **Heat shock transcriptional factors in *Malus domestica*: identification, classification and expression analysis.** *BMC Genomics* 2012, **13:**639.
23. Hashimoto A, Komori T, Yamada S, Kubo T, Imaseki H: **cDNA cloning of translation elongation factor-1 alpha from *Nicotiana paniculata* (Accession No. AB019427).** *Plant Physiol.* 1999, **119:**363-363.
24. Dube A, Bisaillon M, Perreault JP: **Identification of proteins from *Prunus persica* that interact with peach latent mosaic viroid.** *J. Virol.* 2009, **83:**12057-12067.
