# Supplementary material for: Identification and validation of quantitative real-time reverse transcription PCR reference genes for gene expression analysis in teak (Tectona grandis L.f.)
Source: BMC Res Notes. 2014 Jul 22;7:464. doi: 10.1186/1756-0500-7-464 (PMC4114093; doi:10.1186/1756-0500-7-464)
Supplement: Additional file 2 — Clustal alignments used for designing primers to amplify orthologous sequences in teak. Red and green squares mean forward and reverse primers, respectively. [file 1756-0500-7-464-S2.docx]

Additional File 2. Clustal alignments used for designing primers to amplify orthologous sequences in teak. Red and green squares mean forward and reverse primers, respectively.

1. *rp60s* gene*.* Species and accession numbers used: *Populus trichoparpa* (XM_002300027.1), *Arabidopsis thaliana* (NM_117587.2), *Glycine max* (XM_003531057.1), *Pisum sativum* (U10046.1), *Ricinus communis* (XM_002513364.1), *Vitis vinifera* (XM_002277389.2)

10 20 30 40 50 60 70 80 90 100

....|....|....|....|....|....|....|....|....|....|....|....|....|....|....|....|....|....|....|....|

***PtRp60s*** 1 ----------------------GCACTTACGGCCGGGGGTTTTGCAAGCGGCAACAGAGACAGAGAGCGGCAAG----AGCAGCGAAAATGGTGAAGTTC

***AtRp60s*** 1 ----------------------ACTTAGGGTTCATAGCAGCCAGAGAGAGAGACAAGTGAGAGGGATCTACCAAA---CGAAGCAACAATGGTGAAGTTC

***GmRp60s*** 1 ------------GCACCAGCGGAGCAATAGCAACAGAGCCAGCGAGAGCAAAACCCTAGTTCATCCATCACCAGTC--GAGGAAGAAGATGGTGAAGTTC

***PsRp60s*** 1 --------------------------------------------GAAGCAGATTCCGAACCAGAGAG--GCGTA----GGGAGCGAAAATGGTGAAATTC

***RcRp60s*** 1 ----------------------------------------------------AGCTCTTCCAGGTCGCAGCAGTA---AGAACCAAAAATGGTGAAGTTC

***VvRp60s*** 1 ACAGCCACAAAACCTAGGGTTTCCATTTAGGAGAGAAAGCGAGCAGGTAGGCTAGGGTTTTGGGTTGTCTCTCTCTCTCAGAGCAGAAATGGTGAAGTTC

110 120 130 140 150 160 170 180 190 200

....|....|....|....|....|....|....|....|....|....|....|....|....|....|....|....|....|....|....|....|

***PtRp60s*** 75 TTGAAGACAAACAAGGCCGTCATAATCCTGCAAGGAAAATATGCAGGTCGCAAAGGAGTAATCGTCAGGTCCTTCGACGATGGTACACGTGATCGCCCGT

***AtRp60s*** 76 TTGAAGCAGAACAAGGCCGTGATCCTTCTTCAAGGACGTTACGCCGGAAAGAAAGCCGTCATCATCAAATCCTTCGACGACGGTAACCGTGATCGTCCTT

***GmRp60s*** 87 CTTAAGCCCAACAAGGCTGTCATCGTCCTGCAGGGCCGCTACGCCGGGCGCAAGGCGGTGATCGTGAGGACCTTCGACGAGGGAACCAGGGAGCGCCCCT

***PsRp60s*** 51 TTGAAACCTAATAAGGCGGTGATTCTCTTGCAAGGCCGATATGCCGGCAAGAAAGCCGTGATTGTGAAAACCTTCGACGACGGAACCCGCGACAAGCCTT

***RcRp60s*** 46 TTGAAGCCCAACAAAGCCGTGATCCTCCTGCAGGGGCGCTACGCAGGGCGCAAAGCCGTGATCGTGAGATCCTTCGACGATGGAACACGTGACCGTCCCT

***VvRp60s*** 101 CTCAAGCAAAACAAGGCCGTCGTCGTCCTCCAGGGGCGTTTCGCCGGTCGGAAGGCGGTGATTGTCCGCTCTTTCGACGACGGAACCCGCGATCGGCCGT

210 220 230 240 250 260 270 280 290 300

....|....|....|....|....|....|....|....|....|....|....|....|....|....|....|....|....|....|....|....|

***PtRp60s*** 175 ACGGACACTGTTTGGTTGCAGGGATTAAGAAGTACCCAAGCAAGGTTATCAAGAAGGACTCAGCCAAAAAGACTGCCAAGAAATCCCGGGTCAAGTGCTT

***AtRp60s*** 176 ACGGACACTGCCTCGTCGCCGGACTCAAGAAGTACCCGAGCAAAGTCATCCGCAAAGACTCAGCTAAGAAGACAGCTAAGAAATCTAGGGTTAAGTGTTT

***GmRp60s*** 187 ACGGCCACTGCCTCGTCGCCGGAATCAAGAAGTACCCCAGCAAGGTCATCAAGAAGGACTCCGCCAAGAAGACGGCCAAGAAATCTAGGGTTAAGGCGTT

***PsRp60s*** 151 ACGGACACTGTCTTGTTGCTGGAATCAAGAAGTACCCTAGCAAAGTGATCAAGAAAGACTCAGCGAAGAAGACGGCAAAGAAATCTAGGGTTAAGGCATT

***RcRp60s*** 146 ATGGGCATTGCCTTGTCGCTGGCATATCAAAGTACCCAGCAAAAGTGATCAAGAAAGACTCTGCCAAGAAGACAGCAAAGAAATCTCGTGTGAAGGCATT

***VvRp60s*** 201 ATGGGCACTGCCTTGTCGCCGGAATTGCCAAGTACCCGAAGAAGGTGATCCGGAAGGACTCTGCGAAGAAGACGGCGAAGAAGTCGAGAGTGAAGGCTTT

310 320 330 340 350 360 370 380 390 400

....|....|....|....|....|....|....|....|....|....|....|....|....|....|....|....|....|....|....|....|

***PtRp60s*** 275 CATCAAGCTAGTGAACTACCAGCACCTGATGCCCACACGTTACACGCTGGATGTGGACTTGAAAGATGTTGTGACCGCTGATTGTTTGTCAACCAA-GGA

***AtRp60s*** 276 CATCAAGCTTGTTAATTACCAGCATCTGATGCCTACTCGTTACACACTCGACGTGGATCTCAAGGAAGTGGCGACTCTTGAT-GCTCTTCAGAGTAAGGA

***GmRp60s*** 287 CGTGAAGCTCGTGAACTACCAGCACCTCATGCCCACGCGTTACACGTTCGACGTGGATCTCAAGGATGCTGTTACCCCTGAT-GTTCTCGGCACCAAGGA

***PsRp60s*** 251 CGTGAAGCTGGTGAATTACCAACATCTGATGCCTACCCGTTACACTCTGGATGTGGATCTGAAGGATGCTGTTGTTCCTGAT-GTTCTTCAATCAAAGGA

***RcRp60s*** 246 TATGAAGGTAGTTAACTACAGCCATCTGATGCCAACAAGATACACACTTGATGTTGATTTGAAGGATGTGGCGACTCCCGAT-GCTTTGGTTACTAAGGA

***VvRp60s*** 301 CATCAAGCTCGTCAACTACAACCACCTGATGCCCACTCGTTACACCCTGGACGTGGATCTCAAGGACGTAGTCACCGTCGAC-GCACTTCAGAGCAGGGA

410 420 430 440 450 460 470 480 490 500

....|....|....|....|....|....|....|....|....|....|....|....|....|....|....|....|....|....|....|....|

***PtRp60s*** 374 TAAGAAGATTACTGCTTGCAAGGAGACAAAGGCTAGGTTCGAGGAGCGGTTTAAGACAGGCAAGAACAGGTGGTTTTTTACAAAGCTGAGGTTTTGAT--

***AtRp60s*** 375 TAAGAAGGTTGCTGCTCTTAAGGAAGCTAAGGCTAAGCTTGAGGAGAGGTTCAAAACCGGTAAGAACAGATGGTTCTTTACCAAGCTCAGGTTCTGAAGA

***GmRp60s*** 386 CAAGAAGGTCACTGCTCTCAAGGAGACCAAGAAGCGGTTGGAGGAGAGGTTCAAGACCGGCAAGAATAGGTGGTTCTTTACCAAACTCAGATTCTGAT--

***PsRp60s*** 350 CAAGAAGGTGACTGCACTGAAAGAAACTAAGAAGAGCCTTGAAGAGAGGTTCAAAACAGGGAAGAACAGGTGGTTTTTCACCAAGCTTAGGTTTTGAA--

***RcRp60s*** 345 TAAGAAGGTTACTGCCGCAAAAGAGATCAAGAAAAGGCTCGAGGACAGGTTCAAGACTGGCAAAAATCGTTGGTTCTTTTCCAAGCTCAGGTTTTAAAGA

***VvRp60s*** 400 CAAGAAGGTGACGGCGGCCAAGGAGACCAAGGCCAGGTTCGAGGAGCGGTTCAAGACTGGGAAGAACAGGTGGTTCTTTACCAAGCTCAGGTTCTGAG--

510 520 530 540 550 560 570 580 590 600

....|....|....|....|....|....|....|....|....|....|....|....|....|....|....|....|....|....|....|....|

***PtRp60s*** 471 ----TTAAAAATGGTCTG--TCAGT-TGTTGAGCTTTTCCAA-----GCCTTTATTATTATGATGGATTTTAG-CAGTTTTGTTGATTTTGGATCTCTCT

***AtRp60s*** 475 AATTTTCTATTTCGTGAGGAATTCA-TTTTGAGCGTTTTGTTATCGTGTTTTTTAGTTTCTAGGGTTCATTTCCCATGGTGAAAAATGTGGATCCTGTTT

***GmRp60s*** 483 ----CTCGCAATCGTGAGGTTTTAT-TATTAGGGCTTTTGGAATGTTGCCTTTTTTTTAATATTGTATTCTTG-TTTTGCAAATCTTATCAAAACTATTA

***PsRp60s*** 447 ----TTTTCACTGTTTTG--TTTCT-AGTTATACATTTT--G-----GCTTTTG--ATTATTATCAAT----G-AATTATGGATGAACTTGTGGCT---T

***RcRp60s*** 444 -----TCGATTACGGGATTACTGC--TATCGCATGTTTTTTT------TATTTAAG----TGAAGCTTCTCTGTCTGCTTTAAACATATGGCAATTTTGT

***VvRp60s*** 497 ----TCATGTGTCTTAAGGCTTTCTTTACTATGAACTCAAATCGGACTTGTTATGTTTTAGGGATTCTGTCTTTTTGATGTGTTAATTTTAAGGATTATG

610 620 630 640 650 660 670 680 690 700

....|....|....|....|....|....|....|....|....|....|....|....|....|....|....|....|....|....|....|....|

***PtRp60s*** 559 GGGTTTTTTTATAAGCAAACTCATTGCCTGGTTTAAAAAAAAAGCAAAAAAAATCCT-------------------------------------------

***AtRp60s*** 574 TGGATTGTGGAAGATGTTTTGTTGAAGTTTGGATTA--TGGATTTGATCTTTTATTTTGATTCTCTAATGCATTCTTATTTACTCT--------------

***GmRp60s*** 578 TTCTGCTCTTAAGATTTGTCGTCATCGTCTTCTTCTTTTGTTAATTAATGCATTTGCCTTCTTTTAATTTACGGGGGAAATGAATGCGAGCATGTTTTGA

***PsRp60s*** 524 AGTTTTGATTATTATGAATTTTCACGGTAATTTTTAATAGGTCAC-------------------------------------------------------

***RcRp60s*** 528 TATGTTGACTCTCGT-CTTTATTGTAGTTTGATTTAAATGGATATGAAGGTTAGCATTATT---------------------------------------

***VvRp60s*** 594 GAGATGGAGTTTAGTTTCTGTTTTGAATTTGGTTCAA-TATTGGTTAAATCTAATATGGTTGCGATTTCCTGTGTAAAATAAA-----------------

1. *Cac* gene*.* Species and accession numbers used: *Vitis vinifera* (XM_002281392.1), *Arabidopsis lyrata* (XM_002894613.1), *Populus trichoparpa* (XM_002318903.1), *Ricinus communis* (XM_002512492.1), *Glycine max* (XM_003535990.1).

10 20 30 40 50 60 70 80 90 100

....|....|....|....|....|....|....|....|....|....|....|....|....|....|....|....|....|....|....|....|

***VvCac*** 1 --------ATGTTGCAGTGTATTTTTCTTCTTTCTGATTCTGGAGAGGTAATGCTGGAGAAACAGCTCACCGGACACCGGGTTGATCGATCCATATGTGA

***AlCac*** 1 ----AGAGATGCTTCAATGTATATTCCTCATCTCCGATTCTGGAGAAGTAATGCTAGAGAAGCAGCTTACGGGTCATCGCGTTGATCGATCCATATGTGC

***PtCac*** 1 --------ATGTTGCAGTGTATATTTATTCTTTCAGATTCCGGGCAAGTAATGCTAGAGAAACAGCTAATTGGGCATAAAGTAGATAGATCCATTTGTGC

***RcCac*** 1 --------ATGCTGCAATGTATATTTCTCCTCTCAGATTGCGGGGAGGTCATTCTAGAGAAGCAGCTAACTGGTCACCGAGTAGACAGATCCATTTGTGA

***GmCac*** 1 AGCGAAAGATGTTGCAGTGCATTTTTCTTCTGTCAGATTCCGGAGAGGTAATGCTAGAGAAACAGCTTAGTGGGCACCGCGTAGATCGCTCCATATGTGC

110 120 130 140 150 160 170 180 190 200

....|....|....|....|....|....|....|....|....|....|....|....|....|....|....|....|....|....|....|....|

***VvCac*** 93 TTGGTTCTGGGAACAGACTGTCTCCCAAGCTGATTCAACCAAGCTTCCACCAGTAATTGCTTCACCAACACATTACATTTTCCAAATTACTCGTGAGGGA

***AlCac*** 97 TTGGTTCTGGGATCAATCTATTTCTCAAGGCGATTCCTTTAAGTTACTTCCAGTGATTGCTTCACCAACACATTATCTATTTCAAATCGTTCGCGATGGC

***PtCac*** 93 TTGGTTTTGGGATCAAGTCATTTCTCAAGGTGATTCCTTTAAGCAACAATCAGTTATTGCATCACCGACGCATTACTTGTTCCAAATTGTCCGGGAGGGA

***RcCac*** 93 TTGGTTTTGGAATCAAGCCATTTCTCAAGATGACTCCTTTAAGCAACAATCGGTTATTGCTTCACCAACTCATTACCTGTTTCAAATTGTTCGTGAGGGG

***GmCac*** 101 CTGGTTCTGGGACCAAGCCATTTCTCAACCTGATTCCTTCAAGCAACAACCAGTTATTGCTTCTCCTACCCATTATCTATTCCAAGTTTTTCGCGAGGGA

210 220 230 240 250 260 270 280 290 300

....|....|....|....|....|....|....|....|....|....|....|....|....|....|....|....|....|....|....|....|

***VvCac*** 193 ATCACATTCTTAGCCTGCACCCAAGTTGAAATGCCTCCTTTAATGGGCATTGAGTTTCTTTGCAGAGTAGCAGATGTCCTGTCAGATTATCTTGGAGGGT

***AlCac*** 197 ATTACCTTATTAGCTTGTAGTCAAGTTGAAATGCCACCGTTGATGGCAATCGAGTTTCTTTGCAGAGTTGCTGATGTTTTGTCTGAGTACCTTGGTGGGT

***PtCac*** 193 ATCACTTTCTTAGCTTGCACTCAACTTGAAATGCCACCTTTGATGGGCATTGAGTTTCTTTGCAGAGTAGCTGATGTCCTCTCAGATTACCTTGAAGGGT

***RcCac*** 193 ATTACTTTTTTAGCCTGTACCCAAGTTGAAATGCCACCTTTGATGGCCATTGAGTTCCTCTGCAGAGTAGCTAATATCCTCTCGGATTACCTTGAAGGGC

***GmCac*** 201 ATCACCTTTTTGGCCTGCACTCAAGTCGAAATGCCGCCATTGATGGCCATTGAGTTCCTTTGTAGGGTAGCTGATGTTCTCAATGATTATCTTGGGGGGT

310 320 330 340 350 360 370 380 390 400

....|....|....|....|....|....|....|....|....|....|....|....|....|....|....|....|....|....|....|....|

***VvCac*** 293 TGAATGAAGACGTGATCAAGGATAACTTTGTGATTGTCTATGAGCTTCTGGATGAGATGATAGACAACGGCTTCCCTCTGACAACAGAACCTAACATTCT

***AlCac*** 297 TAAATGAAGATCTGGTTAAGGATAATTTCATCATTGTCTATGAGCTTTTGGATGAGATGATCGATAATGGTTTCCCTCTCACAACAGAACCAAGCATCCT

***PtCac*** 293 TGAATGAAGATGTGATAAAGGATAACTTTGTCATTGTGTATGAGCTTTTGGACGAGATGATAGACAATGGCTTCCCCCTGACCACAGAACCTAATATCCT

***RcCac*** 293 TGAATGAAGATTTGATAAAGGATAACTTTGTCATCGTGTATGAGCTTTTGGATGAGATGATAGACAATGGATTCCCTCTAACCACAGAACCTAACATCTT

***GmCac*** 301 TGAATGAAGACTTGATCAAAGACAACTTTATCATTGTATATGAGCTGCTGGATGAGATGATAGACAATGGCTTCCCTCTAACTACGGAACCTAATATCCT

410 420 430 440 450 460 470 480 490 500

....|....|....|....|....|....|....|....|....|....|....|....|....|....|....|....|....|....|....|....|

***VvCac*** 393 AAGGGAGATGATCGCTCTACCAAATATTGTTAGCAAAGTATTGGGTGTTGTGACTGGTAACAGTTCTAATGTAAGCAACACTCTTCCAGGCGCAACAGCA

***AlCac*** 397 GAGGGAAATGATAGCTCCACCGAATCTAGTCAGCAAAATGTTGAGTGTTGTAACGGGAAATGCTTCCAATGTTAGTGACACGCTCCCGAGTGGGGCTGGC

***PtCac*** 393 GAGGGAGATGATAGCTCCACCAAATATTGTGAGCAAAATGCTGAGTGTTGTGACTGGTAACAGTTCAAATGTGAGCGACACTCTTCCAGGTGCAACAGCA

***RcCac*** 393 GAGAGAGATGATAGCACCACCAAATATTGTTAGCAAAATGCTTAGTGTTGTGACTGGTAATAGTTCAAATGTGAGTGATACTCTTCCAAATGCAACATCA

***GmCac*** 401 GCAAGAGATGATAGCTCCACCGAATATTGTTAGCAAAGTCTTGAGTGTTGTGACTGGCAGCAGCTCCAATGTGAGTGACACCCTTCCAGGTGCTACTGCG

510 520 530 540 550 560 570 580 590 600

....|....|....|....|....|....|....|....|....|....|....|....|....|....|....|....|....|....|....|....|

***VvCac*** 493 TCTTGTGTTCCATGGAGAAGTACAGAGCCAAAGCATGCAAACAATGAGGTTTATGTTGATCTTCTTGAAGAAATGGATGCAGTCATAAATAGGGATGGGA

***AlCac*** 497 TCTTGTGTTCCATGGCGACCAACAGATCCAAAGTACTCTAGCAACGAAGTTTATGTCGACCTCGTTGAAGAAATGGATGCAATTGTAAACAGGGATGGAG

***PtCac*** 493 TCTTGTGTTCCGTGGAGAACAACAGACATAAAATATGCTAACAATGAAGTTTACGTTGATCTTGTTGAAGAAATGGATGCAATTATAAATAGGGACGGGG

***RcCac*** 493 TCTTGTGTTCCATGGAGAACAACCGACGTAAAATATGCTAACAATGAAGTTTATGTTGATCTTGTTGAAGAAATGGATGCAATTATAAACAGGGATGGAG

***GmCac*** 501 TCTCTTGTTCCCTGGAGAACGGCAGACACAAAGTATGCCAACAATGAAGTTTATGTAGATCTTGTTGAAGAAATGGATGCAACAATAAACAGGGATGGAG

610 620 630 640 650 660 670 680 690 700

....|....|....|....|....|....|....|....|....|....|....|....|....|....|....|....|....|....|....|....|

***VvCac*** 593 TACTGGTGAAATGTGAGATATATGGAGAAGTGGAAGTGAACTCCCACCTTTCTGGCCTTCCTGATTTAACACTCTCATTTGCAAACCCTTCCATTCTGAA

***AlCac*** 597 AATTGGTAAAATGCGAGATTTACGGTGAGGTCCAAATGAATTCCCAGCTCAGTGGTTTTCCAGATTTGACATTGTCGTTTGCGAATCCATCTATCCTCGA

***PtCac*** 593 TCTTGGTAAAGTGTGAGATTTATGGTGAAGTTCAAGTAAACTCCCATATCACAGGTGTTCCTGAATTGACTCTGTCATTTGCAAACCCATCTATTATGGA

***RcCac*** 593 TCTTGATGAAATGTGAAATCTATGGTGAACTTCAAGTGAACTCCCATATCACAGGTGTTCCAGATTTGACTCTTTCATTTACAAACCCATCTATACTGGA

***GmCac*** 601 TTCTGGTGAAATGTGAGATCAATGGTGAGGTTCAAGTGAATTCCCATATCACAGGTCTTCCTGATTTGACTCTTTCATTTGCAAATCCTTCAATCCTTGA

710 720 730 740 750 760 770 780 790 800

....|....|....|....|....|....|....|....|....|....|....|....|....|....|....|....|....|....|....|....|

***VvCac*** 693 TGATGTGAGATTCCATCCTTGTGTTCGGTTTCGGCCATGGGAATCAAATAACATTCTCTCATTTGTGCCTCCTGATGGACAGTTTAAGCTCATGAGTTAC

***AlCac*** 697 AGACATGAGGTTTCACCCGTGTGTCCGCTTCAGACCGTGGGAATCTCATCAAGTTCTCTCCTTTGTCCCTCCAGATGGAGAGTTCAAGCTTATGAGTTAC

***PtCac*** 693 CGATGTCAGATTTCATCCCTGTGTTCGGTTTCGACCATGGGAATCCCATCATATCCTATCATTTGTGCCTCCTGATGGACTGTTTAAGCTCATGAGTTAC

***RcCac*** 693 TGATGTGAGATTTCATCCTTGTGTTCGGTTTCGACCTTGGGAGTCCCATCAGATCCTGTCGTTTGTGCCTCCTGATGGATTGTTTAAGCTCATGAGTTAC

***GmCac*** 701 TGATGTGAGGTTCCATCCCTGTGTTAGATATCGGCCCTGGGAATCCAATCAAATTCTTTCATTCGTGCCTCCTGATGGACGATTTAAGCTTATGAGTTAC

810 820 830 840 850 860 870 880 890 900

....|....|....|....|....|....|....|....|....|....|....|....|....|....|....|....|....|....|....|....|

***VvCac*** 793 AGGGTCAAAAAGTTGAGGAGTACCCCAATATATGTAAAGCCGCAGCTAACATCAGACGCTGGGACATGTCGACTCAGTGTGTTGGTTGGCATACGAAGCG

***AlCac*** 797 AGGGTGAAGAAGCTGAAGAACACACCTGTATATGTAAAGCCACAAATAACATCAGATGCGGGTACATGTCGAATTAGCGTGCTAGTGGGAATCAGAAGCG

***PtCac*** 793 AGGGTTAAAAAGTTGAAAAGTACCCCGATATATGTAAAGCCACAGATTACATCTGATGCTGGGACATGCCGCATCAATGTGATGGTTGGAATACGAAATG

***RcCac*** 793 AGGGTTAAAAAGTTAAAAACCGTACCGATATATGTAAAGCCACAACTTACATCTGATGCTGGGACATGCCGCATCAATCTGATGGTTGGAATAAAAAATG

***GmCac*** 801 AGAGTTGGAAAATTGAAGAACACCCCAATATATGTTAAGCCACAATTCACTTCAGATGGTGGAAGATGCCGTGTTAGTGTATTGGTTGGCATAAGAAATG

910 920 930 940 950 960 970 980 990 1000

....|....|....|....|....|....|....|....|....|....|....|....|....|....|....|....|....|....|....|....|

***VvCac*** 893 ATCCTGGAAAAACAATTGACTCAGTAACCGTCCAATTCCAACTACCTCCTTGTATTCTATCAGCAAATCTGTCTTCTAATCATGGAACAGTCAGCATCCT

***AlCac*** 897 ACCCAGGAAAGACGATTGAGTCCATAACCTTGAGTTTCCAGCTTCCTCATTGTGTTTCATCTGCAGATCTCTCATCAAATCATGGAACTGTAACTATTCT

***PtCac*** 893 ACCCTGGAAAGATGGTTGACTCAATAACAGTGCAATTTCAACTGCCTTCATGTGTTTTATCAGCTGACGTGACTGCAAATCATGGAGCAGTGACCGTCTT

***RcCac*** 893 ACCCTGGGAAGATGATCGACTCAATAAATGTGCAGTTCCATTTGCCTCCTTGCATTTTGTCAGCTGATCTGACGTCAAATCATGGAGTAGTGAATGTCCT

***GmCac*** 901 ATCCTGGAAAGACAATTGATAATGTTACTGTGCAGTTTCAACTTCCTTCTTGCATCTTATCAGCTGATCTGAGTTCAAATTATGGAATAGTAAACATCCT

1010 1020 1030 1040 1050 1060 1070 1080 1090 1100

....|....|....|....|....|....|....|....|....|....|....|....|....|....|....|....|....|....|....|....|

***VvCac*** 993 TGCCAATAAGACCTGCTCTTGGTCCATTGGGCGAATTCCCAAGGATAAAGCCCCTTCACTGTCTGGAACCCTAACACTTGAGACAGGCATGGAGCGCCTT

***AlCac*** 997 CTCTAACAAGACATGTACATGGACAATCGGACGAATCCCAAAAGACAAGACTCCGTGTTTGTCAGGAACACTAACGCTGGAAACAGGTTTAGAACGGCTT

***PtCac*** 993 CACAAACAAGATGTGCAATTGGTCAATTGATCGAATACCGAAAGATAGAGCCCCTGCATTGTCTGGAACACTCATGCTTGAGACAGGATTAGAGCGCCTT

***RcCac*** 993 ATCTAATAAGATGTGTGTTTGGTCAATCGATCGAATTCCTAAAGATAAAACTCCGTCATTGTCTGGTACATTAGTGCTTGAGACGGGATTAGAGCGCCTT

***GmCac*** 1001 TGCTAACAAGATATGCTCTTGGTCCATTGGTCGGATCCCAAAGGATAAGGCCCCTTCAATGTCGGGAACATTGGTGCTGGAGACTGGATTGGAGCGTCTT

1110 1120 1130 1140 1150 1160 1170 1180 1190 1200

....|....|....|....|....|....|....|....|....|....|....|....|....|....|....|....|....|....|....|....|

***VvCac*** 1093 CATGTATTTCCCACATTCCAAGTGGGCTTCAGGATCATGGGAGTTGCTCTCTCTGGCCTGCAAATAGATACATTGGATATAAAGAATCTACCAAGTCGCC

***AlCac*** 1097 CATGTGTTTCCGACATTCAAACTCGGGTTTAAGATAATGGGTATTGCTCTTTCTGGCCTTAGAATCGAGAAACTTGATCTTCAAACTATCCCTCCTCGTT

***PtCac*** 1093 CATGTATTTCCCACATTTCGAGTGGGTTTTAGGATCCAGGGTGTTGCCCTTTCTGGCCTGCAATTAGATAAACTGGATCTCAGGGTTGTACCAAGTCGTC

***RcCac*** 1093 CATGTATTCCCCATATTTCAATTGAGTTTTAGAATTCAAGGTGTTGCCCTCTCAGGCTTGCAAATAGATAAACTGGACCTGAAGGTTGTACCTAATCGTC

***GmCac*** 1101 CATGTCTTTCCCACATTTCAAGTGGGTTTTAGGATTATGGGTGTTGCCCTCTCTGGTCTGCAAATAGATAAACTAGATCTAAAGACCGTACCTTACCGTT

1210 1220 1230 1240 1250 1260 1270 1280 1290 1300

....|....|....|....|....|....|....|....|....|....|....|....|....|....|....|....|....|....|....|....|

***VvCac*** 1193 CATACAAAGGTTTTCGAGCTCTCACACAGGCGGGTCAATACGAAGTAAGGTCATAGCTTTCAACTCTCTGTTGAAGTTAATTTTGAACCATGCTGCTTCT

***AlCac*** 1197 TGTACAAAGGGTTTCGTGCTCAGACACGCGCCGGTGAGTTTGATGTCAGATTGTAGCTTTCTGGT--ACGGTCATGCCGGTCAAAAGTC---TTGACTTT

***PtCac*** 1193 TTTATAAAGGCTTTCGAGCTCTCACAAGATCAGGACTATATGAAGTGAGGTCATAG--------------------------------------------

***RcCac*** 1193 TTTATAAAGGTTTTCGAGCTTTGACACGAGCAGGACTATATGAAGTTAGGTCATAG--------------------------------------------

***GmCac*** 1201 TTTATAAAGGTTTTCGAGCTCTTACTCGGGCAGGGGAATTTGAAGTCAGGTCATAATTTTGTATTTACCATTGA-GTGATCTAAGACTTGTAATGATTCT

1. *Act* gene*.* Species and accession numbers used: *Populus trichoparpa* (XM_002308329.1), *Arabidopsis lyrata* (XM_002882721.1), *Arabidopsis thaliana* (NM_112046.3), *Glycine max* (NM_001254249.1), *Ricinus communis* (XM_002530665.1), *Vitis vinifera* (XM_002279636.1).

10 20 30 40 50 60 70 80 90 100

....|....|....|....|....|....|....|....|....|....|....|....|....|....|....|....|....|....|....|....|

***PtACT*** 1 ----------------------------------------------------------------------------------------------------

***AlACT*** 1 --------------------------------------------------------------------------------------------TTCTCTTC

***AtACT*** 1 --------------------------------------------------------------------------------------------TTCTCTTC

***GmACT*** 1 -----------------------------------------------------------------------GGACACACAAATTCACACAAACATAAAAA

***RcACT*** 1 GTAGAATTTCATTTGATGCCAATTTGCATTTGAGTGTTTTTTCCTCTATTCCTTTTCTGTTATCCTTTTTTTTTTTTTAAAAAACAAGAAGGCGGGGACC

***VvACT*** 1 ----------------------------------------------------------------------------------------------------

110 120 130 140 150 160 170 180 190 200

....|....|....|....|....|....|....|....|....|....|....|....|....|....|....|....|....|....|....|....|

***PtACT*** 1 --GATTTACATCTTCCCCTTTTTTAAAACTAGA-GGGCGGGTACCTTGAACAGAC--GCCCACACAGAGATCGAATCCAGATT--TCTAAACGGTGAGAG

***AlACT*** 9 TTCATTCGCTCCGTTTCTCTCTCAAA------------------------CACCCTCCAGGTTTCCTCAGAGATCCCTCGAATCATTTTGAAGGATATAG

***AtACT*** 9 TTCATTCGCTCCGTTTCTCTCTCAAAAACTACACACCCGTACCACACCACCACCCTCCTCGTTTCCTCAGAGATCCCCTCTCTAACTTCTAAGGATATAG

***GmACT*** 30 AAAAAAAAAACACACACACACTCTCATACACACGTTGTCGCGCACACATTCCTTCATTCCGCAGCAACAAACAAACATCTTTT-------ACCTTAAACA

***RcACT*** 101 TTAACGCCCAGCCACCACTATCCTTACCTTACACAAACCACCAACAAAATCAAATCAAAGGAAAGAAAGAAAGAACCCCTGTTCATCGAAGACATAACGA

***VvACT*** 1 --------------------------------------------TACCGTGTTTTAGAGAGAGAGAGCGAA-ATTCACAGTTG-------GGCATTAAGG

210 220 230 240 250 260 270 280 290 300

....|....|....|....|....|....|....|....|....|....|....|....|....|....|....|....|....|....|....|....|

***PtACT*** 94 GAAATGGCAGAAAGTGAAGATATTCAGCCTCTTGTTTGCGACAATGGTACCGGAATGGTCAAGGCTGGGTTTGCCGGAGATGATGCACCAAGGGCTGTTT

***AlACT*** 85 AAAATGGCAGACGGTGAAGACATTCAGCCTCTTGTCTGTGACAATGGAACCGGAATGGTTAAGGCTGGATTTGCCGGAGATGATGCACCAAGAGCTGTAT

***AtACT*** 109 AAAATGGCAGATGGTGAAGACATTCAGCCTCTCGTCTGTGACAATGGAACCGGAATGGTTAAGGCTGGATTTGCTGGAGATGATGCACCAAGAGCTGTAT

***GmACT*** 123 ACAATGGCCGATGCCGAGGATATTCAACCCCTCGTTTGCGATAATGGAACCGGAATGGTCAAGGCTGGTTTTGCTGGAGATGATGCACCGAGGGCTGTGT

***RcACT*** 201 GAAATGGCAGATGGTGAGGATATTCAACCTCTCGTGTGTGACAATGGTACCGGAATGGTGAAGGCTGGCTTTGCTGGAGATGATGCTCCAAGGGCTGTGT

***VvACT*** 49 A--ATGGCAGAAACTGAGGATATTCAGCCTCTTGTCTGCGATAATGGAACCGGAATGGTCAAGGCTGGATTTGCTGGAGATGATGCTCCGAGGGCTGTGT

310 320 330 340 350 360 370 380 390 400

....|....|....|....|....|....|....|....|....|....|....|....|....|....|....|....|....|....|....|....|

***PtACT*** 194 TTCCTAGCATTGTGGGTCGCCCACGCCACACCGGTGTGATGGTTGGTATGGGTCAAAAGGATGCGTATGTTGGGGATGAGGCACAATCAAAGAGAGGTAT

***AlACT*** 185 TCCCTAGCATTGTTGGTCGTCCTCGTCACACTGGTGTGATGGTTGGTATGGGACAAAAAGATGCTTACGTTGGTGATGAGGCTCAGTCTAAGAGAGGTAT

***AtACT*** 209 TCCCTAGCATCGTTGGTCGTCCTCGACACACTGGTGTCATGGTTGGTATGGGACAAAAAGATGCTTACGTTGGTGATGAGGCTCAGTCTAAGAGAGGTAT

***GmACT*** 223 TCCCTAGCATTGTGGGGCGTCCACGTCACACTGGGGTGATGGTTGGGATGGGGCAGAAGGATGCGTATGTTGGGGACGAGGCTCAATCCAAGAGGGGTAT

***RcACT*** 301 TCCCTAGTATTGTGGGACGCCCTCGCCACACTGGTGTGATGGTAGGTATGGGCCAAAAAGATGCCTATGTTGGTGATGAGGCTCAATCTAAGAGAGGTAT

***VvACT*** 147 TTCCTAGCATTGTGGGTAGACCTCGACACACTGGAGTGATGGTTGGGATGGGACAGAAAGATGCCTATGTCGGGGATGAGGCACAATCCAAGAGAGGTAT

410 420 430 440 450 460 470 480 490 500

....|....|....|....|....|....|....|....|....|....|....|....|....|....|....|....|....|....|....|....|

***PtACT*** 294 TTTGACTTTGAAATACCCAATTGAGCATGGTATTGTTAGCAATTGGGATGATATGGAGAAGATTTGGCATCACACCTTCTACAATGAGCTCCGTGTGGCT

***AlACT*** 285 TTTGACATTGAAATATCCTATTGAGCATGGTATTGTTAGCAACTGGGATGACATGGAGAAGATTTGGCATCACACTTTCTACAATGAGCTCCGTGTTGCA

***AtACT*** 309 TTTGACATTGAAATATCCTATTGAGCATGGTATTGTTAGCAACTGGGATGATATGGAGAAGATTTGGCATCACACTTTCTACAATGAGCTCCGTGTTGCA

***GmACT*** 323 TTTGACTCTCAAATACCCAATTGAGCATGGAATTGTGAGCAATTGGGACGACATGGAGAAGATCTGGCATCACACTTTCTACAACGAGCTTCGTGTGGCT

***RcACT*** 401 TTTGACTTTGAAGTACCCAATTGAGCATGGAATAGTTAGCAATTGGGATGACATGGAGAAGATTTGGCATCATACCTTTTACAATGAGCTCCGTGTTGCT

***VvACT*** 247 TTTAACTCTAAAATACCCAATTGAGCATGGCATTGTTAGCAATTGGGATGATATGGAAAAAATCTGGCATCACACCTTCTACAATGAGTTGCGTGTGGCT

510 520 530 540 550 560 570 580 590 600

....|....|....|....|....|....|....|....|....|....|....|....|....|....|....|....|....|....|....|....|

***PtACT*** 394 CCTGAAGAGCATCCGGTTCTCCTTACAGAAGCTCCTCTTAACCCCAAAGCCAATCGTGAGAAAATGACCCAGATCATGTTTGAGACCTTCAACACTCCCG

***AlACT*** 385 CCTGAAGAGCACCCTGTTCTTCTCACTGAGGCTCCTCTCAACCCCAAGGCCAATCGTGAGAAGATGACACAGATTATGTTTGAAACTTTCAACACTCCTG

***AtACT*** 409 CCTGAAGAACACCCTGTTCTTCTCACTGAGGCTCCTCTCAACCCCAAGGCCAATCGTGAGAAAATGACTCAGATTATGTTTGAAACTTTCAACACTCCTG

***GmACT*** 423 CCTGAGGAACACCCTGTGCTTCTCACCGAGGCACCTCTTAATCCTAAGGCTAATCGTGAGAAAATGACTCAGATCATGTTTGAGACCTTCAACACCCCTG

***RcACT*** 501 CCTGAGGAGCATCCTGTTCTTCTCACTGAAGCTCCTCTCAATCCTAAGGCCAATCGTGAGAAGATGACACAGATCATGTTTGAGACCTTTAATACTCCTG

***VvACT*** 347 CCAGAGGAGCATCCAGTGCTTCTCACTGAAGCTCCTCTCAACCCAAAGGCCAATCGTGAGAAAATGACTCAAATTATGTTCGAGACCTTCAACACACCCG

610 620 630 640 650 660 670 680 690 700

....|....|....|....|....|....|....|....|....|....|....|....|....|....|....|....|....|....|....|....|

***PtACT*** 494 CAATGTACGTTGCTATCCAGGCTGTCCTTTCCCTTTATGCCAGTGGTCGTACAACTGGTATTGTTCTGGACTCTGGAGATGGTGTGAGTCACACAGTTCC

***AlACT*** 485 CTATGTATGTCGCTATCCAAGCTGTTCTTTCCCTCTACGCCAGTGGTCGTACTACTGGTATTGTGTTGGACTCTGGAGATGGTGTGAGTCACACTGTTCC

***AtACT*** 509 CCATGTATGTCGCTATCCAAGCTGTTCTTTCCCTCTACGCTAGTGGTCGTACTACTGGTATTGTGTTGGACTCTGGAGATGGTGTGAGTCACACTGTTCC

***GmACT*** 523 CTATGTATGTCGCTATCCAGGCCGTGCTTTCCCTTTATGCTAGTGGCCGTACAACTGGTATTGTTCTGGACTCTGGAGATGGTGTCAGTCACACGGTTCC

***RcACT*** 601 CTATGTACGTTGCCATCCAGGCCGTCCTTTCCCTTTATGCCAGCGGCCGTACTACTGGTATTGTTCTGGACTCTGGAGATGGTGTGAGCCACACAGTTCC

***VvACT*** 447 CCATGTATGTCGCTATCCAGGCTGTCCTTTCCCTTTATGCCAGCGGTCGCACAACTGGTATCGTTCTGGACTCTGGTGATGGTGTGAGCCACACAGTCCC

710 720 730 740 750 760 770 780 790 800

....|....|....|....|....|....|....|....|....|....|....|....|....|....|....|....|....|....|....|....|

***PtACT*** 594 CATCTATGAAGGCTATGCCCTCCCACATGCCATTCTGCGTCTTGACCTGGCAGGCCGTGATCTCACTGATGCCCTAATGAAAATCTTGACTGAGCGTGGC

***AlACT*** 585 AATTTATGAAGGATATGCTCTTCCACACGCCATTCTGCGTTTGGACCTTGCAGGACGTGACCTTACTGATTACCTCATGAAGATCTTAACAGAGCGTGGT

***AtACT*** 609 AATTTATGAAGGATATGCTCTTCCACATGCTATTCTGCGTTTGGACCTTGCAGGCCGTGACCTTACTGATTACCTCATGAAGATCTTAACCGAGCGTGGT

***GmACT*** 623 TATCTACGAAGGTTATGCCCTCCCACATGCAATCCTGCGTTTGGACCTTGCAGGGCGTGATCTCACTGATGCCCTCATGAAAATCTTGACTGAGCGTGGT

***RcACT*** 701 CATCTATGAAGGCTATGCTCTCCCGCATGCCATTCTGCGACTTGACCTGGCAGGCCGTGATCTCACTGATGCTCTTATGAAAATTTTGACTGAGCGTGGT

***VvACT*** 547 CATTTATGAAGGGTATGCCCTCCCACATGCCATCCTGCGTCTTGACTTGGCAGGACGTGATCTCACAGATGCCCTCATGAAAATCTTGACCGAGCGTGGT

810 820 830 840 850 860 870 880 890 900

....|....|....|....|....|....|....|....|....|....|....|....|....|....|....|....|....|....|....|....|

***PtACT*** 694 TACTCTTTCACAACCACAGCAGAGCGTGAAATTGTAAGGGACATGAAGGAAAAACTAGCCTACATTGCTCTTGATTATGAGCAAGAGCTAGAGACAGCAA

***AlACT*** 685 TACTCATTCACTACCTCAGCAGAGCGTGAAATTGTAAGGGATGTGAAAGAGAAACTTTCTTACATAGCTCTTGACTACGAGCAAGAGATGGACACGGCAA

***AtACT*** 709 TACTCATTCACTACCTCAGCAGAGCGTGAAATCGTAAGGGATGTGAAAGAGAAACTTGCTTACATAGCACTTGACTACGAGCAAGAGATGGAAACAGCAA

***GmACT*** 723 TACACTTTCACCACATCTGCGGAACGGGAAATTGTGAGGGACATGAAGGAGAAACTGGCCTACATTGCTCTGGATTATGAGCAGGAGTTGGAAACTGCCA

***RcACT*** 801 TACTCTTTCACCACCACTGCAGAGAGGGAAATTGTAAGAGACATGAAGGAGAAACTATCCTACATTGCTCTTGATTATGAACAGGAGCTAGAGACGGCAA

***VvACT*** 647 TACTCGTTCACCACTACTGCAGAGCGGGAAATTGTTAGAGACATGAAAGAGAAGCTAGCCTACATCGCGCTTGACTATGAACAAGAGCTAGAGACAGCAA

910 920 930 940 950 960 970 980 990 1000

....|....|....|....|....|....|....|....|....|....|....|....|....|....|....|....|....|....|....|....|

***PtACT*** 794 AGACCAGCTCATCTGTTGAGAAGAGCTATGAGCTGCCAGATGGGCAGGTTATCACCATTGGAGCTGAACGCTTCCGTTGTCCAGAGGTCCTCTTCCAACC

***AlACT*** 785 ACACAAGCTCATCCGTTGAGAAGAGTTACGAGTTGCCTGATGGACAGGTGATCACCATTGGAGGAGAGAGATTCCGCTGCCCCGAGGTTCTGTTCCAACC

***AtACT*** 809 ACACAAGCTCATCCGTTGAGAAGAGCTATGAGTTGCCTGATGGACAGGTGATCACCATTGGAGGAGAGAGATTCCGCTGCCCGGAGGTTCTGTTCCAACC

***GmACT*** 823 AGACCAGTTCAGCTGTTGAAAAGAGCTATGAGCTACCTGATGGGCAGGTGATCACGATTGGCGCTGAACGATTCCGATGCCCTGAAGTTCTGTTCCAGCC

***RcACT*** 901 AGACCAGCTCATCTGTCGAGAAGAGTTATGAGCTGCCAGATGGGCAGGTTATCACCATTGGCGCTGAGCGATTCCGTTGTCCAGAGGTCCTCTTCCAACC

***VvACT*** 747 AGACCAGCTCATCTGTGGAGAAGAGTTATGAGCTGCCAGATGGGCAGGTGATCACCATTGGGGCTGAGCGATTCCGCTGCCCAGAGGTGCTGTTCCAGCC

1010 1020 1030 1040 1050 1060 1070 1080 1090 1100

....|....|....|....|....|....|....|....|....|....|....|....|....|....|....|....|....|....|....|....|

***PtACT*** 894 ATCCATGATAGGAATGGAAGCTGCTGGCATACATGAAACAACATACAATTCTATCATGAAGTGTGATGTCGATATTAGGAAGGATCTCTATGGCAATATT

***AlACT*** 885 ATCTTTGGTGGGAATGGAAGCTGCTGGTATTCATGAGACCACTTACAATTCCATCATGAAGTGTGATGTGGATATCAGGAAGGATCTGTATGGAAACATT

***AtACT*** 909 GTCTTTGGTGGGAATGGAAGCTGCTGGTATTCACGAGACCACTTACAATTCCATCATGAAGTGTGATGTGGATATCAGGAAGGATCTGTATGGAAACATT

***GmACT*** 923 ATCCATGATTGGGATGGAATCTCCTGGTATCCATGAGACAACATATAACTCTATCATGAAGTGTGATGTCGACATTAGGAAGGATCTCTATGGTAACATT

***RcACT*** 1001 ATCTATGATTGGGATGGAAGCTGCTGGCATACACGAAACCACTTACAACTCAATCATGAAGTGTGATGTCGATATTAGGAAGGATCTCTACGGCAATATT

***VvACT*** 847 ATCCATGATCGGGATGGAAGCTGCTGGCATTCATGAAACCACATACAACTCCATCATGAAATGTGATGTGGATATTAGGAAGGATCTGTATGGAAACATT

1110 1120 1130 1140 1150 1160 1170 1180 1190 1200

....|....|....|....|....|....|....|....|....|....|....|....|....|....|....|....|....|....|....|....|

***PtACT*** 994 GTCCTCAGTGGTGGTTCTACCATGTTCCCAGGCATTGCGGATAGGATGAGCAAGGAAATTACAGCATTAGCTCCCAGTAGCATGAAGATTAAGGTGGTTG

***AlACT*** 985 GTGCTCAGTGGTGGAACCACCATGTTCCCTGGAATTGCTGATAGGATGAGCAAAGAGATCACTGCTTTGGCTCCAAGCAGCATGAAGATTAAGGTGGTTG

***AtACT*** 1009 GTGCTCAGTGGTGGAACCACCATGTTCCCTGGAATTGCTGATAGGATGAGCAAAGAGATCACTGCTTTGGCTCCAAGCAGCATGAAGATTAAGGTGGTTG

***GmACT*** 1023 GTCTTGAGTGGTGGTTCCACAATGTTCCCTGGCATTGCTGATAGGATGAGCAAGGAGATTACAGCATTGGCACCAAGTAGCATGAAAATTAAGGTTGTAG

***RcACT*** 1101 GTCCTCAGTGGTGGCTCTACTATGTTCCCAGGCATTGCTGATAGGATGAGCAAGGAGATCACAGCATTAGCCCCCAGCAGCATGAAGATTAAGGTGGTGG

***VvACT*** 947 GTCCTCAGTGGTGGCTCCACCATGTTTCCGGGTATTGCTGACAGGATGAGCAAGGAGATTACAGCATTAGCTCCAAGCAGCATGAAAATTAAGGTGGTGG

1210 1220 1230 1240 1250 1260 1270 1280 1290 1300

....|....|....|....|....|....|....|....|....|....|....|....|....|....|....|....|....|....|....|....|

***PtACT*** 1094 CACCACCTGAAAGGAAGTACAGTGTCTGGATAGGAGGTTCCATTTTGGCATCCCTCAGCACCTTCCAGCAGATGTGGATTGCAAAGGCTGAGTATGATGA

***AlACT*** 1085 CTCCACCAGAGAGGAAGTACAGTGTCTGGATTGGAGGCTCCATTCTAGCATCACTCAGTACCTTCCAACAGATGTGGATAGCAAAGGCTGAGTATGATGA

***AtACT*** 1109 CTCCACCAGAGAGGAAGTACAGTGTCTGGATTGGAGGCTCCATTCTAGCATCACTCAGTACCTTCCAACAGATGTGGATAGCAAAGGCCGAGTATGATGA

***GmACT*** 1123 CACCACCAGAGAGGAAGTACAGTGTCTGGATTGGAGGCTCCATCTTGGCTTCCCTCAGCACCTTCCAACAGATGTGGATTGCGAAGGCAGAGTATGATGA

***RcACT*** 1201 CACCACCAGAAAGGAAGTACAGTGTCTGGATTGGAGGTTCCATTTTGGCATCCCTCAGCACCTTCCAGCAGATGTGGATTGCAAAGGCAGAATATGATGA

***VvACT*** 1047 CACCCCCGGAGAGGAAGTACAGTGTCTGGATCGGAGGCTCCATTCTGGCTTCCCTCAGCACTTTCCAGCAGATGTGGATTTCAAAGGGAGAGTATGATGA

1310 1320 1330 1340 1350 1360 1370 1380 1390 1400

....|....|....|....|....|....|....|....|....|....|....|....|....|....|....|....|....|....|....|....|

***PtACT*** 1194 GTCTGGGCCATCAATTGTCCACAGGAAGTGCTTTTAATCATGCAAAGCAAGAGGCTCTTGGACAAATACATACTATATACA-------------------

***AlACT*** 1185 GTCAGGGCCATCAATAGTCCACAGGAAGTGCTTCTAAGATT---AAGCTCGAATCAAAGTGATGAATGATTGTTCTGTATTGGTAAAG------------

***AtACT*** 1209 GTCAGGGCCATCAATAGTCCACAGGAAGTGCTTCTAAGATT---AAGCTCAAATCAAAGTGATGAATGATTGTTCTGTATTGGTAAAG------------

***GmACT*** 1223 ATCTGGACCATCAATCGTACACAGGAAATGCTTCTAA-GTTATAATAGGGAGTGTGAAAGCTGGACCAGGGAAATTACTAT-------------------

***RcACT*** 1301 GTCTGGACCATCAATTGTCCACAGGAAGTGCTTCTAATAATGCAAAGCATAAAGCTAAAGCCGGAACGCATCCTATACAAAATTCTTTTTTTCTTTCTTC

***VvACT*** 1147 GTCAGGGCCATCAATTGTTCACAGGAAATGTTTCTAA---------------------------------------------------------------

1410 1420 1430 1440 1450 1460 1470 1480 1490 1500

....|....|....|....|....|....|....|....|....|....|....|....|....|....|....|....|....|....|....|....|

***PtACT*** 1274 -CTTTGTATT-ACAGAAGGCTTCTAATCAAGTGGTTGCAAAGTTATCTTCTGCATTTCGTCACCTCTTTCAATCTCTCTCTTTTTTTTCGTGTTTTGCTC

***AlACT*** 1269 -CCTTTTGTT--CATCGACTTTGTTGCAAAATATTCTT----TTGTTTTCTATGTTTCTTCACCAC----------------------------------

***AtACT*** 1293 -CCTTTTGTT--CATCGACTTTGTTGCAAAATATTCTT----TTGTTTTCTATGTTTCTTCACCACTACATTACATTTCTTTCTTGT---TGTTATCCTC

***GmACT*** 1302 -TTATACAAATACTACAAAAATACCATCTAGTGGTTGAGGAACTTTCATTTCCTACTCTTTACCATCCTTTTATCTATCTTGTTTTTGTGTTTTCCTTTC

***RcACT*** 1401 TCTTTTTGTTTGCAGAAGGCTTCTAATCAAGTGGTTGCAAAA--ATTTTCTCTGTTTTTACTCTTTATATTTTGTTATTTTTCCATCTTCTGTTTTGCTC

***VvACT*** 1183 ----------------------------------------------------------------------------------------------------

1510 1520 1530 1540 1550 1560 1570 1580 1590 1600

....|....|....|....|....|....|....|....|....|....|....|....|....|....|....|....|....|....|....|....|

***PtACT*** 1373 CGTCAATGTCTGGACAACAAAAATGAGGTTGAGTGGATCAAATTTAAGATTCGATTATTTAATTTGTTATTGGATTTGTAGAAGAGTTGTGTAAT--GTA

***AlACT*** 1328 ----------------------------------------------------------------------------------------------------

***AtACT*** 1384 TTTTGGTGTTTCTGCTATTAATCGAAAAAGAAATTTTCTTTTCTTAGTTTC-------------------------------------------------

***GmACT*** 1402 TTTGGTATGTTGAGATAAGAGCATGAAGGCTAGCAA--GATATGTAAGATTCTTTTTTTTTCTCCCGTTCTG---TTGTAGAAGAGATGTGAATT--GTT

***RcACT*** 1499 TGTTAATGTCTGGACACCAAAGATGAGGGCGAGTGATAAATTTTCAATATTCAATTTTTTCATTTAT--TTGG--TTGTAGAAGCATTGTGTATTTTGTA

***VvACT*** 1183 ----------------------------------------------------------------------------------------------------

1. *His3* gene*.* Species and accession numbers used: *Populus trichoparpa* (XM_002306258.1), *Gossypium hirsutum* (AF024716.1), *Lycopersicon esculentum* (X83422.1), *Zea mays* (EU976723.1).

10 20 30 40 50 60 70 80 90 100

....|....|....|....|....|....|....|....|....|....|....|....|....|....|....|....|....|....|....|....|

***PtHis3*** 1 GAACGCCAAATCTCTATAAGTAAGCCTTGACTTCTCTAGATTTCGAACTTTACAAAAATTCGACGCGGAGGGAGCGAGAGATTCTTGAAGAGATTCAAAG

***GhHis3*** 1 ------------------------------------------------------------------------------------------------GAAG

***LeHis3*** 1 ---------------------------------------------------------------GGAGAAGAAGAAGAAGGAGTAATAGTTTTCCTAAGAG

***ZmHis3*** 1 ----------------------TTTTTTTCCGGCCTCCCATTCCTCGCGCGGCGACCACCCGATTCGAAGCGTGCG-GAGAGACCGAAGAAGCGGGAGAG

110 120 130 140 150 160 170 180 190 200

....|....|....|....|....|....|....|....|....|....|....|....|....|....|....|....|....|....|....|....|

***PtHis3*** 101 ATGGCCCGTACCAAGCAAACTGCTCGTAAGTCTACTGGAGGAAAGGCACCGAGGAAGCAGCTCGCTACCAAGGCTGCTCGTAAGTCTGCCCCAACCACTG

***GhHis3*** 5 ATGGCCCGTACCAAGCAAACCGCCCGTAAGTCTACTGGTGGGAAGGCTCCAAGGAAGCAACTTGCTACCAAGGCTGCCCGTAAATCTGCCCCAACCACCG

***LeHis3*** 38 ATGGCTCGTACCAAGCAAACTGCTCGTAAGTCTACAGGAGGAAAGGCTCCCAGGAAACAACTTGCCACTAAGGCTGCACGTAAGTCTGCTCCTACCACTG

***ZmHis3*** 78 ATGGCTCGTACCAAGCAGACTGCTCGCAAGTCCACGGGAGGGAAGGCTCCCAGGAAGCAGCTTGCCACCAAGGCTGCCCGTAAGTCTGCCCCCACCACTG

210 220 230 240 250 260 270 280 290 300

....|....|....|....|....|....|....|....|....|....|....|....|....|....|....|....|....|....|....|....|

***PtHis3*** 201 GTGGAGTGAAGAAGCCTCACCGTTACCGCCCTGGAACTGTTGCTCTTCGTGAAATCCGTAAGTATCAGAAGAGTACTGAGCTCCTGATCAGGAAACTCCC

***GhHis3*** 105 GTGGTGTGAAGAAGCCTCATCGATACCGTCCTGGAACTGTTGCTCTTCGTGAAATTCGTAAATACCAGAAGAGTACTGAGCTTCTTATCAGGAAATTGCC

***LeHis3*** 138 GTGGTGTGAAGAAGCCACACAGATACCGACCTGGTACTGTTGCTCTTCGTGAAATCCGTAAGTACCAAAAGAGTACTGAGCTCTTGATCAGGAAGCTTCC

***ZmHis3*** 178 GTGGAGTGAAGAAGCCTCACCGCTACCGCCCTGGAACTGTTGCACTCCGTGAGATCCGCAAGTACCAGAAGAACACTGAGCTGCTGATCAGGAAGCTGCC

310 320 330 340 350 360 370 380 390 400

....|....|....|....|....|....|....|....|....|....|....|....|....|....|....|....|....|....|....|....|

***PtHis3*** 301 TTTCCAGAGGCTTGTTCGTGAAATTGCCCAGGATTTTAAGACTGATCTGCGTTTCCAAAGCCATGCTGTCTTGGCACTGCAAGAGGCAGCTGAGGCATAC

***GhHis3*** 205 TTTCCAGAGGCTTGTTCGTGAAATTGCCCAGGACTTCAAGACTGATTTGCGTTTCCAGAGCCATGCTGTTCTAGCTCTCCAGGAAGCTGCAGAGGCATAC

***LeHis3*** 238 ATTCCAGAGGCTTGTTCGTGAAATTGCCCAGGACTTCAAGACTGATTTGCGTTTCCAGAGTCATGCGGTGCTAGCTCTGCAAGAGGCTGCTGAGGCCTAC

***ZmHis3*** 278 CTTCCAGAGGCTCGTTAGGGAAATTGCACAGGACTTCAAGACTGATTTGCGTTTCCAGAGCCATGCGGTGCTTGCTCTCCAGGAGGCTGCTGAGGCATAC

410 420 430 440 450 460 470 480 490 500

....|....|....|....|....|....|....|....|....|....|....|....|....|....|....|....|....|....|....|....|

***PtHis3*** 401 CTTGTTGGGCTGTTCGAAGACACCAACCTTTGTGCCATCCATGCCAAACGTGTCACCATCATGCCCAAGGATATCCAGCTGGCTAGGAGGATCAGGGGTG

***GhHis3*** 305 CTTGTGGGTCTTTTTGAAGACACCAACCTTTGCGCGATCCATGCCAAGCGTGTCACAATTATGCCCAAGGACATCCAGTTGGCTCGTAGGATCAGGGGAG

***LeHis3*** 338 TTGGTGGGTCTCTTTGAGGACACTAACCTTTGTGCCATTCACGCCAAGCGTGTGACTATCATGCCAAAGGACATTCAGCTTGCCAGGCGAATTAGAGGCG

***ZmHis3*** 378 CTTGTTGGCCTGTTTGAGGACACCAACCTGTGCGCCATCCATGCTAAGCGTGTGACCATCATGCCCAAGGACATTCAGCTGGCAAGGAGGATCCGCGGCG

510 520 530 540 550 560 570 580 590 600

....|....|....|....|....|....|....|....|....|....|....|....|....|....|....|....|....|....|....|....|

***PtHis3*** 501 AGCGTGCTTAAGGGGGCACT---------TGCTGAAAGGGGAGAACACTCTTCTTTGTATCGGCAGCAGCAAGGTTTAGGTAGT--TGATAGATAGGTTT

***GhHis3*** 405 AGCGTGCTTAA--------T---------TTCCAGAAGTGTGGATCAGTCGCTTAGAAGAAACTATTGTTTATTTTTAGGCA-----AATGTATTGGTTT

***LeHis3*** 438 AGCGTGCTTAGTT---TGTT---------TACTGAAGTAGTAGCTTTGTTTGTCGTATTTTAAC-TCTTTTCTTGTTAGACAAAGACAACTAATGAATTT

***ZmHis3*** 478 AGAGGGCCTAATCGCCACCTCAAACATCGTGACAAAAAAATGAAGTCCTGGGGTTATTGTTAATCTGGTGCCATTGTAAGGACA---TATGAGTAGGGTT

610 620 630 640 650 660 670 680 690 700

....|....|....|....|....|....|....|....|....|....|....|....|....|....|....|....|....|....|....|....|

***PtHis3*** 590 GGTGGTAGTGGTGTACGGCTGTGTGATGGTTGGGTTTGTAGAAGGCTGTGTGATGGTGTGGGGGGGTTAGTAGAAGGTGTTTTATCTTG-TGGGGATTGA

***GhHis3*** 483 TTTGTTTTTTCTAATCATCTTTTAAGCCATGATGGTAGTGGTAGGTTCAATG-TGATGTGATGATGA-AGTGGGGCGTCTGATATGTTAATGGACAATAA

***LeHis3*** 525 AGTAGTAG-GGTAATGTGCTTCTAAGT--TTGTTTTGGTAGCCCGGAATGTGCTGTAGTCTTGTTGC-CGTTGTAGACATTTTGTCT------AGATTGT

***ZmHis3*** 575 TGTTTTGTGGATCGCAAGTTTCTCTTCTGCTACTGTTGCTGCTACCTT--TGCTGGTGT--TATTGCTAGGGTTGAATACTTAAGTTAACTATGCGCTGA

710 720 730 740 750 760 770 780 790 800

....|....|....|....|....|....|....|....|....|....|....|....|....|....|....|....|....|....|....|....|

***PtHis3*** 689 ACC---TTTGATATA-TTTTATTCCGTTGTAACTCTG---TTTGACCGATTGTC-TTTTCTTTCGTGACATGAGGCCACAAGGAAGTGGCTGCATGAACA

***GhHis3*** 581 CTCATGTCTGGTATAGTCTTGTTTAAATGTGTTTTTG---CTTAATCGAAAGTTGCTTTGCCACACAGTA-GTGGCTTTGGTTGTTTAAAAAAAAAAAAA

***LeHis3*** 615 CACACCTCTGGTGTT-TAACAATGTTCAGTATTTTCA---TCTAATGGCTAATCTCCA------------------------------------------

***ZmHis3*** 671 CAG---TCTGGACTAGCAGTGTTATG-TGCGCTGCTGGACCTTGCGCGCGATGCTGTGGCCGATTTTGTTCGTTAATGTCAAGAATTGTGTTAAAAAAAA

810 820 830 840

....|....|....|....|....|....|....|....|

***PtHis3*** 781 AATGCCTATATTTTTGATCTAATTCGTAGTTTAGTTTTGC

***GhHis3*** 677 AAAAAAAAAA------------------------------

***LeHis3*** 668 ----------------------------------------

***ZmHis3*** 767 AAAAAAAAAA------------------------------

1. *Sand* gene*.* Species and accession numbers used: *Populus trichoparpa* (XM_002314230.1), *Arabidopsis thaliana* (NM_128399.3), *Picea sitchensis* (EF676351.1)*, Vitis vinifera* (XM_002285134.1).

10 20 30 40 50 60 70 80 90 100

....|....|....|....|....|....|....|....|....|....|....|....|....|....|....|....|....|....|....|....|

***PtSand*** 1 ----------------------------------------------------------------------------------------------------

***AtSand*** 1 ----------------------------------------------------------------------------------------------------

***PsSand*** 1 TGATTCTCTTCATTAAATTCAACGTTTGATTCCTCTTCCAGTCACTTAATTTCATATATTTTTCAATCTCGGGAATTTGATGGTGTGAAGTTTCCTTAGC

***VvSand*** 1 ----------------------------------------------------------------------------------------------------

110 120 130 140 150 160 170 180 190 200

....|....|....|....|....|....|....|....|....|....|....|....|....|....|....|....|....|....|....|....|

***PtSand*** 1 -------------ACATCGTCATAATAAGCTAAGAGGACGGAGTAGACCCGACACATGTAAAGTCGGATCCAAGTAATCCACCTACTGGTCAGAGCCTCG

***AtSand*** 1 ----------------------------------------------------------------------------------------------------

***PsSand*** 101 TTTGAAATTCGCAGGATCGCTTCATATATGTTTTCTGAAGACGTACAGGGCTTCGGTGTTCAGAAGAATTCGATATTTTGTATTTGAAATTTCTGTATAT

***VvSand*** 1 ----------------------------------------------------------------------------------------------------

210 220 230 240 250 260 270 280 290 300

....|....|....|....|....|....|....|....|....|....|....|....|....|....|....|....|....|....|....|....|

***PtSand*** 88 TCTCTCGTGTCTCTCTCCCTCTTTGGCAACGACGCACCTAATTGCAGAG-ACCTCGAATCCAACAAAAAACCGACTGGTAGAGA--GACCTAACAGCCAA

***AtSand*** 1 ------------------------------------------AGCAGAG-AGAATCAATGAACACGTCTTGCCATTAGAGGAGACCAACCATTTCTCTCC

***PsSand*** 201 ATACAACAATGGAAACAGATTCGGCTCGGGAAAGCGGCGAGGAAGAGAATGGTTTTACTCAGCCTGAGATAGGAGATGACGATATTGATAATGTCGCAGA

***VvSand*** 1 ---------------------------------------------------------------------------------------------GCTCCAA

310 320 330 340 350 360 370 380 390 400

....|....|....|....|....|....|....|....|....|....|....|....|....|....|....|....|....|....|....|....|

***PtSand*** 185 CGCATCCGAAAA--GAT----ATAACAGAGACCCAAAAAGATAAAAATAAA---AAAACCCTCTCTCTTCCTCTTCCTCTCTCGTAATTACGAAATTCCA

***AtSand*** 58 TCTCTCTTGAAA--AATCT--ATAACCGATTTCTGCAATGGCGACTTCAGATTCGAGGTCTTCTC-CTTCATCATCCGACACCGAATTCGC-CGATCCAA

***PsSand*** 301 AGATTTCAGAGATGGATTAGGGTTATTGAGTCCGAAAGAGAAAACCATGGAGGAGAGAAGGAATGGAACCCTTGATTCCGACAATGATAGCGATGACAAT

***VvSand*** 8 CGAATTCCGAAA--TATC---GCCCCCCATATCTTACCCAATGTCGTCCGATTCGAGCTCCTCCATATCCAATGACGGCTCCACTGACCA--AAACCCTA

410 420 430 440 450 460 470 480 490 500

....|....|....|....|....|....|....|....|....|....|....|....|....|....|....|....|....|....|....|....|

***PtSand*** 276 AATATGTCCTCATCCG-ATTCCAACTCCTC--CTCCGTTGACGATCCCAACCCTAACCCCAAGCCTTTGGATTA--CCAATTCGAAACCCTAAACCTTGA

***AtSand*** 152 ATCCTAGCTCCGATCC-AGAGACGAATTCGGAGCGTGTTCAAAGTCAA----TTAGAGTCAATGAATTTATCTCAACCTAGCGAAGTCTCTGA-------

***PsSand*** 401 AGCAGGATTCGAACCGTTGACGAGAATGGGAATGCCGATGATAGCTCAAGGATCGAGGTTGAGGAAGGGCAGTCTGATAGCTCAAGGATTGAAGTTG---

***VvSand*** 101 ACCCTAGCCCCAC----AGCCAAACCCCTCGACTCCCTTCAAGATCGC----TTGGCCTCGATCGCGTTGACTGAGCCCAACGGCGGCGCCGAATCGCCA

510 520 530 540 550 560 570 580 590 600

....|....|....|....|....|....|....|....|....|....|....|....|....|....|....|....|....|....|....|....|

***PtSand*** 371 GCAAGGATCCGGTAGCACCATCATCCAAAACGACGTCGACGAAGAAGGACGACAACAAGATCAAGGCTCCTCTTTAAATGGATCGCT-GAATGTAAACAG

***AtSand*** 239 ---------TGGTAGCCACACCGAATTTAGCGGTGGCGGCGATGA-TAATGATGATGAGGTTGCATCGGCTAACGGGAACGAAGGCGGAGTTAGCAATGG

***PsSand*** 497 ----AGGAAGGGCAGTCTGAT-AGCTCAAGAATTGAGGTTGAGGA------AGGACAGGCTGATAGCAATCGACAAATTGCAGAATTTAGCTCGGAAAAT

***VvSand*** 193 TC--GGATCAGG-AGCCTCA--AGCCGGG--GTCGCAAACGGATC-GTTCAGTGAGGAGATCCAGGAAGTGGTTCAGAATAATCA---AGCTGCTGGCAG

610 620 630 640 650 660 670 680 690 700

....|....|....|....|....|....|....|....|....|....|....|....|....|....|....|....|....|....|....|....|

***PtSand*** 470 TAA--CAATAACGAACAAGATGACAGAATTGGCCTCGTCAGGAGTGTCGTGTTGAGGCGGACGAACTCGGAGGTGGAGGTGGACGTGAACGGGCCTTCCA

***AtSand*** 330 AGGTTTATTGCGTGAAGGTGTGGCGGGA--ACTAGCGGAGGAGAGGTTTTGTTAAGGGCGGAAAATCCGGTGGAAATGGAAGCAGGTGAAGAACCACCGA

***PsSand*** 587 AACAATTTTGTGGAGGGACATAAGGAAA--TTTCAGAGGCACGTGAACATTTGGAGGATGAT-TTTTCAGAACAGGAAATTGAGGTTGAAGCTCCTACTA

***VvSand*** 282 TGAGGCGGTGGTTGAAGAGGTGAGTGAG--AGCTTCACTCATGGAGTGGTGTGGAGG--GAC-AATTCGGAGCATGAAGTTGATGCG------CCTTCCA

710 720 730 740 750 760 770 780 790 800

....|....|....|....|....|....|....|....|....|....|....|....|....|....|....|....|....|....|....|....|

***PtSand*** 568 GCCCGAGCAGTAGTGGATACGCCGGCGAGAGAGGGAGCAGTGGCG------------------TCAGTG---------AGGATGATGA-G------ATAG

***AtSand*** 428 GTCCGACTAGTAGCGGTTACGATGGAGAGAGAGGAAGTAGCGGCGGAGCTA------CTTCTACTTATA---------AAGCTGATGATG------GAAG

***PsSand*** 684 GTCCCAGCAGCAGTGGATACGCAGGTGGCAGAGGCAGTAGTAGCGCTGCCAGCATTGGCAGTGCCAGTGGATCGGAGGAGATTAGGGACGCTTTGCGAAG

***VvSand*** 371 GCCCTAGCAGTAGCGGCTATGCTGGGGAACGGGGCAGTAGTAGTGCGACGAGTGAGTCTGGGATTGGGG---------AGGGTGGTGAAG------ATGA

810 820 830 840 850 860 870 880 890 900

....|....|....|....|....|....|....|....|....|....|....|....|....|....|....|....|....|....|....|....|

***PtSand*** 634 AG-----GAGGTTGCAATTGATAGTGCTCTCCATGAG---GTTTTTGATTCACAAGCTGCCTGGTTGCCTGGCAAACGTCATGTCGATGAGGATGATGCT

***AtSand*** 507 CGAGGATGAGATTAGGGAAGCTAATGTGGATGGTGACACTGCCTCGCAGCATGAAGCTGCGTGGTTGCCTGGAAAACGCCATGTTGATGAGGATGATGCT

***PsSand*** 784 CGGGGATGGAGTTACAGAATGTTTTGGCAATGGCAACGGGGAGCACCGAGGGCAAGCGAGTTGGGCACATGGCAAGCGGTATTCAAATGAGGATGAGACA

***VvSand*** 456 AATTCTCGAAGTTAGGAATGATGATTCCGTTGATGGG---GTATCGGATTTACAGCAATCGTGGGTTCCAGGGAAGCGTCACGTCGATGAGGACGATGCT

910 920 930 940 950 960 970 980 990 1000

....|....|....|....|....|....|....|....|....|....|....|....|....|....|....|....|....|....|....|....|

***PtSand*** 726 TCCATATCATGGAGGAAAAGGAAGAAGCATTTTTTTATATTGAGTCACTCTGGAAAACCAATATATTCCAGATATGGAGATGAACACAAGCTAGCAGGAT

***AtSand*** 607 TCTACGTCATGGAGAAAGAGGAAGAAGCATTTCTTCATACTGAGTAACTCAGGCAAACCGATATATTCCAGATATGGAGATGAACATAAGCTTGCTGGAT

***PsSand*** 884 TCAATTTCTTGGAGGAAGAGAAAGAAGCACTTCTTTGTACTTAGTCATTCTGGGAAGCCAATTTATTCCAGATATGGGGATGAGCATAAGCTAGCAGGAT

***VvSand*** 553 TCTATTTCATGGAGGAAAAGAAAGAAGCACTTTTTCATTCTGAGTCACTCTGGGAAACCAATATATTCCAGATATGGAGATGAGCACAAGCTCGCAGGAT

1010 1020 1030 1040 1050 1060 1070 1080 1090 1100

....|....|....|....|....|....|....|....|....|....|....|....|....|....|....|....|....|....|....|....|

***PtSand*** 826 TTTCAGCGACACTGCAGGCCATAATTTCATTTGTGGAGAATGGGGGGGATCGTGTCAAATTGGTTAGGGCAGGAAAGCACCAGGTGGTTTTTCTTGTAAA

***AtSand*** 707 TTTCAGCTACTCTTCAAGCTATTATTTCTTTTGTGGAGAATGGTGGTGACCGTGTCAACTTAGTCAAGGCAGGAAATCACCAGGTTGTCTTTCTCGTTAA

***PsSand*** 984 TTTCAGCAACCTTGCAAGCGATCGTTTCCTTTGTGGAGAATGGTGGAGACCACATAAAATTGGTGCGGGCAGGCAACCATCAGATTATTTTTCTAGTTAA

***VvSand*** 653 TCTCAGCAACATTGCAAGCTATCATTTCGTTTGTGGAGAATGGGGGAGATCGTGTCCAATTAATAAGGGCAGGAAAACACCAGGTGGTTTTTCTAGTGAA

1110 1120 1130 1140 1150 1160 1170 1180 1190 1200

....|....|....|....|....|....|....|....|....|....|....|....|....|....|....|....|....|....|....|....|

***PtSand*** 926 AGGACCAATTTACTTGGTGTGCATCAGCTGCACGGAACAGCCATATGAATCATTGAGGGGGGAATTGGAGCTTATTTATGGTCAGATGATACTCATTTTA

***AtSand*** 807 GGGGCCAATATATCTGGTCTGCATCAGCTGTACAGATGAAACATATGAGTATTTAAGGGGGCAGTTGGATCTTCTATATGGTCAGATGATACTAATTTTA

***PsSand*** 1084 GGGTCCTATCTATTTGGTCTGTATAAGCTGTACAGAAGAGCCATTTCAAGCTTTGAAAGGGCAGCTAGAGCTTCTTTATGACCAGATGTTGCTTATTCTG

***VvSand*** 753 AGGACCAATTTACTTAGTTTGCATCAGCTGTACAGAAGAGCCTTACGAGTCATTAAGAAGTCAGTTGGAGCTTATTTATGGTCAGATGCTACTTATTCTG

1210 1220 1230 1240 1250 1260 1270 1280 1290 1300

....|....|....|....|....|....|....|....|....|....|....|....|....|....|....|....|....|....|....|....|

***PtSand*** 1026 ACAAAGTCGGTTAATAGATGTTTTGAGAAAAATCCGAAGTTTGATATGACTCCATTGCTTGGAGGAACGGATGTTGTCTTCTCATCTCTCATCCATTCAT

***AtSand*** 907 ACAAAATCAATAGACAGATGTTTTGAAAAGAATGCAAAGTTCGATATGACACCCTTGCTTGGAGGGACAGATGCTGTCTTCTCATCTCTTGTCCATTCAT

***PsSand*** 1184 ACAAAGTCAATAGATAAATGCTTTGAAAAAAATTCAAAGTTTGACATGACACCCTTACTTGGAGGCACGGATGTAGTCTTCTCCTCTCTTATACATGCTT

***VvSand*** 853 ACAAAGTCAGTAAATAGATGTTTTGAGAAGAATCCAAAGTTTGATATGACCCCTTTGCTCGGAGGAACAGATGTTGTCTTCTCTTCTCTCATTCATTCTT

1310 1320 1330 1340 1350 1360 1370 1380 1390 1400

....|....|....|....|....|....|....|....|....|....|....|....|....|....|....|....|....|....|....|....|

***PtSand*** 1126 TTAGTTGGAATCCAGCAACATTTCTCCATGCATACACTTGTCTTCCCCTTGCTTATGGAACGAGGCAAGCTGCAGGTGCTATACTGCATGATGTTGCTGA

***AtSand*** 1007 TTAGCTGGAACCCAGCTACATTTCTTCATGCCTATACTTGTCTTCCCCTTCCATATGCGTTAAGGCAAGCTACAGGAACCATATTGCAAGAAGTTTGCGC

***PsSand*** 1284 TCAGTTGGAATCCAGCAACATATTTGCATGCATATACCTGCCTTCCCCTGCGACACTCCACAAGACAAGCTGCAGGAGCTATTCTCCAAGATGTAGCGGA

***VvSand*** 953 TCAATTGGAACCCAGCTACATTTCTTCATGCATACACCTGTCTTCCCCTTGCTTATGCGACAAGGCAAGCTTCAGGTGCCATATTACAAGATGTTGCTGA

1410 1420 1430 1440 1450 1460 1470 1480 1490 1500

....|....|....|....|....|....|....|....|....|....|....|....|....|....|....|....|....|....|....|....|

***PtSand*** 1226 TTCTGGTGTTCTCTTTGCAATATTAATGTGCAAACACAAAGTTGTTAGTCTTGTTGGTGCTCAAAAAGCTTCTCTTCATCCTGATGACATGCTGCTACTT

***AtSand*** 1107 GTCTGGTGTCTTATTCTCACTACTAATGTGCAGACACAAGGTTGTCAGTCTTGCTGGTGCACAGAAAGCGTCTCTCCATCCCGATGACTTGCTTCTACTC

***PsSand*** 1384 TTCTGGTGTCTTATTTGCTATTCTCATGTGCAGACATAAGGTTATCAGCCTTTTTGGGGCGCAAAAGGCAATCCTTCATCCAGATGACATGCTTTTACTT

***VvSand*** 1053 TTCAGGCGTCCTTTTTGCAATACTAATGTGTAAACACAAGGTCATTAGTCTTGTTGGTGCACAAAAAGCATCTCTTCACCCTGATGATATGCTGCTGCTT

1510 1520 1530 1540 1550 1560 1570 1580 1590 1600

....|....|....|....|....|....|....|....|....|....|....|....|....|....|....|....|....|....|....|....|

***PtSand*** 1326 TCCAACTTCATAATGTCTTCAGAATCATTTAGGCAAGTAAAATGGACTTTC---ATTAGACTTC-----TGTTATCCCATTCGACATCTGAATCTTTCTC

***AtSand*** 1207 TCAAATTTTGTCATGTCATCAGAATCATTCAGGACATCAGAATCTTTCTCACCAATCTGCCTACCAAGATACAACGCTCAGGCCTTTTTGCATGCCTATG

***PsSand*** 1484 TCAAATTTTGTTTTATCATCGGAATCTTTCAGGACATCAGAATCATTTTCTCCTATTTGTCTGCCACAATTCAATCCAATGGCATTCCTTTATGCTTATG

***VvSand*** 1153 TCAAACTTTGTTATGTCATCTGAATCATTTAGGACATCCGAATCTTTCTCACCAATTTGCCTGCCAAGATATAATCCCATGGCATTTTTATATGCTTATG

1610 1620 1630 1640 1650 1660 1670 1680 1690 1700

....|....|....|....|....|....|....|....|....|....|....|....|....|....|....|....|....|....|....|....|

***PtSand*** 1418 GCCAATTTGCCTGCCAAGATATAACCCAATGGCATTTTTGTATGCTTATGTCCGTTATCTTGATGTTGACACATACTTGATGATTCGTATCGAAATGGTT

***AtSand*** 1307 TCCACTTCTTTGATGATGATACATATG--TAATATTGCTTACCACACGTTCAGATGCGTTCCATCATCTCAAAGATTGCAGGGTACGCCTTGAGGCTGTT

***PsSand*** 1584 TGCAATACCTTGGAGTAGACACCTACT--TGATGTTGCTCACAACTGATTCTGATTCCTTCTTCCATCTGAAGGAATGCAGGATTCGTATTGAGAATGTA

***VvSand*** 1253 TCCATTATCTTGATGTTGACACATACT--TGATGTTGCTTACTACTAAATCAGATGCTTTCTATCATCTCAAAGATTGCAGGCTTCGTATTGAGACGGTG

1710 1720 1730 1740 1750 1760 1770 1780 1790 1800

....|....|....|....|....|....|....|....|....|....|....|....|....|....|....|....|....|....|....|....|

***PtSand*** 1518 CTTTTGAAGTCAAATGTTCTTAGCGAAGTTCAGAGGTCCATGCTGGATGGTGGGATGCATGTTGAGGATTTGCCTGCCGATCCATTGTCTCGTCCTGGAT

***AtSand*** 1405 CTTCTCAAGTCAAATATTCTAAGTGTGGTTCAAAGATCAATCGCGGAAGGTGGAATGCGTGTTGAAGATGTACCAATAGACCGCAGGCGTCG--------

***PsSand*** 1682 CTAGTCAAATCAAATGTCCTAAGTGAGGTTCAGAGGTCAATGCTAGATGGTTGTCTACGTGTGGAGGACCTACCTGGTGATCCAACATTGCCGTCAGATT

***VvSand*** 1351 CTTTTGAAGTCAAATGTTCTCAGCGAAGTTCAGAGATCCCTGCTAGATGGTGGGATGCGTGTGGAGGATTTGCCTGTTGATACATCTCCTCGCTCTGGTA

1810 1820 1830 1840 1850 1860 1870 1880 1890 1900

....|....|....|....|....|....|....|....|....|....|....|....|....|....|....|....|....|....|....|....|

***PtSand*** 1618 CTGCTTCGCCTCATTTTGGGGAGCATCAGGAACCGACCGATTCTC---CTAGGAGATTTAGGGAACCATTTGCTGGGATTGGTGGTCCTGCTGGACTTTG

***AtSand*** 1496 ----ATCATCTACTACTAATCAAGAACAAGACTCACCTGGTCCC--------GACAT----------ATCTGTGGGAACCGGAGGTCCCTTTGGACTTTG

***PsSand*** 1782 CTCTCTCTTTTCGTTTACGACGGGATAAGAACCTGCAGGTAGCTGGATCTTCAACAGGAACTGGAAGAAACACTGGAATTGGAGGTCCAGCTGGGCTTTG

***VvSand*** 1451 TTTTATCTGCTCATTTAGGCCAGCACAAACTTCCAACAGATTCTC---CAGAAACATCTAGGGAAGAATGTATTGGTGTTGGTGGTCCTTTTGGACTTTG

1910 1920 1930 1940 1950 1960 1970 1980 1990 2000

....|....|....|....|....|....|....|....|....|....|....|....|....|....|....|....|....|....|....|....|

***PtSand*** 1715 GCATTTCATATATCGTAGTATCTATCTGGAGCAATATATATCTTCTGAGTTTTCGGCACCAATTAATAGTCCACAACAGCAGAAAAGATTGTACAGGGCT

***AtSand*** 1575 GCATTTCATGTACCGTAGTATATACTTAGATCAATACATTTCCTCGGAATTCTCACCCCCAGTAACTAGTCACAGACAACAGAAAAGTCTATATCGAGCA

***PsSand*** 1882 GCATTTTATGTACCGTAGTAACTATCTTGATCAGTATGTGGCTTCAGAGTTTTCACCACCCATAAACAGCCGCAATGCACAGAAAAGGCTATTCAGAGCT

***VvSand*** 1548 GCATTTCATATATCGCAGCATATATCTGGATCAGTATGTATCTTCGGAGTTCTCACCACCAATTAACAGTTCCAGACAGCAGAAAAGATTATATAGAGCT

2010 2020 2030 2040 2050 2060 2070 2080 2090 2100

....|....|....|....|....|....|....|....|....|....|....|....|....|....|....|....|....|....|....|....|

***PtSand*** 1815 TACCAGAAACTTTACGCTTCGATGCATGATAAAGGCAACGGGGCGC----ACAAAACACAGTTTAGAAGAGATGAGAATTATGTTCTCCTCTGTTGGGTC

***AtSand*** 1675 TACCAGAAACTTTATGCTTCAATGCATGTAAAAGG-ATTGGGACCC---CACAAGACTCAATATAGAAGAGATGAAAACTACACTCTTCTATGTTGGGTC

***PsSand*** 1982 TATCAGAAGTTGCATACCTCAATGCATGATAAGGATGTAGGGCCTC----ATAAGATGCAGTACAGGAAGGATGAAAACTATGTTTTACTATGCTGGATT

***VvSand*** 1648 TACCAGAAGCTTTATGCTTCCATGCATGATAGAGG-AGTGGGCCCCCCCCATAAAACACAGTTTAGAAGGGATGAAAACTATGTTCTCCTCTGCTGGGTT

2110 2120 2130 2140 2150 2160 2170 2180 2190 2200

....|....|....|....|....|....|....|....|....|....|....|....|....|....|....|....|....|....|....|....|

***PtSand*** 1911 ACCCCAGATTTTGAGCTTTATGCGACATTTGATCCACTTGCAGACAAGGGTTTGGCAATAAAGACTTGCAACAGGGTCTGTCAATGGGTGAAAGATGTTG

***AtSand*** 1771 ACACCAGATTTTGAACTCTATGCAGCATTTGATCCACTTGCAGACAAGGCGATGGCGATAAAGATATGCAATCAGGTGTGCCAAAGGGTAAAAGATGTGG

***PsSand*** 2078 ACTCAGGAATTTGAGCTTTATGCAGCTTTTGATCCACTAGCTGAAAAGAGTTCAGCAATAACTGTTTGTAATCGTGTTTGCCAGTGGCTAAGGGATGTGG

***VvSand*** 1747 ACCCCGGAGTTTGAACTTTATGCAGCATTTGATCCACTTGCAGATAAGGCCTTGGCGATACGGACGTGCAACCGGGTCTGTCAATGGGTAAAGGATGTTG

2210 2220 2230 2240 2250 2260 2270 2280 2290 2300

....|....|....|....|....|....|....|....|....|....|....|....|....|....|....|....|....|....|....|....|

***PtSand*** 2011 AAAATGAGATATTTTTGCTGGGAGCAAGCCCCTTTTCATGGTGAC-----CC-CAAAAATATCT-----TGTAACACAGCATGTATACTAGGGTTT----

***AtSand*** 1871 AGAATGAAGTGTTCTTGCAAGGAGCTAGTCCTTTCTCTTGGTGATAATATTTTACTATTCACTC-----TTTAATATA-TATGTATTTTTT--TTT----

***PsSand*** 2178 AAAGTGAGATATTTCTACTGGAGGCGAGTCCACTCTCTTGGTGAATCGTATCAAGTGTACAGCTATAGTTGAAACCGAGCTTGAATTCAGATTCTTGGAA

***VvSand*** 1847 AAAATGAGATTTTCTTGTTGGGAGCAAGCCCCTTTTCATGGTGAT-----TTTCTCAAATATTT-----TGTAACACAGCATGGACTCTATAGTTA----

2310 2320 2330 2340 2350 2360 2370 2380 2390 2400

....|....|....|....|....|....|....|....|....|....|....|....|....|....|....|....|....|....|....|....|

***PtSand*** 2095 -GTTTATTCAATAAAATCCCACATTTAATTTCTGTGAATCTCACGTTTAATTTAAGCGTGCCAATAGTTTACCAAAAATCTGTTTCATTTACTTTCCAAA

***AtSand*** 1958 -AATCTTTCTACTGGTTTGACCCCCTATATTGTGCTAGTACCAC--CCACCTTAA-TAAGGCAATAATGTAATTGTACCCAGAAACCCTTGAGAGAAAGC

***PsSand*** 2278 AAAAAAAATTTAAGAGTAGAAAAATTGTTTGTTATCATTTTCATTAACAAACCAAGCTTTTTTGTATAATGAGGCATCATCACTGTGAGTTGATGCCAGA

***VvSand*** 1932 -ATTTTGTTTTTCTAATAAAATACCTCATATTTATCATTATTATTATTGTTATAA-------AAGAAATCACCATGA-TTTGTATCCTCTTGTTGTCAAC

2410 2420 2430 2440 2450 2460 2470 2480 2490 2500

....|....|....|....|....|....|....|....|....|....|....|....|....|....|....|....|....|....|....|....|

***PtSand*** 2195 GGCTTGCATACAGTTGCAGCCTCATGTTGTC---ACCAGAAATTCTCAACGAATATTAGATTTGGTGTACACTCCTGTGGAATTGGGTATAAGTTGGGAA

***AtSand*** 2055 TATTTCATTGCCTTTATTGTTATTAATTA-----GCTTGAATAGTCAAAATTGAGATGATGTAGACAAAAATTGAAACTATTTGAAAAGTGAGCAATAAT

***PsSand*** 2378 AGTCAGTGTATGTTGGTTCCGCTTTGTTGCTTTTCTTATAAAATATCATGTACAGCTCCTTTAAGAGGAA---TCTGTGGCATTCCTTTCAGGTGAGACA

***VvSand*** 2023 --CCCGCTTGTA--TACAAAAGTATATTG-----AATAAATTATCTGAGGCAGTATTCATTTCAGTAA-------TTTAGTATCTAGCGCAA-CAGCCAT

1. *Β-Tub* gene*.* Species and accession numbers used: *Populus trichoparpa* (XM_002298000.1), *Gossypium hirsutum* (AF521240.1), *Medicago truncatula* (XM_003630465.1), *Nicotiana tabacum* (EF051136.2), *Ricinus communis* (XM_002509755.1), *Theobroma cacao* (GU570572.1), *Vitis vinifera* (XM_002273478.2).

10 20 30 40 50 60 70 80 90 100

....|....|....|....|....|....|....|....|....|....|....|....|....|....|....|....|....|....|....|....|

***PtTub*** 1 --------------------------GGCTCTCCTCTACTCTT-------TTTCTCTACATTTCC--CGTCTTAACATTACCATT---------GTTGAT

***GhTub*** 1 ----------------------------------------------------------------------------------------------------

***MtTub*** 1 ---------------------------------------------------------GTACTTGATCTAACTCGGTGTTACAATTTTTTCCTTGAGTCTT

***NtTub*** 1 ------------------------------------------------------------CACAGAAAATCCACCAACTATCCTCTCCCTTTCTCCTCCC

***RcTub*** 1 --AAAGGCCTTGCACCATCCCATTCTTTCTCATCCCTCCCTCTCTAGTGCTTTGATTCATTTTGTAAGGCCAAGAAAAACCCATTAGCCATATTAAAAAA

***TcTub*** 1 ----------------------------------------------------------------------------------------------------

***VvTub*** 1 ATCCCCATCGATATTTCTCTCTTCTTTGCTCTCTTCTTCTCTTATATATCTCCCTTTGAGTTTCCAACTTTTTGTCACAGCCTTTTGTTCCCAAGCCAAT

110 120 130 140 150 160 170 180 190 200

....|....|....|....|....|....|....|....|....|....|....|....|....|....|....|....|....|....|....|....|

***PtTub*** 57 T-ATTAATCCA------------GATGAGAGAAATCCTCCATATTCAAGCTGGTCAATGTGGTAACCAGATTGGTGGCAAGTTCTGGGAGGTTGTGTGTG

***GhTub*** 1 ------------------------ATGAGAGAAATCCTCCATGTTCAAGCCGGTCAGTGTGGTAATCAAATTGGTGGCAAGTTTTGGGAAGTAGTATGTG

***MtTub*** 44 GAAATCATCAAGTTCAT------AATGAGAGAAATCCTTCATGTACAAGCAGGTCAATGTGGAAATCAAATTGGAGGAAAGTTTTGGGAGGTTATGTGTG

***NtTub*** 41 TCCTTCGAACGAAACCCTAATAAAATGCGTGAAATCCTCCATATCCAAGGTGGCCAATGTGGCAACCAAATTGGGGCCAAGTTCTGGGAGGTTGTGTGCG

***RcTub*** 99 ACTATAATCTT--GTATAGAGAAAATGAGAGAAATTCTCCATATCCAAGCTGGTCAATGTGGTAACCAAATTGGAGGCAAGTTTTGGGAAGTTGTATGTG

***TcTub*** 1 ----------------------------------------------------------------------------------------------------

***VvTub*** 101 TGAGCAATCCATATCATTCTGAGAATGAGAGAAATTCTCCATATCCAAGCTGGGCAATGCGGGAACCAAATTGGTGGCAAGTTTTGGGAGGTGGTATGTG

210 220 230 240 250 260 270 280 290 300

....|....|....|....|....|....|....|....|....|....|....|....|....|....|....|....|....|....|....|....|

***PtTub*** 144 ATGAACATGGGATTGATCCGACGGGGAATTACACTGGCAACTCTCACGTTCAACTTGAGAGGGTCAATGTTTACTACAATGAGGCTAGTGGTGGCCGCTA

***GhTub*** 77 ATGAACATGGGATAGATGCCACTGGTAACTATGTCGGCACTTCGCCTGTTCAGCTTGAAAGGCTTAATGTTTACTATAATGAAGCCAGTGGTGGCAGATA

***MtTub*** 138 ATGAACATGGGATAGATCCTTCAGGAAGTTATGTAGGAAAGTCACACCTTCAACTTGAGAGAGTGAATGTGTACTACAATGAAGCAAGTGGTGGAAGATA

***NtTub*** 141 CGGAGCACGGGATCGATTCCACCGGCGCGTACCATGGGGAATCGGATATTCAACTTGAGAGGGTAAATGTCTATTATAATGAGGCGAGTTGTGGGCGTTT

***RcTub*** 197 ATGAACATGGTATTGATCCTACAGGAAATTATGTTGGCAACTCCCATGTTCAACTTGAGAGAGTTAATGTTTACTACAACGAAGCTAGTGGTGGCAGGTA

***TcTub*** 1 ----------------------------------------------------------------------------------------------------

***VvTub*** 201 ATGAGCACGGCATCGATACCAAGGGGAATTACGTTGGTGATTCTCATCTGCAGCTTGAGAGGGTGAATGTCTACTACAATGAGGCCAGTGGCGGACGGTA

310 320 330 340 350 360 370 380 390 400

....|....|....|....|....|....|....|....|....|....|....|....|....|....|....|....|....|....|....|....|

***PtTub*** 244 TGTGCCCAGGGCTGTCCTAATGGACCTTGAGCCAGGGACCATGGACAGCTTGAGGACTGGTCCCTATGGGAAAATCTTTAGGCCTGACAATTTTGTTTTC

***GhTub*** 177 TGTGCCTAGAGCTGTGTTAATGGATCTTGAGCCAGGAACTATGGACAGTTTACGAACAGGTCCTTATGGAAAATTGTTTAGACCAGATAACTTTGTGTTC

***MtTub*** 238 TGTTCCTAGAGCTGTTCTAATGGACCTTGAACCAGGTACCATGGACAGTTTACGTTCTGGTCCATTTGGAAAAATATTTAGGCCTGATAACTTTGTGTTT

***NtTub*** 241 TGTACCTCGTGCTGTTCTTATGGATTTAGAGCCTGGTACTATGGACAGTGTTAGATCTGGGCCTTATGGTCAGATTTTTAGGCCTGACAACTTTGTTTTT

***RcTub*** 297 CGTGCCTAGAGCTGTGTTAATGGATCTTGAACCAGGTACCATGGACAGCTTAAGGACTGGTCCTTATGGTAAAATCTTTAGGCCTGACAATTTTGTTTTT

***TcTub*** 1 -------------------ATGGATCTCGAGCCGGGAACTATGGATAGTGTGAGGACTGGACCTTACGGACAGATCTTTAGGCCCGATAACTTCGTGTTT

***VvTub*** 301 TGTGCCTAGAGCTGTGCTCATGGACCTCGAGCCAGGGACCATGGACAGCTTGAGGACTGGCCCCTATGGCAAAATCTTTAGGCCTGATAACTTTGTGTTC

410 420 430 440 450 460 470 480 490 500

....|....|....|....|....|....|....|....|....|....|....|....|....|....|....|....|....|....|....|....|

***PtTub*** 344 GGCCAAAATGGAGCTGGAAATAACTGG-CTAAAGGACATTACACTGAAGGAGCTGAACTGATCGATTCTGTTCTTGATGTTGTTCGAAAAGAGGCTGAGA

***GhTub*** 277 GGCCAAAATGGAGCTGGCAATAACTGGGCTAAGGGACATTATACTGAAGGAGCTGAATTGATTGATTCAGTTCTTGATGTTGTTCGTAAAGAGGCTGAGA

***MtTub*** 338 GGACAAAATGGAGCTGGTAATAATTGGGCTAAAGGACATTACACTGAAGGAGCTGAACTTATTGATTCTGTTCTTGATGTTGTTCGAAAAGAAGCTGAGA

***NtTub*** 341 GGCCAGTCTGGT-CGGGAAATAATTGGGCTAAGGGTCATTACACTGAGGGCGCTGAGTTGATTGATTCGGTTCTCGATGTTGTTCGTAAAGAAGCCGAGA

***RcTub*** 397 GGCCAAAATGGAGCTGGTAATAACTGGGCTAAAGGGCATTATACCGAAGGAGCAGAATTGATCGACTCTGTTCTTGATGTAGTTCGTAAAGAAGCTGAGA

***TcTub*** 82 GGACAATCTGGAGCTGGGAATAATTGGGCTAAGGGGCATTACACTGAAGGAGCTGAGCTTATTGATGCTGTTCTTGATGTTGTTAGAAAGGAGGCTGAGA

***VvTub*** 401 GGCCAAAACGGAGCTGGAAACAACTGGGCCAAGGGGCATTACACTGAGGGGGCAGAGCTGATTGATTCTGTTCTAGATGTTGTTCGCAAAGAGGCCGAGA

510 520 530 540 550 560 570 580 590 600

....|....|....|....|....|....|....|....|....|....|....|....|....|....|....|....|....|....|....|....|

***PtTub*** 443 ATTGTGATTGCTTACAAGGCTTCCAAATCTGTCATTCTCTGGGAGGTGGAACTGGATCAGGAATGGGGACTCTGCTCATATCAAAGATCAGGGAAGAATA

***GhTub*** 377 ATTGTGATTGTTTACAAGGTTTTCAGGTTTGCCATTCACTGGGAGGTGGAACAGGGTCAGGGATGGGGACATTGTTGATATCAAAGATCAGGGAAGAATA

***MtTub*** 438 ATTGCGACTGCTTGCAAGGTTTTCAAATTTGTCATTCGCTTGGAGGTGGAACTGGATCAGGAATGGGTACCTTGCTCATTTCCAAGATCAGAGAAGAGTA

***NtTub*** 440 ATTGTGATTGCCTACAAGGGTTTCAGGTGTGCCATTCCCTGGGAGGAGGGACTGGGTCTGGAATGGGGACACTTCTCATTTCAAAGATAAGAGAGGAATA

***RcTub*** 497 ATTGTGATTGCTTACAAGGCTTCCAAATCTGCCATTCTTTGGGAGGTGGAACTGGGTCAGGAATGGGAACTCTACTCATATCAAAGATCAGGGAAGAGTA

***TcTub*** 182 ATTGTGACTGTCTCCAAGGTTTTCAAGTTTGCCACTCTCTGGGTGGAGGAACTGGTTCCGGGATGGGTACCCTGTTGATCTCAAAGATCAGAGAAGAATA

***VvTub*** 501 ATTGTGATTGCTTACAAGGCTTCCAGATTTGCCATTCCCTCGGCGGCGGCACTGGATCCGGAATGGGAACCCTGCTTATATCCAAGATCAGAGAAGAATA

610 620 630 640 650 660 670 680 690 700

....|....|....|....|....|....|....|....|....|....|....|....|....|....|....|....|....|....|....|....|

***PtTub*** 543 CCCTGATAGGATGATGTTAACTTTCTCAGTATTTCCATCTCCCAAGGTTTCTGATACTGTGGTTGAGCCCTACAATGCAACCCTCTCTGTACACCAACTA

***GhTub*** 477 CCCTGACCGGATGATGCTAACGTTCTCGGTGTTTCCATCACCTAAAGTATCGGATACTGTGGTTGAACCCTATAATGCGACCCTGTCAGTGCACCAGCTT

***MtTub*** 538 TCCAGACAGAATGATGTTGACTTTCTCAGTGTTCCCTTCACCAAAGGTTTCTGATACCGTGGTGGAACCCTACAATGCAACTCTCTCTGTTCATCAACTA

***NtTub*** 540 CCCAGACAGGATGATGCTGACATTCTCTGTTTTCCCATCTCCAAAGGTCTCGGACACTGTTGTAGAGCCTTACAATGCAACCTTGTCTGTTCATCAGCTT

***RcTub*** 597 CCCAGATAGGATGATGTTAACTTTCTCTGTTTTTCCTTCTCCTAAGGTTTCTGATACAGTGGTTGAGCCCTACAATGCAACCTTGTCTGTGCACCAGTTA

***TcTub*** 282 CCCTGATAGAATGATGCTCACTTTCTCTGTCTACCCATCACCAAAGGTTTCAGATACAGTGGTTGAGCCATACAATGCCACCCTTTCTGTTCATCAGCTT

***VvTub*** 601 CCCTGATCGGATGATGCTCACTTTCTCCGTCTTCCCTTCACCCAAGGTCTCTGACACCGTCGTTGAGCCCTACAACGCCACCCTCTCCGTCCACCAACTC

710 720 730 740 750 760 770 780 790 800

....|....|....|....|....|....|....|....|....|....|....|....|....|....|....|....|....|....|....|....|

***PtTub*** 643 GTTGAAAATGCTGATGAGTGTATGGTCCTTGACAACGAGGCTCTCTATGATATCTGCTTTCGAACTCTCAAGCTCACCAATCCAAGCTTTGGTGATCTTA

***GhTub*** 577 GTGGAAAATGCTGATGAATGCATGGTCCTTGACAATGAAGCTCTTTATGATATCTGCTTCAGAACTCTTAAGCTCACAAATCCCAGCTTTGGTGACTTGA

***MtTub*** 638 GTTGAAAATGCAGACGAATGCATGGTTCTTGATAATGAGGCACTCTATGATATCTGTTTCAGAACACTCAAGCTCACTAATCCAAGTTTTGGTGATTTGA

***NtTub*** 640 GTGGAGAATGCAGATGAGTGCATGGTTCTTGATAATGAGGCACTCTATGACATTTGTTTCCGTACCCTCAAACTTACAACTCCTAGCTTTGGTGATCTGA

***RcTub*** 697 GTTGAAAATGCTGATGAGTGTATGGTACTTGACAATGAAGCACTCTATGATATCTGCTTCCGAACTCTCAAGCTCACCAATCCTAGCTTTGGTGATCTGA

***TcTub*** 382 GTTGAGAATGCTGATGAGTGCATGGTGTTGGACAATGAGGCTTTGTATGATATCTGTTTCAGGACCCTGAAGTTAACTACTCCTAGCTTTGGTGATCTGA

***VvTub*** 701 GTGGAGAACGCCGACGAGTGCATGGTCCTCGACAATGAAGCTCTCTACGACATTTGCTTCCGAACTCTCAAGCTCACCAATCCAAGCTTTGGGGATTTGA

810 820 830 840 850 860 870 880 890 900

....|....|....|....|....|....|....|....|....|....|....|....|....|....|....|....|....|....|....|....|

***PtTub*** 743 ACCACTTGATCTCGACAACCATGAGTGGAGTAACATGTTGCCTTCGATTCCCGGGCCAATTGAACTCTGATCTTCGAAAACTAGCCGTGAACTTAATCCC

***GhTub*** 677 ACCATTTGATTTCAACAACCATGAGTGGAGTCACATGCTGCCTTCGCTTCCCTGGCCAACTCAATTCCGATCTTCGAAAACTAGCAGTAAACTTGATCCC

***MtTub*** 738 ACCATTTGATATCAACAACAATGAGTGGAGTAACATGTTGCCTCCGATTTCCTGGCCAACTCAACTCTGATCTTAGGAAATTAGCAGTTAACCTCATCCC

***NtTub*** 740 ATCACTTAATATCTGCAACCATGTCTGGAGTTACTTGTTGCCTCAGATTCCCTGGCCAGCTTAACTCTGATCTGCGGAAACTTGCTGTGAATCTCATTCC

***RcTub*** 797 ACCATTTGATCTCTACAACCATGAGTGGAGTAACATGTTGTCTTCGCTTCCCGGGTCAGCTAAACTCTGATCTTCGAAAACTAGCTGTAAATTTAATCCC

***TcTub*** 482 ACCATCTGATCTCTGCAACCATGAGTGGTGTCACATGCTGCCTTAGATTCCCTGGCCAGCTCAACTCTGACCTCCGAAAACTTGCAGTGAACCTCATTCC

***VvTub*** 801 ACCATTTGATCTCCACCACCATGAGCGGCGTAACATGCTGCCTCCGCTTCCCCGGCCAGCTCAACTCCGACCTCCGAAAACTGGCGGTCAATCTTATCCC

910 920 930 940 950 960 970 980 990 1000

....|....|....|....|....|....|....|....|....|....|....|....|....|....|....|....|....|....|....|....|

***PtTub*** 843 CTTCCCACGTCTCCATTTCTTCATGGTTGGTTTTGCACCATTAACCTCCCAAGGCTCACAACAGTACCGTGCCTTAACCATCCCGGAGCTGACACAACAA

***GhTub*** 777 ATTCCCACGCCTCCATTTTTTCATGGTTGGTTTTGCACCTTTAACATCCCGGGGTTCACAACAATACCGAGCTTTAACGATCCCCGAGCTAACTCAACAA

***MtTub*** 838 TTTCCCACGTCTACACTTTTTCATGGTTGGTTTTGCTCCCTTAACATCAAGGGGTTCTCAACAGTACAGTTCCCTCACCATTCCAGAACTCACACAGCAA

***NtTub*** 840 TTTCCCCCGTCTTCACTTTTTCATGGTTGGGTTTGCTCCACTTACCTCACGTGGTTCACAACAATACCGAGCTTTATCTGTCCCTGAGCTTACTCAGCAA

***RcTub*** 897 CTTCCCGCGTCTCCACTTCTTTATGGTAGGATTTGCACCCCTGACCTCTCGTGGCTCGCAACAGTACCGAGCCCTAACAATCCCTGAGCTCACACAGCAA

***TcTub*** 582 TTTCCCTCGTCTGCACTTCTTTATGGTTGGGTTTGCTCCTCTCACCTCGAGGGGATCTCAGCAGTATCGTGCTCTAACTGTCCCAGAACTCACCCAGCAA

***VvTub*** 901 ATTCCCGCGATTACACTTCTTCATGGTGGGTTTTGCACCCCTGACGTCGCGTGGATCACAGCAGTACCGGGCCCTCACCATCCCGGAGCTGACACAGCAA

1010 1020 1030 1040 1050 1060 1070 1080 1090 1100

....|....|....|....|....|....|....|....|....|....|....|....|....|....|....|....|....|....|....|....|

***PtTub*** 943 ATGTGGGATGCTAAAAACATGATGTGTGCAGCTGACCCTCGGCACGGTAGGTACTTAACAGCCTCAGCTATGTTTCGAGGCAAAATGAGCACTAAGGAAG

***GhTub*** 877 ATGTGGGATTCCAAAAACATGATGTGCGCGGCTGATCCTCGTCATGGAAGGTACTTAACAGCCTCGGCAATGTTTCGAGGCAAAATGAGCACCAAGGAAG

***MtTub*** 938 ATGTGGGATGCAAGAAACATGATGTGTGCTGCTGATCCAAGACATGGTAGGTATCTAACAGCCTCGGCAATGTTCCGTGGCAAAATGAGCACAAAAGAAG

***NtTub*** 940 ATGTGGGATGCAAAGAACATGATGTGTGCTGCTGACCCTAGGCATGGCCGCTATTTGACAGCATCAGCTATGTTTAGGGGGAAGATGAGCACCAAGGAAG

***RcTub*** 997 ATGTGGGATGCTAAGAACATGATGTGTGCAGCTGACCCGCGGCACGGGAGATACCTGACAGCCTCAGCCATGTTCCGTGGCAAGATGAGCACTAAAGAAG

***TcTub*** 682 ATGTGGGATGCTAAAAATATGATGTGTGCTGCAGACCCACGACATGGTCGCTACCTCACAGCCTCAGCCATGTTCAGGGGGAAGATGAGCACCAAAGAGG

***VvTub*** 1001 ATGTGGGATGCGAAGAACATGATGTGTGCGGCTGACCCGCGACACGGCCGGTACCTGACCGCCTCAGCGATGTTCCGGGGGAAGATGAGTACTAAAGAGG

1110 1120 1130 1140 1150 1160 1170 1180 1190 1200

....|....|....|....|....|....|....|....|....|....|....|....|....|....|....|....|....|....|....|....|

***PtTub*** 1043 TTGATGAGCAGATGATGAATGTGCAAAACAAGAACTCATCATATTTTGTTGAGTGGATTCCAAATAATGTTAAATCAAGTGTTTGTGACATTCCACCAAC

***GhTub*** 977 TTGATGAACAAATGATCAATGTCCAAAACAAGAACTCTTCGTACTTTGTGGAGTGGATTCCGAACGATGTTAAATCAAGTGTTTGCGACATCCCACCAAC

***MtTub*** 1038 TTGATCAACAGATGATAAATGTTCAGAACAAGAACTCGTCTTACTTTGTGGAATGGATTCCAAACAATGTGAAATCAAGTGTTTGTGATATTCCGCCAAC

***NtTub*** 1040 TTGATGAGCAGATGCTTAACGTGCAGAACAAAAATTCATCATACTTTGTTGAGTGGATCCCCAACAATGTCAAATCAACTGTCTGTGATATTCCACCAAC

***RcTub*** 1097 TTGATGAACAAATGATAAACGTACAAAATAAGAACTCATCTTACTTTGTTGAGTGGATTCCAAACAATGTCAAATCAAGTGTATGTGACATTCCACCAAC

***TcTub*** 782 TTGATGAGCAAATGATTAATGTTCAAACCAAGAACTCTTCATACTTTGTTGAGTGGATTCCAAACAATGTGAAGTCTAGTGTGTGTGATATTCCTCCTGA

***VvTub*** 1101 TTGATGAGCAGATGATCAATGTGCAGAACAAGAACTCCTCGTACTTTGTTGAGTGGATACCAAACAATGTGAAGTCAAGCGTCTGTGACATCCCTCCGAC

1210 1220 1230 1240 1250 1260 1270 1280 1290 1300

....|....|....|....|....|....|....|....|....|....|....|....|....|....|....|....|....|....|....|....|

***PtTub*** 1143 TGGGTTAGCAATGTCATCACACATTTATGGGAAATTCTACGTCTATTCAAGAAATGTTTAGGCGTGTTTCGGAACAATTTACAGTTATGTTTAGGAGAAA

***GhTub*** 1077 TGGGTTGACGATGTCATCG-ACGTTTATGGGGAACTCGACATCGATACAAGAGATGTTTCGACGTGTTTCGGAACAGTTCACAGTGATGTTTAGGAGGAA

***MtTub*** 1138 AGGGTTGTCGATGTCTTCG-ACATTTATGGGGAATTCGACATCTATTCAAGAAATGTTTAGACGTGTTTCGGAGCAGTTTACTGTTATGTTTAAGAGAAA

***NtTub*** 1140 TGGTCTGAAGATGGCATCA-ACTTTCATTGGAAACTCAACATCAATTCAAGAGATGTTCCGTCGTGTCAGTGAGCAATTCACAGCCATGTTTAGGAGGAA

***RcTub*** 1197 AGGGTTATCCATGTCATCA-ACATTTATGGGAAATTCAACGTCTATTCAAGAAATGTTCAGGCGTGTATCAGAACAATTTACGGTCATGTTTAGGAGGAA

***TcTub*** 882 GGGCCTGTCTATGGCATCA-ACTTTCATTGGTAACTCAACCTCCATTCAGGAGATGTTCAGGCGAGTGAGTGAGCAATTCACTGCCATGTTTAGGAGGAA

***VvTub*** 1201 TGGGTTGGCCATGTCGTCG-ACGTTCATGGGGAACTCCACGTCCATCCAGGAGATGTTCCGCCGGGTGTCGGAGCAGTTCACGGTCATGTTCAGGAGGAA

1310 1320 1330 1340 1350 1360 1370 1380 1390 1400

....|....|....|....|....|....|....|....|....|....|....|....|....|....|....|....|....|....|....|....|

***PtTub*** 1243 AGCGTTTTTGCACTGGTACACTGGGGAAGGAATGGATGAAATGGAGTTTACTGAGGCTGAAAGTAACCATGAACGATTTGGTTTCTGAATATCACACAAT

***GhTub*** 1176 AGCATTTTTGCATTGGTATACAGGGGAAGGGATGGATGAAATGGAGTTTACTGAGGCTGAAAGTAAT-ATGAATGATTTGGTTTCTGAATATCA-ACAAT

***MtTub*** 1237 GGCATTTTTGCATTGGTATACTGCTGAAGGAATGGATGAGATGGAGTTTACTGAGGCTGAGAGTAAT-ATGAATGATTTGGTTTCTGAATATCA-ACAAT

***NtTub*** 1239 GGCTTTCTTGCACTGGTACACTGGGGAAGGAATGGATGAGATGGAGTTCACTGAGGCAGAGAGTAAC-ATGAATGATCTGGTCTCAGAGTACCA-GCAGT

***RcTub*** 1296 GGCTTTTCTGCATTGGTACACTGGGGAAGGAATGGATGAAATGGAGTTTACTGAGGCAGAAAGCAAT-ATGAATGATTTGGTATCAGAATATCA-ACAGT

***TcTub*** 981 GGCTTTCTTGCACTGGTACACTGGGGAAGGAATGGATGAAATGGAGTTCACTGAGGCTGAGAGCAAC-ATGAATGCCCTTGTATCTGAATATCA-GCAGT

***VvTub*** 1300 GGCGTTTTTGCATTGGTACACTGGAGAGGGAATGGATGAGATGGAGTTCACAGAGGCGGAGAGCAAT-ATGAATGATCTGGTGTCAGAGTATCA-GCAGT

1410 1420 1430 1440 1450 1460 1470 1480 1490 1500

....|....|....|....|....|....|....|....|....|....|....|....|....|....|....|....|....|....|....|....|

***PtTub*** 1343 ATCAAGATGCCG---CAGCCGATAATGAGGGG------GAGTATGATGAAGAAGAGCCTATGGAG----AACTAA---GGAGAATTTGATC-TGGTTATT

***GhTub*** 1274 ATCAAGATGCTGTGGTTGATGAAGATGGTGAA------GGGTATGAAGATGAA---GCTGAGGAA----AATTGAC--GGAATTCTTTTCAACTTTCTAT

***MtTub*** 1335 ATCAAGATGCTGCTGGAGTGGAAGAAGGTGAGTTTGATGAAGATGATGAAGAGGAAATTGCCTAGTTGCAATGACTTTGACACAAACATTATTTAAGGTT

***NtTub*** 1337 ACCAAGATGCAG---TAGCAGATGAGGATGAA------GGATATGAGGATGAAGAGGAAGCATAT----CATGAAT--AGTGTGC-TGATGCCTGGAAA-

***RcTub*** 1394 ATCAAGATGCAGGGGCGGAGCATGATGAGGAT------GAGGATGAGGATGGAGAGGTTGAGGAG----AACTGAA--AATGGAGATTCTTGGTTTCGA-

***TcTub*** 1079 ACCAGGATGCTA---CAGCTGATGAGGAACTT------GAATATGAGGAGGAGGAGGAGGAGGAG----GAGGAA---GGTGTTCATGAGATGTGAGAGA

***VvTub*** 1398 ACCAGGAGGCGGTGGCGGCGGAGGATGAGGAA------GAGTACGATGAAGAAGTG---ATGGAG----AACTAGATTGGTGGCGGTGGTGGTGGTTGAT

1510 1520 1530 1540 1550 1560 1570 1580 1590 1600

....|....|....|....|....|....|....|....|....|....|....|....|....|....|....|....|....|....|....|....|

***PtTub*** 1426 TTCCTATGGC------CATGGCTTCAAGGATGAGTTGTTTC-TGGTGGAGTTATTTATGTT---ACTGTATGGAGGTCAATATTTGAATAACTTGGGCCT

***GhTub*** 1359 AGTAATGGCC------TAGGAAAAAAAAATCAAATATGGTGCTCATTTTGATGGTTCTAAATAAGGAGGCCATGTTTTTTCATTTGTG---CTGGGATTT

***MtTub*** 1435 CTTTTTGGATCATTCATGTCATGGCAAATTAACACATATCATAAGTTTGTTTGGTTGTGTGT--GCTACACATATATATTTGGTAATGGCATGGTGAAAA

***NtTub*** 1419 ----------------TCTTATCTTATATTTTAAGCAATAGT--TCCTTTCAACTGTGGATGTAACCTCAACTACCATACCCATAGGAGGAGATTGAACT

***RcTub*** 1480 ----ATTGGC------TATAAGACAAAAAT-GTATGTGTTATTCTTTTTG--GGTTGGGTCGAGGGTGGGTGTGTTTTAT-GTTTGTT---GTTTGATGA

***TcTub*** 1163 TGTAAAGGCTG---TGTGCTACTTTATATTGTGGACTGTGGTAATGCCTCGATATACTGCTTGAGAGTTAAAAAGTGCAATTTTCTGTT-AGATTGAGTG

***VvTub*** 1485 GTCACTGGGT------TATGGGTGTGGGGTTTGGCTGGCAAATAATCGAGGAGGGTGGGGT---GCT-TGTGTAT-TCAATATTTGGG--GTTTGGTATC

1610 1620 1630 1640 1650 1660 1670 1680 1690 1700

....|....|....|....|....|....|....|....|....|....|....|....|....|....|....|....|....|....|....|....|

***PtTub*** 1516 CTGATGACCGTGAGATTGTGAATAT----TTCTATTTATAATCTGTGAGT-ATAAAATT-TGTA--TTACTGTTTTG----TTATAATGTGGATGATTA-

***GhTub*** 1450 TTAGTAATGTGAATTGTGAATTTGTA--TATTTATATCAAATGTGAA---TTGTGATTT-AATGAATTTGGAGTGTA-AATTTATG-TAATGAATTTGGA

***MtTub*** 1533 AAGAAGGTAAAGAATGTGTGTACATGAATAATTTACTCATATATATTATGAGTTGGCTTGCAAATGTGAAAAGTACA----TAGGATTTGGGGCTATCAA

***NtTub*** 1502 TTAGTAGTGAAGAGAAAGCG-----A-----CTAACCTGT--ATTTT---TGTTGAACT-T--GTCTCACTATCC-------TTTAATTGCAA-ATTGAA

***RcTub*** 1564 TGGTAAATAAGGAGGTCATGTTTACAG-TGTCCAGGGCTCCTGTAACCCTTTCTGTAAA-AAGGAATTTTTTTTATG-GCTTTGTGATTATTTATATGTA

***TcTub*** 1259 GTAACATATGTGAAAAGCTGTTTTTG-----TTACTCTTTTTATTCT---GTTTGAATT-CTAGCCTTGCTGTTCAA-----TTCGTTTGCTATAATGAA

***VvTub*** 1572 CCTATGATTGTGAATTTGTGATTTTAT-TTTTTATTTTTTGTCCGTTATTTATGTGATT-TGTGAGTTGTGAGTATAAGTTTTGTACTTGAAACAAGTAA

1710 1720 1730 1740 1750 1760 1770 1780 1790 1800

....|....|....|....|....|....|....|....|....|....|....|....|....|....|....|....|....|....|....|....|

***PtTub*** 1602 -TCGTTTTTCTTCATCTATTT-TACATTATCTACATTTTTTCTCCTAATAATATTAATTTT---------------------------------------

***GhTub*** 1542 ATCACAAGTTTCAAAATTTCAAGACAAAAAAAAAAAAAA-------------------------------------------------------------

***MtTub*** 1629 TTCTCA-TGGGAGTGTTGTTTTTATTTTGAATCGTCTGTTTGTTCTTGTCTTCTATCTTATCTTGTTAATTGATATGATCAATTTGTATAGTCGGTGGAA

***NtTub*** 1576 TATTTTGTGAAAAAAAAAAAAAAAAAAAAAAAA-------------------------------------------------------------------

***RcTub*** 1661 ATTTGA--TTACATGTCTTATATATATTATATTA------------------------------------------------------------------

***TcTub*** 1345 AGTTAATAGTATTTGTTAAAAAAAAAAAAAAAA-------------------------------------------------------------------

***VvTub*** 1670 ATCACTCTGTTAAAAGGGCTTGTTCATCAGACCCCTTCATTTCCCTTCTCCTCTTAAGCTTCCAACTCAAAAGGAGAAAAACCAA---------------

1. *Ubq* gene*.* Species and accession numbers used: *Populus trichoparpa* (XM_002320914.1), *Hevea brasiliensis* (EF120638.1), *Medicago truncatula* (XM_003629847.1), *Nicotiana tabacum* (DQ138111.1), *Pyrus communis* (AF386524.1), *Ricinus communis* (XM_002515167.1), *Solanum tuberosum* (L22576.1)

10 20 30 40 50 60 70 80 90 100

....|....|....|....|....|....|....|....|....|....|....|....|....|....|....|....|....|....|....|....|

***PtUbq*** 1 ----------------------------------GCTCTCTTATACAAAACA-CCATCAGCCGAAA-CCCGCTGTTCCCCATCCGCTCTGCATCTCCAAA

***HbUbq*** 1 ----------------------------ACGCGGGGACGCTTCAACCATTCAACCCTAAGCCGAAAACCCTCTTCTCGCCTTGCTTTCTGCATC------

***MtUbq*** 1 --------------------------------GAAACCCTAGAAGATAAATTCCGAGAAACCCTAATCGCCTTCATTCCGAAACCATTCGTTTGTGAGAG

***NtUbq*** 1 ----------------------------------------------------------------------------------------------------

***PcUbq*** 1 ----------------------------------------------------------------------------------------------------

***RcUbq*** 1 -------------------------------TGAAACCCTAG----------CCGAAAAGACCTCTTCAT----ATTCCTACACT-CTCTTTTCT-----

***StUbq*** 1 CGAAGAAAAGGGCTTGTAAAACCCTAATAAAGTGGCACTGGCAGAGCTTACACTCTCATTCCATCAACAAAGAAACCCTAAAAGCCGCAGCGCCACTGAT

110 120 130 140 150 160 170 180 190 200

....|....|....|....|....|....|....|....|....|....|....|....|....|....|....|....|....|....|....|....|

***PtUbq*** 65 AACCCCCAAG-AGGCGA---AGATGCAGATCTTCGTGAAGACCTTGACGGGAAAGACCATAACCCTCGAGGTTGAGTCATCAGACACAATCGACAATGTC

***HbUbq*** 67 GGCCATCAA-----------AGATGCAGATCTTCGTCAAAACCCTAACGGGTAAGACCATAACTCTCGAGGTAGAGTCCTCGGACACTATCGACAATGTG

***MtUbq*** 69 TTGCAGCAACCAACGAAGCAAGATGCAGATCTTCGTGAAAACCCTAACAGGGAAGACGATAACCCTCGAGGTTGAGTCTTCCGACACAATCGACAATGTC

***NtUbq*** 1 ----------------------ATGCAGATATTCGTGAAGACCCTGACGGGGAAGACTATTACCTTAGAGGTAGAGTCATCGGACACCATTGACAATGTT

***PcUbq*** 1 --------------CAA---CCATGCAGATCTTCGTGAAAACCCTAACGGGTAAGACCATAACCCTAGAGGTCGAGTCCTCCGATACCATTGACAATGTC

***RcUbq*** 50 CTGCATCGGT-GGCGGA--AAGATGCAGATCTTCGTGAAAACCCTAACGGGTAAGACCATAACCCTAGAGGTTGAATCCTCCGATACCATCGACAATGTG

***StUbq*** 101 TTCTCTCCTCCAGGCGA---AGATGCAGATCTTCGTGAAGACCTTAACGGGGAAGACGATCACCCTAGAGGTTGAGTCTTCCGACACCATCGACAATGTC

210 220 230 240 250 260 270 280 290 300

....|....|....|....|....|....|....|....|....|....|....|....|....|....|....|....|....|....|....|....|

***PtUbq*** 161 AAAGCCAAGATTCAGGACAAGGAGGGCATCCCTCCAGACCAACAGCGTCTCATTTTCGCTGGGAAACAACTCGAGGACGGTCGCACCCTCGCTGACTACA

***HbUbq*** 156 AAGGCCAAGATCCAAGACAAGGAGGGCATCCCACCGGACCAGCAGCGCCTCATCTTCGCCGGAAAGCAACTGGAAGACGGAAGGACCCTTGCCGACTACA

***MtUbq*** 169 AAAGCCAAGATCCAGGATAAGGAAGGAATTCCACCTGACCAGCAACGTCTCATCTTCGCCGGAAAGCAGTTGGAAGACGGACGCACTCTCGCCGACTACA

***NtUbq*** 79 AAGGCTAAGATTCAGGACAAGGAAGGCATTCCACCGGACCAGCAGCGGTTGATTTTCGCAGGTAAGCAGCTTGAGGATGGCCGAACACTAGCTGACTACA

***PcUbq*** 84 AAGGCCAAGATCCAAGACAAGGAGGGCATCCCCCCGGACCAGCAGCGCCTCATCTTCGCCGGCAAGCAGCTCGAGGACGGCCGAACCCTCGCCGACTACA

***RcUbq*** 147 AAGGCCAAGATCCAAGACAAGGAAGGCATCCCACCGGACCAGCAACGGCTAATCTTCGCAGGAAAGCAACTCGAAGACGGCCGTACACTTGCGGACTACA

***StUbq*** 198 AAAGCCAAGATCCAGGACAAGGAAGGGATTCCCCCAGACCAGCAGCGTTTGATTTTCGCCGGAAAGCAGCTTGAGGATGGTCGTACTCTTGCCGACTACA

310 320 330 340 350 360 370 380 390 400

....|....|....|....|....|....|....|....|....|....|....|....|....|....|....|....|....|....|....|....|

***PtUbq*** 261 ACATCCAGAAGGAGTCCACTCTCCACTTGGTGCTTCGCCTGAGGGGTGGAGCCAAGAAGAGAAAGAAGAAGACCTACACCAAGCCCAAGAAGATCAAGCA

***HbUbq*** 256 ATATTCAGAAGGAGTCGACTCTCCACCTAGTGTTGCGTTTGAGGGGTGGAGCCAAGAAGAGGAAGAAGAAGACCTATACCAAACCCAAGAAGATCAAGCA

***MtUbq*** 269 ACATCCAGAAGGAATCCACTCTTCACCTTGTCCTACGTCTTCGTGGTGGCGCTAAGAAGCGTAAGAAGAAGACCTACACCAAGCCTAAGAAGATCAAGCA

***NtUbq*** 179 ACATCCAGAAGGAGTCCACCCTCCATCTTGTCCTTCGCCTCCGTGGTGGTGCAAAGAAGCGTAAGAAGAAGACTTACACTAAGCCAAAGAAAATCAAGCA

***PcUbq*** 184 ACATCCAGAAGGAGTCCACTCTCCACCTGGTGCTCCGCCTCCGCGGTGGCGCCAAGAAGAGGAAGAAGAAGACCTACACCAAGCCCAAGAAGATCAAGCA

***RcUbq*** 247 ACATCCAGAAGGAGTCTACTTTGCATCTGGTGCTGCGATTGAGAGGAGGGGCGAAAAAGAGAAAGAAGAAGACGTACACCAAGCCCAAGAAGATCAAGCA

***StUbq*** 298 ACATCCAGAAGGAGTCAACTCTCCATCTCGTGCTCCGTCTCCGTGGTGGTGCTAAGAAGAGGAAGAAGAAGACCTACACCAAGCCAAAGAAGATCAAGCA

410 420 430 440 450 460 470 480 490 500

....|....|....|....|....|....|....|....|....|....|....|....|....|....|....|....|....|....|....|....|

***PtUbq*** 361 CAAGAAGAAGAAGGTCAAGCTCGCTGTGCTCCAGTTCTACAAGGTTGATGATAGCGGCAAAGTCCAGAGGTTGAGGAAGGAGTGCCCTAATGCTGAGTGC

***HbUbq*** 356 CAAGAAGAAGAAGGTCAAGCTCGCCATCCTTCAGTTCTACAAGGTTGATGATAGCGGCAAAGTGCAGAGGCTGAGGAAGGAGTGTCCTAACGCTGAGTGT

***MtUbq*** 369 CAAGCATAGGAAGGTGAAGCTTGCTGTTCTTCAGTTTTATAAGGTTGATGATTCTGGTAAGGTGCAGAGGTTGAGGAAGGAGTGTCCTAATGCTGAGTGT

***NtUbq*** 279 CAAGAAGAAGAAGGTTAAGCTCGCCGTCCTCCAGTTTTACAAGGTTGATGATTCCGGTAAGGTTCAGAGGCTCCGCAAGGAGTGTCCCAATGCTGAGTGT

***PcUbq*** 284 CAAGCACAAGAAGGTGAAGCTCGCAGTGCTCCAGTTCTACAAGGTGGATGACTCCCGGAAGGTCCAGAGGCTGCGGAAGGAGTGCCCCAATGCCGAGTGC

***RcUbq*** 347 CAAGAAGAAGAAGGTGAAGCTCGCTGTCCTTCAGTTTTACAAGGTCGATGATAGCGGAAAAGTGCAGAGGTTGAGGAAAGAGTGTCCGAACGCGGAGTGT

***StUbq*** 398 CAAGAAGAAGAAGGTTAAGCTCGCTGTGTTGCAGTTCTACAAGGTGGATGATACTGGAAAGGTTCAGAGGCTTCGTAAGGAGTGCCCTAATGCTGAGTGC

510 520 530 540 550 560 570 580 590 600

....|....|....|....|....|....|....|....|....|....|....|....|....|....|....|....|....|....|....|....|

***PtUbq*** 461 GGTGCTGGGACTTTCATGGCTAATCATTTTGATAGGCACTACTGTGGTAAGTGTGGGCTTACCTATGTTTACCAGACGGCTGGTGGTGA--TTAAGCTCA

***HbUbq*** 456 GGGGCTGGTACTTTCATGGCCAATCACTTTGATAGGCACTACTGTGGTAAGTGTGGCCTCACCTATGTTTACCAGAAGGCCGGTGGTGA--TTGA--TTG

***MtUbq*** 469 GGTGCTGGAACTTTTATGGCTAATCATTTTGATCGTCATTATTGTGGTAAGTGTGGTCTTACCTATGTTTACCAGAAGGCGGAAGCTTAGATTCAATGTT

***NtUbq*** 379 GGTGCCGGTTCTTTCATGGCTAACCACTTTGACAGGCACTATTGTGGTAAATGTGGGCTTACCTATGTTTACCAGAAGGCTGGTGGTGA--CTAG-----

***PcUbq*** 384 GGCGCCGGGACTTTCATGGCGAACCACTTCGACAGGCACTACTGCGGCAAGTGCGGGTTGACCTATGTTTACCAGAAGGCTGG--------CTGATTAGA

***RcUbq*** 447 GGTGCTGGCACTTTTATGGCTAATCATTTTGATAGGCACTACTGCGGTAAGTGTGGTCTTACTTATGTCTACCAGAAGGCTGGTGGTGA---TTAGGGCA

***StUbq*** 498 GGTGCTGGAACTTTTATGGCTAACCATTTCGACCGTCACTACTGTGGTAAGTGTGGGCTCACCTACGTTTACAACAAGGCTGGAGGCGA--TTGATTTTA

610 620 630 640 650 660 670 680 690 700

....|....|....|....|....|....|....|....|....|....|....|....|....|....|....|....|....|....|....|....|

***PtUbq*** 559 TTTGAATGGCGTTTTTGTCTTAAGTATGGAACAAGGAT-TATCTTTATTTAGAACATGGTTG---CTGT-TGAACTTA-TAGTTATGTTTTATTCGCATT

***HbUbq*** 552 ATTGATCGCCGCCGATGTCGCTTATAGGTTTTTGAGATGTCTTTTTATTTAAACTACCGTTGGACCTGT-TAAAGTTT-TGTTTCGATTATGGTAATTTC

***MtUbq*** 569 TCAA--TGATGATGTTCTAGTGTTTC--TGTTTTGTTATTGTTGTTGAACT---TTTTAATGTTTCAGTTTCGGTTTAATTAGCTTCGTTTGGAAAACAA

***NtUbq*** 471 ----------------------------------------------------------------------------------------------------

***PcUbq*** 476 GTAATTTGGAGTTTTTAATTTCGAATTATATCGCCATGGATTTTAAATTTTGATGCTTTATGGTGCTTT-TGGATTTT-AATTTCATGCTTGAAGAACCG

***RcUbq*** 544 ACAAATTTAAGTCCTTTGAGTACTATGTCATTTTGAGATTATTGTTGGACCAAGTATTATGGCATTTCTTTTGTTGTTATGAGTTTTGTTTGGATAATTT

***StUbq*** 596 ATG-TTTAGCAAATGTCTTATCAGTTTTCTTTTTTGTCGAACGGTAATTTAGA-GTTTTTTTTTGCTATATGGATTTT-CGTTT-----TTGATGTATAT

710 720 730 740 750 760 770 780 790 800

....|....|....|....|....|....|....|....|....|....|....|....|....|....|....|....|....|....|....|....|

***PtUbq*** 653 GCAAATTTTGAGTTTAATTATATTTTTGTTTTATATTGTCCCCCCACAAAAACCAAAAAAAAAAACC---------------------------------

***HbUbq*** 650 GGATACTACT-GTTGGATTGTGTTATTCTTAATTTAGATTTTTTTTAATCAATTGATGTTGTTGACCCTAAGTGATATGATTTTCGATGAAATTTTCGAA

***MtUbq*** 662 TTATTATATGTTCTAGTTGAATATCCAATACAATTTTGTGCGTTTTGAATTATGATTGTTACGTTGTTTTTTGCTGTTGGTTTTATGGTGATATTCTCTG

***NtUbq*** 471 ----------------------------------------------------------------------------------------------------

***PcUbq*** 574 ATGA-GGCTTTAGTTGTTTCTATTGCATATCCCAGATGGAATGATTACCCTAATTTTGTTATCAAAAAAAAAAAAAAAA---------------------

***RcUbq*** 644 TGGATGGTACTTCTTTTTGAAGTTATAATG-GATAATTAGTGGCTCATTTTATA----------------------------------------------

***StUbq*** 688 GTGACAACCCTCGGGATTGTTGATTTATTTCAAAACTAAGAGTTTTTGCTTATTGTTCTCGTCTATTTTGGATATCAAA---------------------

1. *Ef1α* gene*.* Species and accession numbers used: *Populus trichoparpa* (EF147714.1), *Arabidopsis thaliana* (NM_100666.3), *Elaeis guineensis* (AY550990.1), *Gossypium hirsutum* (DQ174254.1), *Malus domestica* (AJ223969.1)*, Nicotiana paniculata* (AB019427.1), *Prunus persica* (FJ267653.1)*, Vitis vinifera* (XM_002284888.1).

10 20 30 40 50 60 70 80 90 100

....|....|....|....|....|....|....|....|....|....|....|....|....|....|....|....|....|....|....|....|

***PtEF1a***  1 --------------GGTCATTAGCTACTCTCCTCCTTCCATCT--CTCTCGCGGC--CAGGGTTTAATCCATTCTCTCGTAAGTTCAGCTCTAATATATC

***AtEF1a***  1 AATAAAACCACTCTCGTTGCTGATTCCATTTATCGTTCTTATTGACCCTAGCCGCTACACACTTTTCTGCGATATCTCTGAGATTTGTTGACAGTCTCTA

***ElgEF1a*** 1 -----------------------GCTTGCCC--TGGTTCTTCTTTTTTGGGAGGGCTCAGTCGTTTC--CCACACACC-GCCGTCACGTC-CAAAAGCA-

***GhEF1a***  1 ----------------------------------------------------------------------------------------------------

***MdEF1a***  1 ----GCGCTCTTCACACTCTGAAGTCGGCGAGAGAAAGCTCCTGAATCTTCCTGTCGCTCTCGTCTG----TTTCTTCCAGT-TATTTTTCTGATTATCC

***NpEF1a***  1 ---------------------------GGCACGAGTCTCCATCTGCTCT-GCGGCAACAGATCTAAATTTGCTTTC---AAGTCCTTTTTTCAATC----

***PpEF1a***  1 -------------TGCTGCCTTCTCTTACTCACTGCCTCTGCCGCTCTGCTTGAACCTAG-CGTTTGAGCTTCAGATCTGTGGTAAATTT-TAATCGCTT

***VvEF1a***  1 -CTCGTCTCTCCCACATACCCTCTTCTGTTTGCTGTGTTCTCTCGCTGCGGCTAGGGTTTTAGCACGATATCTCCTTCTAACGCATCTTTTTAGGAATCC

110 120 130 140 150 160 170 180 190 200

....|....|....|....|....|....|....|....|....|....|....|....|....|....|....|....|....|....|....|....|

***PtEF1a***  83 ATCATGGGTAAGGAAAAGAGTCACATCAACATTGTGGTCATTGGCCATGTCGACTCTGGAAAATCAACCACCACTGGCCACTTGATCTACAAGCTTGGAG

***AtEF1a***  101 ACCATGGGTAAAGAGAAGTTTCACATCAACATTGTGGTCATTGGCCACGTCGATTCTGGAAAGTCGACCACCACTGGGCACTTGATCTACAAGTTGGGTG

***ElgEF1a*** 71 ACCATGGGTAAGGAGAAGGTCCATATCAACATTGTCGTCATTGGTCATGTTGACTCTGGCAAGTCGACCACCACCGGGCATCTCATTTACAAGCTTGGTG

***GhEF1a***  1 ---ATGGGTAAGGAGAAGGTTCACATCAACATTGTTGTCATTGGCCATGTTGACTCTGGAAAGTCAACCACAACGGGTCACTTGATATACAAGCTTGGAG

***MdEF1a***  92 AACATGGGTAAGGAGAAGTTCCACATCAACATCGTGGTCATTGGCCATGTCGACTCTGGGAAGTCGACCACGACAGGTCACTTGATCTACAAGCTTGGTG

***NpEF1a***  66 AACATGGGTAAAGAGAAGGTTCACATCAACATTGTGGTCATTGGCCATGTCGACTCTGGTAAATCAACTACCACTGGTCACTTGATCTACAAGCTTGGTG

***PpEF1a***  86 ATAATGGGCAAAGAAAAGTTTCACATCAACATCGTGGTCATTGGCCATGTCGACTCTGGAAAATCGACCACAACTGGTCATCTTATCTACAAGCTTGGAG

***VvEF1a***  100 ACAATGGGTAAAGAGAAGGTTCACATCAACATTGTCGTCATTGGCCATGTCGACTCTGGCAAGTCGACTACCACTGGTCACTTGATCTACAAGCTTGGAG

210 220 230 240 250 260 270 280 290 300

....|....|....|....|....|....|....|....|....|....|....|....|....|....|....|....|....|....|....|....|

***PtEF1a***  183 GTATTGACAAGCGTGTCATCGAGAGGTTCGAGAAGGAAGCTGCTGAGATGAACAAGAGGTCATTCAAGTATGCCTGGGTGCTCGACAAGCTCAAGGCTGA

***AtEF1a***  201 GTATTGACAAGCGTGTCATTGAGAGGTTCGAGAAGGAGGCTGCTGAGATGAACAAGAGGTCCTTCAAGTACGCATGGGTTTTGGACAAACTTAAGGCTGA

***ElgEF1a*** 171 GAATTGATAAGCGTGTGATTGAGAGATTTGAAAAGGAGGCTGCGGAAATGAACAAGAGGTCTTTCAAGTATGCATGGGTTTTGGACAAGCTGAAGGCTGA

***GhEF1a***  98 GTATTGACAAGCGTGTGATCGAGAGGTTCGAGAAGGAAGCTGCTGAGATGAACAAAAGGTCATTCAAGTATGCCTGGGTGCTCGACAAGTTGAAGGCTGA

***MdEF1a***  192 GTATTGACAAGCGTGTTATTGAGAGGTTCGAGAAGGAGGCAGCTGAGATGAACAAGAGGTCATTCAAGTATGCCTGGGTGTTGGACAAGCTCAAGGCTGA

***NpEF1a***  166 GTATTGACAAGCGTGTCATTGAGAGGTTTGAGAAAGAAGCTGCTGAGATGAACAAGAGGTCATTCAAGTATGCCTGGGTGCTTGACAAGCTAAAGGCTGA

***PpEF1a***  186 GTATTGACAAGCGTGTCATTGAGAGGTTCGAGAAGGAAGCTGCTGAGATGAACAAAAGGTCATTCAAGTACGCCTGGGTGCTTGACAAGCTTAAGGCTGA

***VvEF1a***  200 GTATTGACAAGCGTGTGATTGAGAGGTTTGAAAAGGAAGCGGCTGAGATGAACAAGAGGTCATTCAAGTATGCTTGGGTGTTGGACAAGCTGAAGGCTGA

310 320 330 340 350 360 370 380 390 400

....|....|....|....|....|....|....|....|....|....|....|....|....|....|....|....|....|....|....|....|

***PtEF1a***  283 GCGCGAGCGTGGTATCACCATTGACATTGCCTTGTGGAAGTTCGAGACCACCAAGTACTACTGCACTGTCATTGATGCCCCTGGACATCGTGACTTTATC

***AtEF1a***  301 GCGTGAGCGTGGTATCACCATTGACATTGCTCTCTGGAAGTTCGAGACCACCAAGTACTACTGCACTGTCATTGATGCTCCTGGCCATCGTGATTTCATC

***ElgEF1a*** 271 GCGTGAGCGTGGTATCACCATTGATATTGCTCTGTGGAAGTTTGAGACCACCAAGTACTACTGCACAGTCATTGATGCACCTGGTCATCGTGACTTCATT

***GhEF1a***  198 GCGTGAGCGTGGTATCACCATTGATATTGCCTTGTGGAAGTTTGAGACAACCAAGTACTACTGCACTGTCATTGATGCTCCTGGACATCGCGACTTTATT

***MdEF1a***  292 GCGTGAACGTGGTATTACCATTGACATTGCCCTGTGGAAGTTCGAGACCACCAAGTACTACTGCACTGTCATTGATGCTCCTGGACATCGTGACTTTATC

***NpEF1a***  266 GCGTGAGCGTGGTATCACTATTGATATTGCCTTGTGGAAGTTTGAGACCACCAAGTACTACTGCACTGTGATTGATGCTCCTGGACACAGGGATTTCATC

***PpEF1a***  286 GCGTGAGCGTGGTATCACCATTGATATTGCCTTGTGGAAGTTTGAGACCACCAAGTACTACTGCACAGTCATTGATGCCCCAGGACATCGTGACTTTATC

***VvEF1a***  300 GCGTGAACGTGGTATCACCATTGATATTGCCTTGTGGAAGTTTGAAACCACCAGGTACTACTGCACTGTTATTGATGCTCCTGGCCATCGGGACTTCATC

410 420 430 440 450 460 470 480 490 500

....|....|....|....|....|....|....|....|....|....|....|....|....|....|....|....|....|....|....|....|

***PtEF1a***  383 AAGAACATGATTACTGGGACTTCCCAGGCTGACTGTGCCGTGCTTATCATTGATTCCACCACTGGTGGTTTTGAAGCTGGTATCTCCAAGGATGGCCAGA

***AtEF1a***  401 AAGAACATGATCACTGGTACCTCCCAGGCTGATTGTGCTGTCCTTATCATTGACTCCACCACTGGTGGTTTTGAGGCTGGTATCTCCAAGGATGGTCAGA

***ElgEF1a*** 371 AAGAACATGATCACAGGAACCTCCCAGGCTGACTGTGCTGTCCTTATTATTGACTCCACTACTGGTGGTTTTGAGGCTGGTATATCAAAGGATGGGCAGA

***GhEF1a***  298 AAGAATATGATTACGGGTACTTCTCAAGCTGACTGTGCTGTCCTTATCATTGACTCCACAACTGGAGGTTTTGAAGCTGGTATTTCCAAGGATGGGCAGA

***MdEF1a***  392 AAGAACATGATTACTGGAACCTCACAGGCTGACTGTGCCATTCTCATCATTGACTCTACCACCGGAGGTTTTGAAGCCGGTATTTCCAAGGATGGTCAGA

***NpEF1a***  366 AAGAATATGATTACTGGTACCTCTCAAGCTGACTGTGCTGTCCTGATTATCGACTCTACCACTGGTGGTTTTGAAGCTGGTATCTCCAAGGATGGACAGA

***PpEF1a***  386 AAGAACATGATTACTGGAACCTCACAGGCTGACTGTGCTGTTCTCATCATCGATTCCACCACTGGTGGTTTTGAAGCTGGTATCTCCAAGGATGGCCAGA

***VvEF1a***  400 AAGAACATGATTACTGGTACCTCACAGGCAGATTGTGCTGTCCTCATTATTGACTCCACCACTGGTGGTTTTGAAGCTGGTATCTCCAAGGATGGACAAA

510 520 530 540 550 560 570 580 590 600

....|....|....|....|....|....|....|....|....|....|....|....|....|....|....|....|....|....|....|....|

***PtEF1a***  483 CCCGTGAGCACGCACTCCTTGCCTTCACCCTTGGTGTGAGGCAAATGATCTGCTGCTGTAACAAGATGGATGCCACAACTCCAAAGTACTCCAAGGCAAG

***AtEF1a***  501 CCCGTGAGCACGCTCTACTTGCTTTCACCCTTGGTGTCAAGCAGATGATCTGCTGTTGTAACAAGATGGATGCCACTACCCCCAAGTACTCCAAGGCCAG

***ElgEF1a*** 471 CCCGTGAGCATGCTTTGCTTGCCTTTACCCTTGGTGTGAAGCAGATGATTTGCTGTTGCAACAAGATGGATGCAACTACACCAAAGTACTCCAAGGCAAG

***GhEF1a***  398 CCCGTGAGCATGCTCTCCTTGCCTTCACCCTTGGTGTCAAGCAAATGATTTGCTGCTGCAACAAGATGGATGCCACAACCCCCAAGTACTCAAAGGCAAG

***MdEF1a***  492 CCCGTGAGCATGCTTTGCTTGCTTTTACTCTTGGTGTCAGGCAAATGATTTGCTGCTGCAACAAGATGGATGCCACCACTCCCAAGTACTCAAGGGCAAG

***NpEF1a***  466 CCCGTGAACATGCATTGCTTGCTTTCACCCTTGGTGTCAAACAAATGATTTGCTGCTGCAACAAGATGGATGCTACCACCCCCAAGTACTCCAAGGCTAG

***PpEF1a***  486 CCCGTGAGCATGCCCTTCTTGCTTTCACCCTTGGTGTGAAGCAGATGATTTGCTGCTGTAACAAGATGGATGCCACTACTCCCAAGTACTCCAAGGCAAG

***VvEF1a***  500 CCCGTGAGCATGCACTACTTGCTTTCACCCTTGGTGTGAAGCAGATGATTTGCTGCTGTAACAAGATGGATGCCACAACACCCAAGTACTCCAAGGCAAG

610 620 630 640 650 660 670 680 690 700

....|....|....|....|....|....|....|....|....|....|....|....|....|....|....|....|....|....|....|....|

***PtEF1a***  583 GTATGATGAAATTGTCAAGGAGGTGTCATCCTACTTGAAGAAGGTTGGTTACAACCCTGACAAGATTCCCTTTGTCCCCATCTCTGGATTTGAGGGTGAC

***AtEF1a***  601 GTACGATGAAATCATCAAGGAGGTGTCTTCCTACTTGAAGAAGGTTGGTTACAACCCCGACAAAATCCCATTTGTGCCCATCTCTGGATTTGAGGGTGAC

***ElgEF1a*** 571 GTATGATGAAATTGTTAAAGAAGTGTCCTCCTACCTGAAGAAGGTAGGTTACAATCCTGAGAAGATTCCTTTTGTTCCCATCTCCGGTTTTGAAGGTGAC

***GhEF1a***  498 GTATGATGAAATTGTTAAGGAAGTTTCTTCTTACCTGAAGAAGGTTGGTTACAACCCTGAGAAGATTCCATTCGTCCCCATCTCTGGTTTTGAGGGTGAC

***MdEF1a***  592 GTATGATGAAATTGTGAAGGAAGTGTCGTCCTATCTCAAGAAGGTTGGCTACAACCCAGATAAGATCCCCTTTGTCCCCATTTCTGGGTTCGAGGGTGAC

***NpEF1a***  566 GTACGATGAAATTGTGAAGGAGGTTTCTTCCTACCTCAAGAAGGTTGGATACAACCCTGACAAGATCCCCTTTGTCCCCATCTCTGGTTTTGAGGGTGAC

***PpEF1a***  586 GTACGATGAAATCGTGAAGGAAGTCTCATCCTATCTGAAGAAGGTTGGGTACAACCCGGACAAAATTGCCTTTGTTCCCATCTCTGGGTTCGAGGGTGAC

***VvEF1a***  600 GTACGATGAAATCGTGAAGGAAGTTTCTTCCTACCTGAAGAAGGTTGGATACAACCCTGATAAGATTCCATTTGTCCCCATCTCTGGCTTTGAGGGTGAC

710 720 730 740 750 760 770 780 790 800

....|....|....|....|....|....|....|....|....|....|....|....|....|....|....|....|....|....|....|....|

***PtEF1a***  683 AACATGATTGAGAGGTCCACCAACCTTGACTGGTACAAGGGCCCAACTCTCCTGGATGCCCTGGACCAGATCCAGGAGCCCAAGAGGCCCTCAGACAAGC

***AtEF1a***  701 AACATGATTGAGAGGTCCACCAACCTTGACTGGTACAAGGGACCAACTCTCCTTGAGGCTCTTGACCAGATCAACGAGCCCAAGAGGCCGTCAGACAAGC

***ElgEF1a*** 671 AACATGATCGAGAGGTCCACAAACTTAGATTGGTACAAGGGCCCAACACTTCTTGAGGCTCTTGACATGATCCAGGAGCCCAAGAGGCCCTCCGATAAGC

***GhEF1a***  598 AACATGATTGAGAGGTCCACCAACCTCGATTGGTACAAGGGTCCAACCCTCCTTGAGGCTCTTGACCAGATCAATGAGCCCAAGAGACCCTCTGACAAGC

***MdEF1a***  692 AACATGATTGAGAGGTCCACCAACCTTGACTGGTACAAGGGTCCCACCCTTCTTGAGGCTCTTGACCAGATCAATGAGCCCAAGAGGCCCTCAGACAAGC

***NpEF1a***  666 AACATGATCGAAAGATCAACCAACCTTGACTGGTACAAGGGCCCAACTCTTCTTGAGGCTCTTGACCAGATTAATGAGCCCAAGAGGCCCACAGACAAGC

***PpEF1a***  686 AACATGATTGAGAGATCCACCAACCTTGACTGGTACAAGGGACCAACCCTTCTTGAGGCTCTTGACTTGATCAATGAGCCCAAGAGGCCCTCAGACAAGC

***VvEF1a***  700 AATATGATAGAGAGGTCTACCAACCTTGACTGGTACAAGGGCCCAACTCTTCTTGAGGCCCTGGACATGATCAATGAGCCCAAGAGGCCCACAGACAAGC

810 820 830 840 850 860 870 880 890 900

....|....|....|....|....|....|....|....|....|....|....|....|....|....|....|....|....|....|....|....|

***PtEF1a***  783 CCCTCCGTCTCCCGCTTCAGGACGTGTACAAGATTGGTGGTATCGGAACTGTCCCAGTGGGTCGTGTTGAAACTGGTATCATCAAGCCCGGCATGGTTGT

***AtEF1a***  801 CCCTTCGTCTCCCACTTCAGGATGTCTACAAGATTGGTGGTATTGGAACGGTGCCAGTGGGACGTGTTGAGACTGGTATGATCAAGCCTGGTATGGTTGT

***ElgEF1a*** 771 CCCTTCGCCTCCCACTTCAGGATGTCTACAAGATTGGTGGCATTGGTACTGTCCCCGTTGGACGTGTTGAGACTGGTATCCTCAAGCCTGGTATGGTTGT

***GhEF1a***  698 CCCTCCGTCTCCCACTTCAGGATGTCTACAAGATTGGTGGTATTGGAACTGTCCCAGTGGGTCGTGTTGAAACTGGAATCCTCAAGCCTGGAATGGTTGT

***MdEF1a***  792 CCCTCCGGCTTCCACTTCAGGATGTGTACAAGATTGGTGGTATCGGTACTGTTCCTGTTGGACGTGTTGAGACTGGTGTCATTAAGCCTGGTATGGTGGT

***NpEF1a***  766 CCCTCAGGCTTCCACTTCAGGATGTTTACAAGATTGGTGGTATTGGTACTGTGCCCGTTGGTCGTGTGGAAACTGGTGTCCTCAAGCCTGGTATGCTTGT

***PpEF1a***  786 CTCTCCGTCTACCACTTCAGGATGTGTACAAGATTGGTGGTATTGGAACTGTGCCAGTGGGCCGTGTTGAGACCGGTATTATCAAGCCTGGTATGGTTGT

***VvEF1a***  800 CACTGCGACTCCCTCTTCAGGACGTGTACAAGATTGGTGGGATTGGAACTGTCCCAGTGGGACGTGTGGAGACTGGTGTCCTGAAGCCCGGTATGGTGGT

910 920 930 940 950 960 970 980 990 1000

....|....|....|....|....|....|....|....|....|....|....|....|....|....|....|....|....|....|....|....|

***PtEF1a***  883 CACATTCGGTCCAACTGGACTGAGTACTGAAGTCAAGTCTGTTGAGATGCACCACGAGGCTCTCCTAGAGGCACTTCCCGGTGACAATGTCGGGTTCAAT

***AtEF1a***  901 GACCTTTGCTCCCACAGGATTGACCACTGAGGTCAAGTCTGTTGAGATGCACCACGAGTCTCTTCTTGAGGCACTTCCAGGTGACAACGTTGGGTTCAAT

***ElgEF1a*** 871 CACCTTTGGCCCAAGTGGACTGACTACTGAAGTTAAATCTGTTGAGATGCACCATGAAGCCTTGCAAGAGGCTCTCCCTGGTGACAATGTAGGATTTAAC

***GhEF1a***  798 TACATTCGGACCTTCTGGATTGACCACTGAAGTTAAGTCTGTTGAGATGCATCATGAAGCTCTTCAAGAGGCTCTTCCTGGTGACAATGTTGGGTTCAAT

***MdEF1a***  892 GACTTTTGGCCCAACTGGTCTGACTACTGAGGTCAAGTCTGTTGAGATGCACCACGAAGCTATGCAGGAGGCCCTTCCAGGTGATAATGTTGGATTCAAC

***NpEF1a***  866 GACTTTTGGTCCCACTGGTCTGACCACTGAAGTTAAATCTGTTGAGATGCACCACGAAGCTCTTCAGGAGGCACTTCCTGGTGACAATGTTGGATTCAAC

***PpEF1a***  886 CACTTTTGGACCAACTGGGCTCACCACTGAAGTTAAGTCTGTAGAGATGCATCATGAGGCCCTTCAGGAGGCACTGCCTGGTGACAATGTCGGATTCAAT

***VvEF1a***  900 GACCTTTGGCCCCTCTGGACTGACAACTGAAGTCAAGTCTGTTGAGATGCACCATGAGTCTCTCCCAGAGGCTTTGCCTGGTGACAATGTTGGCTTCAAT

1010 1020 1030 1040 1050 1060 1070 1080 1090 1100

....|....|....|....|....|....|....|....|....|....|....|....|....|....|....|....|....|....|....|....|

***PtEF1a***  983 GTTAAGAATGTAGCTGTTAAGGATCTGAAGCGTGGTTTTGTTGCCTCGAACTCCAAGGACGATCCTGCCAAGGAGGCTGCCAACTTCACCGCCCAAGTTA

***AtEF1a***  1001 GTTAAGAATGTTGCTGTCAAGGATCTTAAGAGAGGGTACGTCGCATCCAACTCCAAGGATGACCCTGCCAAGGGTGCTGCTAACTTCACCTCCCAGGTCA

***ElgEF1a*** 971 GTTAAGAATGTTGCTGTCAAAGATCTCAAGCGTGGTTTTGTTGCCTCCAACTCAAAGGATGATCCTGCAAAGGAGGCTGCCAGCTTCACTTCTCAGGTCA

***GhEF1a***  898 GTGAAGAATGTTGCTGTCAAGGATCTCAAGCGTGGATTTGTTGCCTCCAACTCCAAGGATGATCCTGCCAAGGAGGCAGCCAACTTCACCTCCCAAGTTA

***MdEF1a***  992 GTTAAGAATGTTGCTGTCAAGGATCTCAAGCGTGGGTACGTTGCTTCCAACTCCAAGGATGATCCCGCAAAGGAGGCTGCCAACTTTATCGCTCAGGTCA

***NpEF1a***  966 GTCAAGAACGTTGCAGTTAAGGATCTCAAGCGTGGGTTTGTTGCTTCCAACTCCAAGGATGACCCAGCTAAGGGTGCTTCCAGCTTTACCTCCCAAGTCA

***PpEF1a***  986 GTTAAGAATGTTGCTGTGAAGGATCTCAAGCGTGGTTTCGTTGCATCTAACTCCAAGGATGATCCCGCCAGGGAGGCTGCGAACTTCACATCCCAGGTCA

***VvEF1a***  1000 GTGAAGAACGTTGCTGTGAAGGATCTCAAGCGTGGGTTTGTTGCCTCCAACTCCAAGGATGACCCTGCTAAGGAGGCAGCCAACTTCACCTCCCAGGTCA

1110 1120 1130 1140 1150 1160 1170 1180 1190 1200

....|....|....|....|....|....|....|....|....|....|....|....|....|....|....|....|....|....|....|....|

***PtEF1a***  1083 TCATCATGAACCATCCTGGGCAGATCGGAAACGGTTACGCCCCTGTTCTTGACTGCCACACCTGTCACATTGCTGTGAAGTTTGCTGAGATCCTCACCAA

***AtEF1a***  1101 TCATCATGAACCACCCTGGTCAGATTGGTAACGGTTACGCCCCAGTCCTGGATTGCCACACCTCTCACATTGCAGTCAAGTTCTCTGAGATCTTGACCAA

***ElgEF1a*** 1071 TCATCATGAATCACCCGGGTCAGATTGGTAATGGTTATGCCCCTGTGCTTGATTGCCACACCTCTCACATTGCTGTCAAATTCGCTGAGATCCTCACCAA

***GhEF1a***  998 TCATCATGAACCACCCAGGACAGATTGGAAATGGCTATGCACCGGTCCTCGATTGCCACACCTCCCATATTGCTGTCAAGTTTGCAGAGCTCTTGACCAA

***MdEF1a***  1092 TCATCATGAACCACCCCGGCCAGATTGGACAGGGATATGCTCCAGTTCTCGACTGTCACACCTCCCACATTGCCGTCAAGTTTGCTGAGCTCGTTACAAA

***NpEF1a***  1066 TCATCATGAACCATCCAGGACAGATTGGAAATGGATATGCTCCAGTGCTTGACTGCCACACCTCCCACATTGCTGTCAAGTTTGCAGAAATTTTGACCAA

***PpEF1a***  1086 TCATCATGAACCACCCTGGTCAGATTGGTAACGGATATGCTCCAGTTCTTGATTGCCACACTTCTCACATTGCTGTGAAGTTCGGTGAGATCCTCACCAA

***VvEF1a***  1100 TCATCATGAACCACCCGGGTCAGATCGGAAATGGCTATGCCCCTGTTCTGGACTGCCACACCTCCCACATTGCTGTTAAGTTTGCTGAGATACTGACCAA

1210 1220 1230 1240 1250 1260 1270 1280 1290 1300

....|....|....|....|....|....|....|....|....|....|....|....|....|....|....|....|....|....|....|....|

***PtEF1a***  1183 GATTGACAGGCGGTCTGGGAAGGAACTGGAGAAGGAGCCCAAGTTCCTGAAGAATGGTGATGCTGGTATGATTAAGATGATTCCCACCAAGCCCATGGTG

***AtEF1a***  1201 GATTGACAGGCGTTCTGGTAAGGAGATTGAGAAGGAGCCCAAGTTCTTGAAGAATGGTGATGCTGGTATGGTGAAGATGACTCCAACCAAGCCCATGGTT

***ElgEF1a*** 1171 GATTGACAGGCGATCTGGCAAGGAGCTTGAGAAGGAGCCTAAGTTCCTTAAGAATGGTGATGCTGGATTTGTGAAGATGATTCCCACCAAGCCTATGGTG

***GhEF1a***  1098 GATTGACAGGCGATCTGGTAAGGAGCTTGAGAAGGAGCCTAAGTTCTTGAAGAATGGTGATGCTGGTATGATTAAGATGGTTCCGACCAAGCCCATGGTT

***MdEF1a***  1192 GATCGACAGGCGATCTGGCAAGGAGCTTGAGAAGGAGCCCAAGTTTTTAAAGAATGGTGATGCTGGATTTGTGAAGATGCTTCCCACCAAGCCCATGGTT

***NpEF1a***  1166 GATCGACAGGCGTTCTGGTAAGGAGCTTGAGAAGGAGCCCAAGTTCTTGAAGAATGGTGATGCTGGTATGGTTAAGATGATTCCCACCAAGCCCATGGTT

***PpEF1a***  1186 GATTGACAGGAGGTCTGGTAAGGAGATTGAGAAGGAGCCCAAATTTTTGAAGAACGGAGATGCAGGTATGGTGAAGATGCTTCCCACCAAGCCCATGGTT

***VvEF1a***  1200 GATTGACAGGCGATCTGGCAAGGAGCTTGAGAAGGAGCCCAAGTTCTTGAAGAATGGTGATGCAGGGTTTGTTAAGATGATTCCAACCAAGCCCATGGTG

1310 1320 1330 1340 1350 1360 1370 1380 1390 1400

....|....|....|....|....|....|....|....|....|....|....|....|....|....|....|....|....|....|....|....|

***PtEF1a***  1283 GTGGAGTCTTTCTCAGAGTATCCTCCACTTGGTCGATTTGCTGTGAGGGACATGCGCCAGACCGTTGCTGTGGGTGTGATCAAGAGCGTGGAGAAGAAGG

***AtEF1a***  1301 GTGGAGACCTTCTCTGAGTACCCACCACTTGGACGTTTCGCTGTGAGGGACATGAGGCAGACTGTTGCAGTCGGTGTTATCAAGAGTGTTGACAAGAAGG

***ElgEF1a*** 1271 GTTGAGACTTTCTCTCAGTATCCTCCTCTTGGTCGTTTTGCTGTCAGAGACATGAGACAGACGGTGGCTGTGGGAGTCATCAAGAGTGTTGAGAAGAAGG

***GhEF1a***  1198 GTGGAAACTTTCTCCGAGTACCCTCCACTTGGACGTTTTGCCGTTAGGGACATGAGACAGACTGTTGCTGTTGGTGTGATCAAGAGTGTGGAGAAGAAGG

***MdEF1a***  1292 GTTGAGACCTTCTCTGAGTACCCACCGCTCGGACGTTTTGCTGTGAGGGACATGCGCCAGACTGTTGCAGTTGGTGTCATCAAGAGCGTTGAGAAGAAGG

***NpEF1a***  1266 GTTGAGACCTTCTCTGAGTATCCACCATTGGGACGTTTTGCTGTGAGGGACATGCGTCAAACTGTTGCTGTTGGTGTTATCAAGAACGTTGACAAGAAGG

***PpEF1a***  1286 GTGGAGACTTTCTCTGAGTACCCTCCATTGGGTCGTTTTGCTGTCCGTGACATGCGTCAGACTGTTGCTGTTGGTGTTATCAAGAGCGTGGAGAAGAAGG

***VvEF1a***  1300 GTGGAGACTTTCTCCGAGTATCCCCCACTTGGTCGATTTGCTGTTCGTGACATGCGTCAGACTGTTGCTGTTGGAGTCATCAAGAGCGTGGAGAAGAAGG

1410 1420 1430 1440 1450 1460 1470 1480 1490 1500

....|....|....|....|....|....|....|....|....|....|....|....|....|....|....|....|....|....|....|....|

***PtEF1a***  1383 AACCCTCTGGTGCCAAGGTGACCAAATCTGCTGCCAAGAAGG--------GTGGCAAGTGAACGGAGCAGGTTGATGCTACTGGAATCTATTT-TACAAA

***AtEF1a***  1401 ACCCAACCGGAGCCAAGGTTACCAAGGCTGCCGTCAAGAAGG--------GTGCGAAGTGAACCATCCTCAAAACTCTATCTGCCGCAGGTGA-ATCAAA

***ElgEF1a*** 1371 ACCCTAGTGGTGCCAAGGTCACCAAGTCTGCTGCAAAGAAGAA---------GTAAAGCTCGAGGCCATAAGGTA-TGAGCTACAACAGCCAT----AAT

***GhEF1a***  1298 ACCCAACTGGAGCCAAGGTGACGAAGTCTGCTGCCAAGAAGG-----------GCAAGTGAACCTCAGGGGATTTTGTTGATTCAACAACAGTTTATCAT

***MdEF1a***  1392 ATCCAACTGGAGCTAAGATCACCAAGGCCGCAGCTAAGAAGAAGTGAACCGCACTGGTTCAACAGATTTCCGCAGCGAAATCTTCTATCATCTTTATAAT

***NpEF1a***  1366 ACCCAACTGGTGCTAAGGTCACCAAGGCTGCTCAGAAGAAGAAAT-----GAACGTTGCAGTTCAATTCTGGTGTTACTTGTGATATCTACTACAATAAA

***PpEF1a***  1386 ATCCCAGTGGTGCCAAGGTCACCAAGGCTGCAGCAAAGAAGAA---------GTGAGATGTGTTTTACCGGGCCCCTGAGCTAGGCTAGTTTTGTCCAAT

***VvEF1a***  1400 ATCCATCTGGAGCCAAGGTCACCAAGTCTGCAGCCAAGAAGAAGTGAAGCGTGCGCAATGGG-AGCTCAACAGAG-GAAGCCTCCTATCATCAAAATAAA

1510 1520 1530 1540 1550 1560 1570 1580 1590 1600

....|....|....|....|....|....|....|....|....|....|....|....|....|....|....|....|....|....|....|....|

***PtEF1a***  1474 TAACGACTTGGT----TCTTCTTAGTTCTCAATTTCTTGGATGTTTATCCATGTGTCTTGATCTCTG-TAGTTTTATGTCACTACTCTCATCAAGAGGCT

***AtEF1a***  1492 GGACAGTGTTAG----TTTTATTA-----CAATAGTTTGG-TATTTGGTCGCGTGTCTGTGTTCTTG-TTTCGTTTTCTC------CCCGTCAGAGCGTT

***ElgEF1a*** 1457 GGAGCTCATCTTTCAAGTCATGTGATGGTT--TATCTTTCAAGTCATGTGAT----GGTGGTGTTGACCTGTGGTTAGGAATTAGGTTTTTGTATCAGTG

***GhEF1a***  1387 GGTTACTACAATAAAACTTTGGTTCATGTTAGTAGGTCCATCACTGGACGT------TTAGTTACATGTTTTAATGGTTCACCGTCATCTCTTGGATATT

***MdEF1a***  1492 AATATTAGTCGTAAAAAC---TTCTCTATTAGTATTTCTTTGGATTGCTGG----------TTGTGTATTTTCGTGT--CGGTGTCGTCCTTAGGCATTC

***NpEF1a***  1461 GTGTGTTATGGAGACATTGTTTTCTCTGCTTGTGGTGTTTTTGTTCGGTCTTTAATGTGGTTCTTAGGTTCTAGTTTTCTGCCGTGTATGTCTGGTATCT

***PpEF1a***  1477 GCTGTTT-CTTTTCAATTTGTTTGGTG--T--TATTATGGAAGTTTTGTG------GACAGTTTTGA---ATGTTTACATGCGTAGTGCTTCTACCAACC

***VvEF1a***  1498 ATGATTTGTCGTGAAAACATTTTATCTATTACTACTGTCATAGATCGTCCGC---TTTATTTTGTGCGTCATAGTTTTCTGCCTTCTTCCTTGGGTAGCC
