# Supplementary material for: Identification and validation of quantitative real-time reverse transcription PCR reference genes for gene expression analysis in teak (Tectona grandis L.f.)
Source: BMC Res Notes. 2014 Jul 22;7:464. doi: 10.1186/1756-0500-7-464 (PMC4114093; doi:10.1186/1756-0500-7-464)
Supplement: Additional file 3 — Protein clustal alignments for teak candidate reference genes. [file 1756-0500-7-464-S3.docx]

Additional File 3. Protein clustal alignments for teak candidate reference genes.

1. *rp60s* gene*.* Species and translated sequences used: *Tectona grandis* (JZ515972), *Populus trichoparpa* (XM_002300027.1), *Arabidopsis thaliana* (NM_117587.2), *Glycine max* (XM_003531057.1), *Pisum sativum* (U10046.1), *Ricinus communis* (XM_002513364.1), *Vitis vinifera* (XM_002277389.2)

10 20 30 40 50 60 70 80 90 100

....|....|....|....|....|....|....|....|....|....|....|....|....|....|....|....|....|....|....|....|

***TgRp60s*** 1 ---------ADMVKFLKPNKAVIILQGRYAGRKAVIVRSFDDGTRDRPYGHCLVAGLAKYPRKVIRKDSAKKQAKKSRVKCFIKLVNYNHIMPTRYTLDV

***PtRp60s*** 1 -----------MVKFLKTNKAVIILQGKYAGRKGVIVRSFDDGTRDRPYGHCLVAGIKKYPSKVIKKDSAKKTAKKSRVKCFIKLVNYQHLMPTRYTLDV

***GmRp60s*** 1 --FIHHQSRKKMVKFLKPNKAVIVLQGRYAGRKAVIVRTFDEGTRERPYGHCLVAGIKKYPSKVIKKDSAKKTAKKSRVKAFVKLVNYQHLMPTRYTFDV

***PsRp60s*** 1 --------GAKMVKFLKPNKAVILLQGRYAGKKAVIVKTFDDGTRDKPYGHCLVAGIKKYPSKVIKKDSAKKTAKKSRVKAFVKLVNYQHLMPTRYTLDV

***RcRp60s*** 1 --------EPKMVKFLKPNKAVILLQGRYAGRKAVIVRSFDDGTRDRPYGHCLVAGISKYPAKVIKKDSAKKTAKKSRVKAFMKVVNYSHLMPTRYTLDV

***VvRp60s*** 1 GFGLSLSLRAEMVKFLKQNKAVVVLQGRFAGRKAVIVRSFDDGTRDRPYGHCLVAGIAKYPKKVIRKDSAKKTAKKSRVKAFIKLVNYNHLMPTRYTLDV

110 120 130 140

....|....|....|....|....|....|....|....|....|.

***TgRp60s*** 92 DLKDVVAPDCLQSKDKKVTAAKETKARFEERFKTGKNRWFFTKL--

***PtRp60s*** 90 DLKDVVTADCLSTKDKKITACKETKARFEERFKTGKNRWFFTKLRF

***GmRp60s*** 99 DLKDAVTPDVLGTKDKKVTALKETKKRLEERFKTGKNRWFFTKLRF

***PsRp60s*** 93 DLKDAVVPDVLQSKDKKVTALKETKKSLEERFKTGKNRWFFTKLRF

***RcRp60s*** 93 DLKDVATPDALVTKDKKVTAAKEIKKRLEDRFKTGKNRWFFSKLRF

***VvRp60s*** 101 DLKDVVTVDALQSRDKKVTAAKETKARFEERFKTGKNRWFFTKLRF

1. *Cac* gene*.* Species and translated sequences used: *Tectona grandis* (JZ515973), *Vitis vinifera* (XM_002281392.1), *Arabidopsis lyrata* (XM_002894613.1), *Populus trichoparpa* (XM_002318903.1), *Ricinus communis* (XM_002512492.1), *Glycine max* (XM_003535990.1).

10 20 30 40 50 60 70 80 90 100

....|....|....|....|....|....|....|....|....|....|....|....|....|....|....|....|....|....|....|....|

***TgCac*** 1 ---------PGATSSCVPWRKTDLKHASNEVYVDLVEEMDATINRDGTLVKCEIYGEVQVNAHLSGLPDLTLLFANPSILNDVRFHPCVRLRPWESNQIL

***VvCac*** 1 NSSNVSNTLPGATASCVPWRSTEPKHANNEVYVDLLEEMDAVINRDGILVKCEIYGEVEVNSHLSGLPDLTLSFANPSILNDVRFHPCVRFRPWESNNIL

***AlCac*** 1 NASNVSDTLPSGAGSCVPWRPTDPKYSSNEVYVDLVEEMDAIVNRDGELVKCEIYGEVQMNSQLSGFPDLTLSFANPSILEDMRFHPCVRFRPWESHQVL

***PtCac*** 1 NSSNVSDTLPGATASCVPWRTTDIKYANNEVYVDLVEEMDAIINRDGVLVKCEIYGEVQVNSHITGVPELTLSFANPSIMDDVRFHPCVRFRPWESHHIL

***RcCac*** 1 NSSNVSDTLPNATSSCVPWRTTDVKYANNEVYVDLVEEMDAIINRDGVLMKCEIYGELQVNSHITGVPDLTLSFTNPSILDDVRFHPCVRFRPWESHQIL

***GmCac*** 1 SSSNVSDTLPGATASLVPWRTADTKYANNEVYVDLVEEMDATINRDGVLVKCEINGEVQVNSHITGLPDLTLSFANPSILDDVRFHPCVRYRPWESNQIL

110 120 130 140 150 160 170 180 190 200

....|....|....|....|....|....|....|....|....|....|....|....|....|....|....|....|....|....|....|....|

***TgCac*** 92 SFVPPDGQFNLMSYRVKKLKSTPIYVKPQLTSDSGTCRISVLVGIRNDPGKTIDSITVQFRLPPCVLSSDLSSNCGAVNVLADKTCSWTIGRIPKDKAPS

***VvCac*** 101 SFVPPDGQFKLMSYRVKKLRSTPIYVKPQLTSDAGTCRLSVLVGIRSDPGKTIDSVTVQFQLPPCILSANLSSNHGTVSILANKTCSWSIGRIPKDKAPS

***AlCac*** 101 SFVPPDGEFKLMSYRVKKLKNTPVYVKPQITSDAGTCRISVLVGIRSDPGKTIESITLSFQLPHCVSSADLSSNHGTVTILSNKTCTWTIGRIPKDKTPC

***PtCac*** 101 SFVPPDGLFKLMSYRVKKLKSTPIYVKPQITSDAGTCRINVMVGIRNDPGKMVDSITVQFQLPSCVLSADVTANHGAVTVFTNKMCNWSIDRIPKDRAPA

***RcCac*** 101 SFVPPDGLFKLMSYRVKKLKTVPIYVKPQLTSDAGTCRINLMVGIKNDPGKMIDSINVQFHLPPCILSADLTSNHGVVNVLSNKMCVWSIDRIPKDKTPS

***GmCac*** 101 SFVPPDGRFKLMSYRVGKLKNTPIYVKPQFTSDGGRCRVSVLVGIRNDPGKTIDNVTVQFQLPSCILSADLSSNYGIVNILANKICSWSIGRIPKDKAPS

210 220 230 240 250 260

....|....|....|....|....|....|....|....|....|....|....|....|....|

***TgCac*** 192 MSATLVLETGIERLHVFP-----------------------------------------------

***VvCac*** 201 LSGTLTLETGMERLHVFPTFQVGFRIMGVALSGLQIDTLDIKNLPSRPYKGFRALTQAGQYEVRS

***AlCac*** 201 LSGTLTLETGLERLHVFPTFKLGFKIMGIALSGLRIEKLDLQTIPPRLYKGFRAQTRAGEFDVRL

***PtCac*** 201 LSGTLMLETGLERLHVFPTFRVGFRIQGVALSGLQLDKLDLRVVPSRLYKGFRALTRSGLYEVRS

***RcCac*** 201 LSGTLVLETGLERLHVFPIFQLSFRIQGVALSGLQIDKLDLKVVPNRLYKGFRALTRAGLYEVRS

***GmCac*** 201 MSGTLVLETGLERLHVFPTFQVGFRIMGVALSGLQIDKLDLKTVPYRFYKGFRALTRAGEFEVRS

1. *Act* gene*.* Species and translated sequences used: *Tectona grandis* (JZ515974), *Populus trichoparpa* (XM_002308329.1), *Arabidopsis lyrata* (XM_002882721.1), *Arabidopsis thaliana* (NM_112046.3), *Glycine max* (NM_001254249.1), *Ricinus communis* (XM_002530665.1), *Vitis vinifera* (XM_002279636.1).

10 20 30 40 50 60 70 80 90 100

....|....|....|....|....|....|....|....|....|....|....|....|....|....|....|....|....|....|....|....|

***TgAct*** 1 -----------------------------------------------------------------------------VSNWDDMEKIWHHTFYNELRVAP

***PtACT*** 1 MAESEDIQPLVCDNGTGMVKAGFAGDDAPRAVFPSIVGRPRHTGVMVGMGQKDAYVGDEAQSKRGILTLKYPIEHGIVSNWDDMEKIWHHTFYNELRVAP

***AlACT*** 1 MADGEDIQPLVCDNGTGMVKAGFAGDDAPRAVFPSIVGRPRHTGVMVGMGQKDAYVGDEAQSKRGILTLKYPIEHGIVSNWDDMEKIWHHTFYNELRVAP

***AtACT*** 1 MADGEDIQPLVCDNGTGMVKAGFAGDDAPRAVFPSIVGRPRHTGVMVGMGQKDAYVGDEAQSKRGILTLKYPIEHGIVSNWDDMEKIWHHTFYNELRVAP

***GmACT*** 1 MADAEDIQPLVCDNGTGMVKAGFAGDDAPRAVFPSIVGRPRHTGVMVGMGQKDAYVGDEAQSKRGILTLKYPIEHGIVSNWDDMEKIWHHTFYNELRVAP

***RcACT*** 1 MADGEDIQPLVCDNGTGMVKAGFAGDDAPRAVFPSIVGRPRHTGVMVGMGQKDAYVGDEAQSKRGILTLKYPIEHGIVSNWDDMEKIWHHTFYNELRVAP

***VvACT*** 1 MAETEDIQPLVCDNGTGMVKAGFAGDDAPRAVFPSIVGRPRHTGVMVGMGQKDAYVGDEAQSKRGILTLKYPIEHGIVSNWDDMEKIWHHTFYNELRVAP

110 120 130 140 150 160 170 180 190 200

....|....|....|....|....|....|....|....|....|....|....|....|....|....|....|....|....|....|....|....|

***TgAct*** 24 EEHPILLTDAPLNPKANREKMTQIMFETFNAPAMYVAIQAVLSLYASGRTTGIVLDSGDGVSHTVPIYEGYALPHAILRLDLAGRDLTDHLMKILTERGY

***PtACT*** 101 EEHPVLLTEAPLNPKANREKMTQIMFETFNTPAMYVAIQAVLSLYASGRTTGIVLDSGDGVSHTVPIYEGYALPHAILRLDLAGRDLTDALMKILTERGY

***AlACT*** 101 EEHPVLLTEAPLNPKANREKMTQIMFETFNTPAMYVAIQAVLSLYASGRTTGIVLDSGDGVSHTVPIYEGYALPHAILRLDLAGRDLTDYLMKILTERGY

***AtACT*** 101 EEHPVLLTEAPLNPKANREKMTQIMFETFNTPAMYVAIQAVLSLYASGRTTGIVLDSGDGVSHTVPIYEGYALPHAILRLDLAGRDLTDYLMKILTERGY

***GmACT*** 101 EEHPVLLTEAPLNPKANREKMTQIMFETFNTPAMYVAIQAVLSLYASGRTTGIVLDSGDGVSHTVPIYEGYALPHAILRLDLAGRDLTDALMKILTERGY

***RcACT*** 101 EEHPVLLTEAPLNPKANREKMTQIMFETFNTPAMYVAIQAVLSLYASGRTTGIVLDSGDGVSHTVPIYEGYALPHAILRLDLAGRDLTDALMKILTERGY

***VvACT*** 101 EEHPVLLTEAPLNPKANREKMTQIMFETFNTPAMYVAIQAVLSLYASGRTTGIVLDSGDGVSHTVPIYEGYALPHAILRLDLAGRDLTDALMKILTERGY

210 220 230 240 250 260 270 280 290 300

....|....|....|....|....|....|....|....|....|....|....|....|....|....|....|....|....|....|....|....|

***TgAct*** 124 SFTTTAEREIVRDIKEKLAYIALDYEQELETAKTSSAVEKNYELPDGR----------------------------------------------------

***PtACT*** 201 SFTTTAEREIVRDMKEKLAYIALDYEQELETAKTSSSVEKSYELPDGQVITIGAERFRCPEVLFQPSMIGMEAAGIHETTYNSIMKCDVDIRKDLYGNIV

***AlACT*** 201 SFTTSAEREIVRDVKEKLSYIALDYEQEMDTANTSSSVEKSYELPDGQVITIGGERFRCPEVLFQPSLVGMEAAGIHETTYNSIMKCDVDIRKDLYGNIV

***AtACT*** 201 SFTTSAEREIVRDVKEKLAYIALDYEQEMETANTSSSVEKSYELPDGQVITIGGERFRCPEVLFQPSLVGMEAAGIHETTYNSIMKCDVDIRKDLYGNIV

***GmACT*** 201 TFTTSAEREIVRDMKEKLAYIALDYEQELETAKTSSAVEKSYELPDGQVITIGAERFRCPEVLFQPSMIGMESPGIHETTYNSIMKCDVDIRKDLYGNIV

***RcACT*** 201 SFTTTAEREIVRDMKEKLSYIALDYEQELETAKTSSSVEKSYELPDGQVITIGAERFRCPEVLFQPSMIGMEAAGIHETTYNSIMKCDVDIRKDLYGNIV

***VvACT*** 201 SFTTTAEREIVRDMKEKLAYIALDYEQELETAKTSSSVEKSYELPDGQVITIGAERFRCPEVLFQPSMIGMEAAGIHETTYNSIMKCDVDIRKDLYGNIV

1. *His3* gene*.* Species and translated sequences used: *Tectona grandis* (JZ515975), *Populus trichoparpa* (XM_002306258.1), *Gossypium hirsutum* (AF024716.1), *Lycopersicon esculentum* (X83422.1), *Zea mays* (EU976723.1).

10 20 30 40 50 60 70 80 90 100

....|....|....|....|....|....|....|....|....|....|....|....|....|....|....|....|....|....|....|....|

***TgHis3*** 1 --------------------------------TGGVKKPHRYRPGTVALREIRKYQKSTELLIRELPFQRLVREIAQDFKTDLRFQSHAVLALQEAAEAY

***PtHis3*** 1 MARTKQTARKSTGGKAPRKQLATKAARKSAPTTGGVKKPHRYRPGTVALREIRKYQKSTELLIRKLPFQRLVREIAQDFKTDLRFQSHAVLALQEAAEAY

***GhHis3*** 1 MARTKQTARKSTGGKAPRKQLATKAARKSAPTTGGVKKPHRYRPGTVALREIRKYQKSTELLIRKLPFQRLVREIAQDFKTDLRFQSHAVLALQEAAEAY

***LeHis3*** 1 MARTKQTARKSTGGKAPRKQLATKAARKSAPTTGGVKKPHRYRPGTVALREIRKYQKSTELLIRKLPFQRLVREIAQDFKTDLRFQSHAVLALQEAAEAY

***ZmHis3*** 1 MARTKQTARKSTGGKAPRKQLATKAARKSAPTTGGVKKPHRYRPGTVALREIRKYQKNTELLIRKLPFQRLVREIAQDFKTDLRFQSHAVLALQEAAEAY

110 120 130

....|....|....|....|....|....|....|.

***TgHis3*** 69 LVGLFEDTN---------------------------

***PtHis3*** 101 LVGLFEDTNLCAIHAKRVTIMPKDIQLARRIRGERA

***GhHis3*** 101 LVGLFEDTNLCAIHAKRVTIMPKDIQLARRIRGERA

***LeHis3*** 101 LVGLFEDTNLCAIHAKRVTIMPKDIQLARRIRGERA

***ZmHis3*** 101 LVGLFEDTNLCAIHAKRVTIMPKDIQLARRIRGERA

1. *Sand* gene*.* Species and translated sequences used: *Tectona grandis* (JZ515976), *Populus trichoparpa* (XM_002314230.1), *Arabidopsis thaliana* (NM_128399.3), *Picea sitchensis* (EF676351.1)*, Vitis vinifera* (XM_002285134.1).

10 20 30 40 50 60 70 80 90 100

....|....|....|....|....|....|....|....|....|....|....|....|....|....|....|....|....|....|....|....|

***TgSand*** 1 --------------------------------------HRCLG-ELMLSSLLSSILSVG-----------------------------------------

***PtSand*** 1 GKRHVDEDDASISWRKRKKHFFILSHSGKPIYSRYGDEHKLAGFSATLQAIISFVENGGDRVKLVRAGKHQVVFLVKGPIYLVCISCTEQPYESLRGELE

***AtSand*** 1 GKRHVDEDDASTSWRKRKKHFFILSNSGKPIYSRYGDEHKLAGFSATLQAIISFVENGGDRVNLVKAGNHQVVFLVKGPIYLVCISCTDETYEYLRGQLD

***PsSand*** 1 GKRYSNEDETSISWRKRKKHFFVLSHSGKPIYSRYGDEHKLAGFSATLQAIVSFVENGGDHIKLVRAGNHQIIFLVKGPIYLVCISCTEEPFQALKGQLE

***VvSand*** 1 GKRHVDEDDASISWRKRKKHFFILSHSGKPIYSRYGDEHKLAGFSATLQAIISFVENGGDRVQLIRAGKHQVVFLVKGPIYLVCISCTEEPYESLRSQLE

110 120 130 140 150 160 170 180 190 200

....|....|....|....|....|....|....|....|....|....|....|....|....|....|....|....|....|....|....|....|

***TgSand*** 20 -------------------------ILPLFRMPTHVFR-------------------LLMQHVKLQVP---LQDVAGSGVLFALLLCKHKVISLVGAQKA

***PtSand*** 101 LIYGQMILILTKSVNRCFEKNPKFDMTPLLGGTDVVFSSLIHSFSWNPATFLHAYTCLPLAYGTRQAAGAILHDVADSGVLFAILMCKHKVVSLVGAQKA

***AtSand*** 101 LLYGQMILILTKSIDRCFEKNAKFDMTPLLGGTDAVFSSLVHSFSWNPATFLHAYTCLPLPYALRQATGTILQEVCASGVLFSLLMCRHKVVSLAGAQKA

***PsSand*** 101 LLYDQMLLILTKSIDKCFEKNSKFDMTPLLGGTDVVFSSLIHAFSWNPATYLHAYTCLPLRHSTRQAAGAILQDVADSGVLFAILMCRHKVISLFGAQKA

***VvSand*** 101 LIYGQMLLILTKSVNRCFEKNPKFDMTPLLGGTDVVFSSLIHSFNWNPATFLHAYTCLPLAYATRQASGAILQDVADSGVLFAILMCKHKVISLVGAQKA

210 220 230 240 250 260 270 280 290 300

....|....|....|....|....|....|....|....|....|....|....|....|....|....|....|....|....|....|....|....|

***TgSand*** 74 SLHPDDILLLSNFIMSSESFR--------------TSESFSPICLPRYNSMAFLYAYVHYFDIDTYLILLTTSSDAFYHLKDSRIRIENVLLKSNVLSEV

***PtSand*** 201 SLHPDDMLLLSNFIMSSESFRQVKWTFIRLLLSHSTSESFSPICLPRYNPMAFLYAYVRYLDVDTYLM----------------IRIEMVLLKSNVLSEV

***AtSand*** 201 SLHPDDLLLLSNFVMSSESFR--------------TSESFSPICLPRYNAQAFLHAYVHFFDDDTYVILLTTRSDAFHHLKDCRVRLEAVLLKSNILSVV

***PsSand*** 201 ILHPDDMLLLSNFVLSSESFR--------------TSESFSPICLPQFNPMAFLYAYVQYLGVDTYLMLLTTDSDSFFHLKECRIRIENVLVKSNVLSEV

***VvSand*** 201 SLHPDDMLLLSNFVMSSESFR--------------TSESFSPICLPRYNPMAFLYAYVHYLDVDTYLMLLTTKSDAFYHLKDCRLRIETVLLKSNVLSEV

310 320 330 340 350 360 370 380 390 400

....|....|....|....|....|....|....|....|....|....|....|....|....|....|....|....|....|....|....|....|

***TgSand*** 160 QRSLVDGGMHIEDLLSDPASRPGAMSSHLGQPR-PGRDSPGRIRGGFVEIGGPAGLWHFM----------------------------------------

***PtSand*** 285 QRSMLDGGMHVEDLPADPLSRPGSASPHFGEHQ-EPTDSPRRFREPFAGIGGPAGLWHFIYRSIYLEQYISSEFSAPINSPQQQKRLYRAYQKLYASMHD

***AtSand*** 287 QRSIAEGGMRVEDVPIDRRRRSSTTNQEQ--------DSPGP--DISVGTGGPFGLWHFMYRSIYLDQYISSEFSPPVTSHRQQKSLYRAYQKLYASMHV

***PsSand*** 287 QRSMLDGCLRVEDLPGDPTLPSDSLSFRLRRDKNLQVAGSSTGTGRNTGIGGPAGLWHFMYRSNYLDQYVASEFSPPINSRNAQKRLFRAYQKLHTSMHD

***VvSand*** 287 QRSLLDGGMRVEDLPVDTSPRSGILSAHLGQHK-LPTDSPETSREECIGVGGPFGLWHFIYRSIYLDQYVSSEFSPPINSSRQQKRLYRAYQKLYASMHD

1. *Β-Tub* gene*.* Species and translated sequences used: *Tectona grandis* (JZ515977), *Populus trichoparpa* (XM_002298000.1), *Gossypium hirsutum* (AF521240.1), *Medicago truncatula* (XM_003630465.1), *Nicotiana tabacum* (EF051136.2), *Ricinus communis* (XM_002509755.1), *Theobroma cacao* (GU570572.1), *Vitis vinifera* (XM_002273478.2).

210 220 230 240 250 260 270 280 290 300

....|....|....|....|....|....|....|....|....|....|....|....|....|....|....|....|....|....|....|....|

***TgTub*** 1 -----------------------------------------------------------------------------------------TQQMWDAKNMM

***PtTub*** 55 VLDNEALYDICFRTLKLTNPSFGDLNHLISTTMSGVTCCLRFPGQLNSDLRKLAVNLIPFPRLHFFMVGFAPLTSQGSQQYRALTIPELTQQMWDAKNMM

***GhTub*** 201 VLDNEALYDICFRTLKLTNPSFGDLNHLISTTMSGVTCCLRFPGQLNSDLRKLAVNLIPFPRLHFFMVGFAPLTSRGSQQYRALTIPELTQQMWDSKNMM

***MtTub*** 201 VLDNEALYDICFRTLKLTNPSFGDLNHLISTTMSGVTCCLRFPGQLNSDLRKLAVNLIPFPRLHFFMVGFAPLTSRGSQQYSSLTIPELTQQMWDARNMM

***NtTub*** 55 VLDNEALYDICFRTLKLTTPSFGDLNHLISATMSGVTCCLRFPGQLNSDLRKLAVNLIPFPRLHFFMVGFAPLTSRGSQQYRALSVPELTQQMWDAKNMM

***RcTub*** 201 VLDNEALYDICFRTLKLTNPSFGDLNHLISTTMSGVTCCLRFPGQLNSDLRKLAVNLIPFPRLHFFMVGFAPLTSRGSQQYRALTIPELTQQMWDAKNMM

***TcTub*** 136 VLDNEALYDICFRTLKLTTPSFGDLNHLISATMSGVTCCLRFPGQLNSDLRKLAVNLIPFPRLHFFMVGFAPLTSRGSQQYRALTVPELTQQMWDAKNMM

***VvTub*** 201 VLDNEALYDICFRTLKLTNPSFGDLNHLISTTMSGVTCCLRFPGQLNSDLRKLAVNLIPFPRLHFFMVGFAPLTSRGSQQYRALTIPELTQQMWDAKNMM

310 320 330 340 350 360 370 380 390 400

....|....|....|....|....|....|....|....|....|....|....|....|....|....|....|....|....|....|....|....|

***TgTub*** 12 CAADPRHGRYLTASAMFRGKMSTKEVDEQMINVQNKNSSYFVEWIPNNVKSSVCDIPPTGLSMSSTFVGNSTSIQEMFRRVS------------------

***PtTub*** 155 CAADPRHGRYLTASAMFRGKMSTKEVDEQMMNVQNKNSSYFVEWIPNNVKSSVCDIPPTGLAMSSHIYG--------------KFYVYSRNV--------

***GhTub*** 301 CAADPRHGRYLTASAMFRGKMSTKEVDEQMINVQNKNSSYFVEWIPNDVKSSVCDIPPTGLTMSSTFMGNSTSIQEMFRRVSEQFTVMFRRKAFLHWYTG

***MtTub*** 301 CAADPRHGRYLTASAMFRGKMSTKEVDQQMINVQNKNSSYFVEWIPNNVKSSVCDIPPTGLSMSSTFMGNSTSIQEMFRRVSEQFTVMFKRKAFLHWYTA

***NtTub*** 155 CAADPRHGRYLTASAMFRGKMSTKEVDEQMLNVQNKNSSYFVEWIPNNVKSTVCDIPPTGLKMASTFIGNSTSIQEMFRRVSEQFTAMFRRKAFLHWYTG

***RcTub*** 301 CAADPRHGRYLTASAMFRGKMSTKEVDEQMINVQNKNSSYFVEWIPNNVKSSVCDIPPTGLSMSSTFMGNSTSIQEMFRRVSEQFTVMFRRKAFLHWYTG

***TcTub*** 236 CAADPRHGRYLTASAMFRGKMSTKEVDEQMINVQTKNSSYFVEWIPNNVKSSVCDIPPEGLSMASTFIGNSTSIQEMFRRVSEQFTAMFRRKAFLHWYTG

***VvTub*** 301 CAADPRHGRYLTASAMFRGKMSTKEVDEQMINVQNKNSSYFVEWIPNNVKSSVCDIPPTGLAMSSTFMGNSTSIQEMFRRVSEQFTVMFRRKAFLHWYTG

1. *Ubq* gene*.* Species and translated sequences used: *Tectona grandis* (JZ515978), *Populus trichoparpa* (XM_002320914.1), *Hevea brasiliensis* (EF120638.1), *Medicago truncatula* (XM_003629847.1), *Nicotiana tabacum* (DQ138111.1), *Pyrus communis* (AF386524.1), *Ricinus communis* (XM_002515167.1), *Solanum tuberosum* (L22576.1)

10 20 30 40 50 60 70 80 90 100

....|....|....|....|....|....|....|....|....|....|....|....|....|....|....|....|....|....|....|....|

***TgUbq*** 1 QQIDGDHNSGGILR-------HIDNVKAKIQDKEGIPPDQQRLIFAGKQLEDGRTLADYNIQKESTLHLVLRLRGGAKKRKKKTYTKPKKIKHKN-----

***HbUbq*** 1 MQIFVKTLTGKTITLEVESSDTIDNVKAKIQDKEGIPPDQQRLIFAGKQLEDGRTLADYNIQKESTLHLVLRLRGGAKKRKKKTYTKPKKIKHKKKKVKL

***MtUbq*** 1 MQIFVKTLTGKTITLEVESSDTIDNVKAKIQDKEGIPPDQQRLIFAGKQLEDGRTLADYNIQKESTLHLVLRLRGGAKKRKKKTYTKPKKIKHKHRKVKL

***NtUbq*** 1 MQIFVKTLTGKTITLEVESSDTIDNVKAKIQDKEGIPPDQQRLIFAGKQLEDGRTLADYNIQKESTLHLVLRLRGGAKKRKKKTYTKPKKIKHKKKKVKL

***PcUbq*** 1 MQIFVKTLTGKTITLEVESSDTIDNVKAKIQDKEGIPPDQQRLIFAGKQLEDGRTLADYNIQKESTLHLVLRLRGGAKKRKKKTYTKPKKIKHKHKKVKL

***RcUbq*** 1 MQIFVKTLTGKTITLEVESSDTIDNVKAKIQDKEGIPPDQQRLIFAGKQLEDGRTLADYNIQKESTLHLVLRLRGGAKKRKKKTYTKPKKIKHKKKKVKL

***StUbq*** 1 MQIFVKTLTGKTITLEVESSDTIDNVKAKIQDKEGIPPDQQRLIFAGKQLEDGRTLADYNIQKESTLHLVLRLRGGAKKRKKKTYTKPKKIKHKKKKVKL

1. *Ef1α* gene*.* Species and translated sequences used: *Tectona grandis* (JZ515979), *Populus trichoparpa* (EF147714.1), *Arabidopsis thaliana* (NM_100666.3), *Elaeis guineensis* (AY550990.1), *Gossypium hirsutum* (DQ174254.1), *Malus domestica* (AJ223969.1)*, Nicotiana paniculata* (AB019427.1), *Prunus persica* (FJ267653.1)*, Vitis vinifera* (XM_002284888.1).

110 120 130 140 150 160 170 180 190 200

....|....|....|....|....|....|....|....|....|....|....|....|....|....|....|....|....|....|....|....|

***TgEF1a***  9 ------SQADCAVLIIDSTTGGFEAGISKDGQTREHALLAFTLGVKQMICCCNKMDATTPKYSKARYDEIVKEVSSYLKKVGYNPEKIPFVPISGFEGDN

***PtEF1a***  101 NMITGTSQADCAVLIIDSTTGGFEAGISKDGQTREHALLAFTLGVRQMICCCNKMDATTPKYSKARYDEIVKEVSSYLKKVGYNPDKIPFVPISGFEGDN

***AtEF1a***  101 NMITGTSQADCAVLIIDSTTGGFEAGISKDGQTREHALLAFTLGVKQMICCCNKMDATTPKYSKARYDEIIKEVSSYLKKVGYNPDKIPFVPISGFEGDN

***ElgEF1a*** 101 NMITGTSQADCAVLIIDSTTGGFEAGISKDGQTREHALLAFTLGVKQMICCCNKMDATTPKYSKARYDEIVKEVSSYLKKVGYNPEKIPFVPISGFEGDN

***GhEF1a***  101 NMITGTSQADCAVLIIDSTTGGFEAGISKDGQTREHALLAFTLGVKQMICCCNKMDATTPKYSKARYDEIVKEVSSYLKKVGYNPEKIPFVPISGFEGDN

***MdEF1a***  101 NMITGTSQADCAILIIDSTTGGFEAGISKDGQTREHALLAFTLGVRQMICCCNKMDATTPKYSRARYDEIVKEVSSYLKKVGYNPDKIPFVPISGFEGDN

***NpEF1a***  101 NMITGTSQADCAVLIIDSTTGGFEAGISKDGQTREHALLAFTLGVKQMICCCNKMDATTPKYSKARYDEIVKEVSSYLKKVGYNPDKIPFVPISGFEGDN

***PpEF1a***  101 NMITGTSQADCAVLIIDSTTGGFEAGISKDGQTREHALLAFTLGVKQMICCCNKMDATTPKYSKARYDEIVKEVSSYLKKVGYNPDKIAFVPISGFEGDN

***VvEF1a***  101 NMITGTSQADCAVLIIDSTTGGFEAGISKDGQTREHALLAFTLGVKQMICCCNKMDATTPKYSKARYDEIVKEVSSYLKKVGYNPDKIPFVPISGFEGDN

210 220 230 240 250 260 270 280 290 300

....|....|....|....|....|....|....|....|....|....|....|....|....|....|....|....|....|....|....|....|

***TgEF1a***  104 MIERSTNLDWYKGPTLLEALDMVQEPKRPSDKPLRLPLQDVYKIGGIGTVPVGRVETGILKPGMVVTFGPTGLTTEVKSVEMHHEALQEALPGDNVGFNV

***PtEF1a***  201 MIERSTNLDWYKGPTLLDALDQIQEPKRPSDKPLRLPLQDVYKIGGIGTVPVGRVETGIIKPGMVVTFGPTGLSTEVKSVEMHHEALLEALPGDNVGFNV

***AtEF1a***  201 MIERSTNLDWYKGPTLLEALDQINEPKRPSDKPLRLPLQDVYKIGGIGTVPVGRVETGMIKPGMVVTFAPTGLTTEVKSVEMHHESLLEALPGDNVGFNV

***ElgEF1a*** 201 MIERSTNLDWYKGPTLLEALDMIQEPKRPSDKPLRLPLQDVYKIGGIGTVPVGRVETGILKPGMVVTFGPSGLTTEVKSVEMHHEALQEALPGDNVGFNV

***GhEF1a***  201 MIERSTNLDWYKGPTLLEALDQINEPKRPSDKPLRLPLQDVYKIGGIGTVPVGRVETGILKPGMVVTFGPSGLTTEVKSVEMHHEALQEALPGDNVGFNV

***MdEF1a***  201 MIERSTNLDWYKGPTLLEALDQINEPKRPSDKPLRLPLQDVYKIGGIGTVPVGRVETGVIKPGMVVTFGPTGLTTEVKSVEMHHEAMQEALPGDNVGFNV

***NpEF1a***  201 MIERSTNLDWYKGPTLLEALDQINEPKRPTDKPLRLPLQDVYKIGGIGTVPVGRVETGVLKPGMLVTFGPTGLTTEVKSVEMHHEALQEALPGDNVGFNV

***PpEF1a***  201 MIERSTNLDWYKGPTLLEALDLINEPKRPSDKPLRLPLQDVYKIGGIGTVPVGRVETGIIKPGMVVTFGPTGLTTEVKSVEMHHEALQEALPGDNVGFNV

***VvEF1a***  201 MIERSTNLDWYKGPTLLEALDMINEPKRPTDKPLRLPLQDVYKIGGIGTVPVGRVETGVLKPGMVVTFGPSGLTTEVKSVEMHHESLPEALPGDNVGFNV

310 320 330 340 350 360 370 380 390 400

....|....|....|....|....|....|....|....|....|....|....|....|....|....|....|....|....|....|....|....|

***TgEF1a***  204 KNVAVKDLKRGFVASNSKDDPAKEAANFTSQVIIMNHPGQIG----------------------------------------------------------

***PtEF1a***  301 KNVAVKDLKRGFVASNSKDDPAKEAANFTAQVIIMNHPGQIGNGYAPVLDCHTCHIAVKFAEILTKIDRRSGKELEKEPKFLKNGDAGMIKMIPTKPMVV

***AtEF1a***  301 KNVAVKDLKRGYVASNSKDDPAKGAANFTSQVIIMNHPGQIGNGYAPVLDCHTSHIAVKFSEILTKIDRRSGKEIEKEPKFLKNGDAGMVKMTPTKPMVV

***ElgEF1a*** 301 KNVAVKDLKRGFVASNSKDDPAKEAASFTSQVIIMNHPGQIGNGYAPVLDCHTSHIAVKFAEILTKIDRRSGKELEKEPKFLKNGDAGFVKMIPTKPMVV

***GhEF1a***  301 KNVAVKDLKRGFVASNSKDDPAKEAANFTSQVIIMNHPGQIGNGYAPVLDCHTSHIAVKFAELLTKIDRRSGKELEKEPKFLKNGDAGMIKMVPTKPMVV

***MdEF1a***  301 KNVAVKDLKRGYVASNSKDDPAKEAANFIAQVIIMNHPGQIGQGYAPVLDCHTSHIAVKFAELVTKIDRRSGKELEKEPKFLKNGDAGFVKMLPTKPMVV

***NpEF1a***  301 KNVAVKDLKRGFVASNSKDDPAKGASSFTSQVIIMNHPGQIGNGYAPVLDCHTSHIAVKFAEILTKIDRRSGKELEKEPKFLKNGDAGMVKMIPTKPMVV

***PpEF1a***  301 KNVAVKDLKRGFVASNSKDDPAREAANFTSQVIIMNHPGQIGNGYAPVLDCHTSHIAVKFGEILTKIDRRSGKEIEKEPKFLKNGDAGMVKMLPTKPMVV

***VvEF1a***  301 KNVAVKDLKRGFVASNSKDDPAKEAANFTSQVIIMNHPGQIGNGYAPVLDCHTSHIAVKFAEILTKIDRRSGKELEKEPKFLKNGDAGFVKMIPTKPMVV
