# Supplementary material for: Identification and validation of quantitative real-time reverse transcription PCR reference genes for gene expression analysis in teak (Tectona grandis L.f.)
Source: BMC Res Notes. 2014 Jul 22;7:464. doi: 10.1186/1756-0500-7-464 (PMC4114093; doi:10.1186/1756-0500-7-464)
Supplement: Additional file 4 — Teak sequences (with accession numbers) used for designing qRT-PCR primers (Underlined and bolded). [file 1756-0500-7-464-S4.docx]

Additional File 4. Teak sequences (with accession numbers) used for designing qRT-PCR primers (Underlined and bolded).

>*Tectona grandis ribosomal protein 60S* (JZ515972)

GCAGATATGGTGAAGTTCTTGAAGCCGAACAAAGCCGTAATAATCCTGCAAGGCCGTTACGCCGGCCGTAAAGCAGTGATCGTCCGCTCATTCGACGACGGCACTCGTGACCGGCCGTACGGCCATTGCTTGGTGGCGGGGCTGGCGAAGTACCCGCGCAAGGTCATCCGCAAAGACTCGGCGAAGAAGCAGGCGAAGAAATCACGAGTGAAATGCTTCATCAAATTGGTGAATTACAACCACATCATGCCCACGCGCTACACGCTCGATGTGGATCTGAAGGATGTGGTCGCTCCGGATTGCTTGCAGTCGAAAGATAAGAAGGTGACGGCGGCGAAGGAGACGAAGGCTCGGTTCGAGGAGCGGTTCAAGACGGGGAAAAACCGCTGGTTCTTTACCAAGCTC

>*Tectona grandis Clathrin Adaptor Complex* (JZ515973)

CCAGGGGCAACATCATCTTGTGTTCCATGGAGAAAAACAGACCTTAAGCATGCCAGCAATGAAGTTTATGTTGATCTTGTGGAAGAAATGGATGCCACTATAAACAGAGATGGGACTCTGGTAAAATGCGAGATATATGGTGAAGTTCAAGTAAATGCTCATTTATCAGGTCTCCCAGATCTCACTCTGTTGTTTGCGAACCCTTCAATTCTTAATGATGTGAGATTTCACCCTTGTGTTAGACTTCGTCCATGGGAATCAAACCAAATTCTGTCCTTTGTGCCACCTGATGGACAATTTAATCTCATGAGTTACAGGGTAAAGAAGTTGAAGAGTACTCCAATTTATGTGAAGCCGCAATTGACCTCGGATTCAGGGACATGTCGTATAAGTGTTCTAGTTGGAATACGGAACGATCCTGGAAAGACGATTGACTCTATAACAGTTCAATTCCGATTACCACCTTGTGTTTTATCGTCTGATCTTTCATCAAATTGTGGAGCTGTAAATGTTCTTGCTGACAAGACCTGCTCATGGACAATCGGACGAATACCAAAAGATAAAGCTCCTTCAATGTCTGCGACCTTGGTGCTTGAGACAGGCATAGAGCGCCTTCATGTATTTCCC

>*Tectona grandis Actin* (JZ515974)

GTTAGCAATTGGGATGATATGGAGAAAATCTGGCATCACACATTCTACAACGAACTTCGTGTTGCTCCAGAAGAGCACCCAATTCTCTTGACAGATGCTCCTCTTAATCCCAAGGCCAACCGTGAAAAGATGACTCAAATTATGTTTGAGACCTTTAATGCCCCTGCCATGTATGTTGCCATCCAGGCTGTTCTCTCCCTTTATGCCAGTGGTCGTACAACTGGTATTGTTCTCGACTCTGGGGATGGTGTTAGCCATACAGTGCCCATCTATGAAGGCTATGCACTCCCCCATGCTATCCTGCGTCTTGATCTTGCCGGGCGTGATCTCACTGACCACCTCATGAAGATATTGACAGAACGAGGCTACTCATTCACTACAACTGCAGAGCGGGAAATTGTAAGGGACATAAAAGAAAAGCTGGCTTACATTGCTCTGGATTATGAGCAAGAGCTAGAAACAGCAAAGACTAGCTCTGCTGTGGAGAAGAACTATGAACTGCCTGATGGCAGG

>*Tectona grandis Histone 3* (JZ515975)

ATTGGTGTCTTCGAAGAGACCAACAAGATACGCCTCCGCAGCTTCCTGGAGAGCGAGCACGGCGTGGCTTTGGAACCTCAAATCGGTCTTGAAATCCTGAGCGATTTCACGAACCAAACGCTGGAAAGGCAGCTCGCGGATGAGGAGTTCTGTGCTCTTCTGATATTTCCGGATTTCACGAAGAGCAACAGTTCCAGGGCGGTAACGATGGGGCTTCTTCACTCCTCCCGT

>*Tectona grandis Sand Family* (JZ515976)

CATCGCTGCTTGGGGGAACTGATGCTGTCTTCTCTTCTCTCATCCATTCTTTCAGTTGGAATCCTGCCACTTTTCCGCATGCCTACTCATGTCTTCCGCTTGCTTATGCAACACGTCAAGCTGCAGGTGCCTTTGCAAGATGTAGCTGGTTCAGGAGTCCTATTTGCACTCTTATTGTGTAAACACAAGGTTATCAGTCTTGTTGGCGCCCAAAAAGCATCTCTTCATCCTGATGATATATTGCTACTCTCCAATTTTATTATGTCCTCTGAATCTTTTAGGACATCTGAGTCCTTCTCACCAATTTGCCTGCCAAGATACAATTCCATGGCATTTCTTTATGCTTATGTGCATTATTTTGATATCGATACTTACCTGATCTTGCTCACCACAAGTTCTGATGCCTTTTATCATTTAAAAGATAGCAGGATTCGGATTGAAAATGTCCTTTTAAAGTCAAATGTACTGAGTGAAGTTCAAAGATCCTTGGTAGATGGTGGTATGCATATTGAGGATTTGCTTAGTGACCCCGCTTCTCGTCCTGGGGCCATGTCTTCTCATCTAGGTCAACCAAGACCTGGTAGAGATTCTCCGGGGAGAATTAGAGGTGGATTTGTTGAAATTGGTGGTCCAGCTGGACTTTGGCATTTCATG

>*Tectona grandis Beta Tubulin* (JZ515977)

ACACAGCAAATGTGGGATGCAAAGAACATGATGTGCGCCGCCGACCCCCGCCACGGCCGCTACCTGACCGCCTCTGCCATGTTCCGCGGCAAGATGAGCACGAAAGAAGTGGACGAACAAATGATCAACGTGCAGAACAAGAATTCATCCTACTTCGTCGAATGGATCCCCAACAACGTTAAATCAAGTGTTTGTGACATTCCCCCAACTGGGCTCTCAATGTCGTCGACTTTCGTCGGGAATTCGACGTCGATACAGGAGATGTTCCGGCGCGTGTCG

>*Tectona grandis Ubiquitin* (JZ515978)

TTCAGCAGATTGACGGGTAAGACCATAACTCTGGAGGTTGAATCCTCCGACATATCGACAATGTCAAGGCCAAGATCCAGGACAAGGAAGGCATACCGCCGGACCAGCAGCGCCTCATCTTCGCTGGAAAACAGCTCGAGGACGGTCGCACCCTCGCCGACTACAACATCCAAAAGGAATCGACTCTCCACCTCGTTCTCCGCCTCCGCGGCGGCGCTAAGAAGCGGAAGAAGAAGACCTACACCAAGCCGAAGAAAATCAAGCACAAGAACA

>*Tectona grandis Elongation Factor 1 alpha* (JZ515979)

CTTTATCAAGAACATGATCACTGGTTATCACAGGCTGATTGTGCTGTCCTTATCATTGACTCTACCACTGGTGGTTTTGAAGCTGGTATTTCCAAGGATGGTCAGACCCGTGAGCATGCATTGCTTGCTTTCACTCTTGGTGTCAAGCAAATGATTTGTTGTTGCAACAAGATGGATGCCACCACACCAAAATACTCCAAGGCTAGGTATGATGAAATTGTGAAGGAAGTGTCTTCCTACCTCAAGAAGGTTGGATACAACCCTGAAAAGATCCCATTTGTTCCCATTTCTGGTTTTGAGGGAGACAACATGATTGAGAGGTCCACTAACTTGGACTGGTACAAGGGCCCAACCCTCCTTGAGGCGCTTGACATGGTTCAGGAGCCCAAGAGGCCCTCAGACAAGCCTCTCCGTCTGCCACTTCAGGATGTTTACAAGATTGGTGGTATTGGTACTGTCCCTGTTGGCCGAGTGGAGACCGGTATCCTCAAGCCTGGTATGGTTGTGACCTTTGGCCCGACTGGGTTGACCACTGAAGTTAAGTCTGTTGAGATGCACCACGAAGCGTTGCAGGAGGCTCTTCCTGGTGATAATGTTGGGTTCAACGTGAAGAATGTTGCTGTGAAGGATCTGAAACGTGGTTTTGTTGCCTCCAACTCTAAGGATGATCCTGCTAAGGAAGCCGCCAACTTCACTTCCCAAGTCATCATCATGAACCACCCTGGCCAGATTGGA

> *Tectona grandis Glyceraldehyde 3-phosphate dehydrogenase* (FN431983.1)

GTACTTTATTCACCATCTGAGTCTAATAAATGCTGCTCGCCGACTTCACCTTGTGATTCTGCTTTCTAGTAGTGTTATCCTTTTGTTTGCATTTGTGGTAGTATATTTTAAGGAGATGGATTTGGATTTACTTGGTCTTCCCTTGATCTCTGTTTTGTACATAATTTGTGTAGGCTGTTGGTAAAGTGCTTCCAGCCCTAAATGGAAAGTTGACTGGTATGGCATTCCGAGTTCCAACAGTTGATGTTTCAGTCGTGGACCTCACTGTCAGGTTGGAGAAAGAGGCCACCTATGAGGAGATCAAAGCTGCCATCAAGTGAGTTTTTGAATTTGGTTTTGAACAATGTGAGCTAGTAATAGTTCTATGGATGTGTAATCTAAAGTTGCTTGGTTCTCTTAGGGAGGAGTCGGAGAACAAGCTAAAGGGCATCTTGGGGTACACTGAAGATGATGTGGTGTCAACAGACTTTGTGGGCGATAGCCGGTAAATGTCTTTTCCTAATGGAGTACCTTTTGGTCTAGCAATTAGTCCTCCTTCGATTTGTAGGGTGCTCAAACTT

>*Tectona grandis Cinnamyl Alcohol Dehydrogenase* (JZ515980)

AGCCATGAAGTAATTGGAGAAGTTGTTGAATTGGGCTCAAAAGTGAAGAATTTCAAAGTGGGTGACATTATAGGAGTTGGAGGAATTATTGGTTCTTGTGAAAAATGTACTCTCTGCAACTCCAATCTGGAGCAATACTGCAGCAACAGAATCTTTACCTACAATGACGTCTACAAAGATGGAACTCCAACTCAAGGGGGATATTCTTCTGCTATGGTTATTCATCACAGGTTTCCGAAATTTATCTTATTACATAAAAGCAAAAAAACACGAAACCGAAAACACTCAACAACGGATTACAGATTTGCAGTTAAAATACCAGAAAAACTAGCACCAGAACAAGCAGCACCACTACTATGTGCCGGGGTGACAGCATACAGTCCCCTCAAAGAGTTCATGGATTCCGGCAAGGTCTACAAAGGAGGAATATTAGGCTTGGGAGGAGTTGGTCACCTGGCTGTGATGATAGCAAAGGCAATGGGTCATCATGTGACAGTAATAAGCTCTTCTGATAAGAAAAAAGAGGAGGCTATGGAGCATTTGCACGCAGACGCCTTCTTGGTGAGTCGTAGTGAGGATGAAATGAAGCAAGCGATAAACAGCCTCGACTATATACTCGACACCGTGCCTGTTGTTCATCCTCTGCCATCATATATTTCACTTTTGAAAACTGAAGGAAAGCTGTTATTAGTAGCGGCAGTTCCTCAGCCACTTCAGTTTCTGGCTGCCGATATGATAAGAGGTATGGTATAT
