# Supplementary material for: Identification and validation of quantitative real-time reverse transcription PCR reference genes for gene expression analysis in teak (Tectona grandis L.f.)
Source: BMC Res Notes. 2014 Jul 22;7:464. doi: 10.1186/1756-0500-7-464 (PMC4114093; doi:10.1186/1756-0500-7-464)

Additional File 5. Agarose gel (2%) electrophoresis showing amplification of a specific PCR product of the expected size for each gene. M represents 50 bp DNA ladder marker (GeneRuler^TM^ 50bp DNA Ladder, Thermo Scientific, USA) and “-” represents negative control.


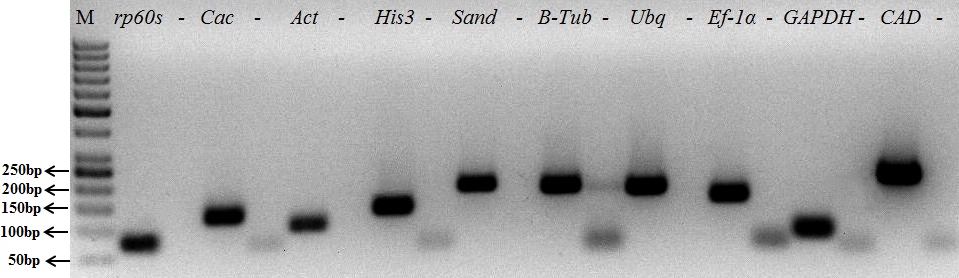

Supplement: Additional file 5 — Agarose gel (2%) electrophoresis showing amplification of a specific PCR product of the expected size for each gene. M represents 50 bp DNA ladder marker (GeneRuler™ 50 bp DNA Ladder, Thermo Scientific, USA) and “-” represents negative control. [file 1756-0500-7-464-S5.docx]
