# Supplementary material for: Identification and validation of quantitative real-time reverse transcription PCR reference genes for gene expression analysis in teak (Tectona grandis L.f.)
Source: BMC Res Notes. 2014 Jul 22;7:464. doi: 10.1186/1756-0500-7-464 (PMC4114093; doi:10.1186/1756-0500-7-464)
Supplement: Additional file 6 — Raw CP data used for statistical analysis in this study. [file 1756-0500-7-464-S6.docx]

Additional File 6. Raw CP data used for statistical analysis in this study.

| Sample | ***rp60s*** | ***Sand*** | ***Act*** | ***Cac*** | ***GAPDH*** | ***Β-Tub*** | ***His3*** | ***Ubq*** | ***Ef-1α*** |
| --- | --- | --- | --- | --- | --- | --- | --- | --- | --- |
| Flower 1 | 29.159 | 30.163 | 24.925 | 31.887 | 25.974 | 28.741 | 27.770 | 26.510 | 23.046 |
| Flower 2 | 27.611 | 29.992 | 24.479 | 30.733 | 25.414 | 28.699 | 27.055 | 26.141 | 22.920 |
| Flower 3 | 27.806 | 28.285 | 24.262 | 29.968 | 24.860 | 28.916 | 27.701 | 26.037 | 22.520 |
| Flower 4 | 28.983 | 30.218 | 24.658 | 32.597 | 26.103 | 28.841 | 27.572 | 26.526 | 23.098 |
| Flower 5 | 27.521 | 29.653 | 24.388 | 30.178 | 25.310 | 28.644 | 27.077 | 26.171 | 22.813 |
| Flower 6 | 27.816 | 28.664 | 24.151 | 29.691 | 24.992 | 28.855 | 27.466 | 25.987 | 22.558 |
| Leaf 1 | 29.820 | 30.447 | 27.498 | 32.098 | 26.482 | 28.904 | 27.554 | 27.794 | 24.410 |
| Leaf 2 | 28.787 | 29.239 | 27.401 | 30.424 | 25.626 | 31.931 | 26.306 | 26.993 | 24.220 |
| Leaf 3 | 28.847 | 29.426 | 25.849 | 30.955 | 25.932 | 28.147 | 26.543 | 26.642 | 22.884 |
| Leaf 4 | 29.714 | 30.536 | 27.201 | 32.032 | 26.533 | 28.920 | 27.565 | 27.796 | 24.241 |
| Leaf 5 | 28.888 | 29.037 | 27.158 | 29.953 | 25.635 | 32.017 | 26.560 | 26.976 | 24.194 |
| Leaf 6 | 28.947 | 29.728 | 25.793 | 31.299 | 25.972 | 28.013 | 26.550 | 26.585 | 22.893 |
| Root 1 | 27.900 | 29.061 | 25.016 | 30.590 | 25.232 | 28.649 | 26.951 | 26.033 | 22.504 |
| Root 2 | 28.797 | 29.801 | 25.394 | 30.971 | 25.647 | 29.283 | 27.399 | 26.722 | 23.162 |
| Root 3 | 28.319 | 29.789 | 25.790 | 31.423 | 25.802 | 29.618 | 28.157 | 26.823 | 23.336 |
| Root 4 | 27.875 | 29.049 | 24.948 | 30.242 | 25.382 | 28.661 | 26.860 | 25.993 | 22.545 |
| Root 5 | 28.821 | 29.923 | 25.515 | 30.612 | 25.691 | 29.088 | 27.414 | 26.616 | 23.021 |
| Root 6 | 28.343 | 29.660 | 25.877 | 31.403 | 25.968 | 29.766 | 27.980 | 26.789 | 23.407 |
| Seedling 1 | 27.679 | 30.160 | 24.804 | 31.406 | 24.638 | 29.793 | 29.507 | 26.157 | 22.903 |
| Seedling 2 | 28.409 | 30.059 | 25.111 | 31.592 | 24.971 | 29.144 | 28.428 | 26.533 | 22.845 |
| Seedling 3 | 28.719 | 30.913 | 24.926 | 32.432 | 25.728 | 28.731 | 28.702 | 26.399 | 22.926 |
| Seedling 4 | 27.769 | 30.357 | 24.760 | 31.513 | 24.797 | 29.603 | 29.396 | 26.176 | 22.874 |
| Seedling 5 | 28.273 | 29.673 | 25.068 | 31.469 | 25.032 | 29.083 | 28.465 | 26.480 | 22.855 |
| Seedling 6 | 28.875 | 30.627 | 24.855 | 32.940 | 25.877 | 28.684 | 28.541 | 26.398 | 22.970 |
| Branch secondary xylem 1 | 28.913 | 28.801 | 26.878 | 29.477 | 24.611 | 28.849 | 28.580 | 26.752 | 23.388 |
| Branch secondary xylem 2 | 28.306 | 28.550 | 26.326 | 29.944 | 25.181 | 28.444 | 28.676 | 26.069 | 23.077 |
| Branch secondary xylem 3 | 28.150 | 28.344 | 26.288 | 28.714 | 24.246 | 28.503 | 28.359 | 26.054 | 22.987 |
| Branch secondary xylem 4 | 28.888 | 29.133 | 26.981 | 29.350 | 24.737 | 28.792 | 28.569 | 26.677 | 23.563 |
| Branch secondary xylem 5 | 28.461 | 28.631 | 26.388 | 29.671 | 25.219 | 28.554 | 28.508 | 26.092 | 23.015 |
| Branch secondary xylem 6 | 28.064 | 28.084 | 25.574 | 28.817 | 24.205 | 27.996 | 28.266 | 26.135 | 22.968 |
| Stem secondary xylem 1 | 29.278 | 31.939 | 29.238 | 32.102 | 26.675 | 31.952 | 31.437 | 27.756 | 25.094 |
| Stem secondary xylem 2 | 28.957 | 31.716 | 29.907 | 32.036 | 26.662 | 33.539 | 28.851 | 28.706 | 25.134 |
| Stem secondary xylem 3 | 29.341 | 31.953 | 29.431 | 33.113 | 27.356 | 32.131 | 31.054 | 28.038 | 25.230 |
| Stem secondary xylem 4 | 29.059 | 32.147 | 29.301 | 32.545 | 26.540 | 32.107 | 31.451 | 27.897 | 24.926 |
| Stem secondary xylem 5 | 28.934 | 31.965 | 29.855 | 31.908 | 26.749 | 32.632 | 28.889 | 28.566 | 25.156 |
| Stem secondary xylem 6 | 29.277 | 31.991 | 29.723 | 32.445 | 27.361 | 32.242 | 31.658 | 27.945 | 25.164 |
